# Genes with stable DNA methylation levels show higher evolutionary conservation than genes with fluctuant DNA methylation levels

**DOI:** 10.18632/oncotarget.5504

**Published:** 2015-10-19

**Authors:** Ruijie Zhang, Wenhua Lv, Meiwei Luan, Jiajia Zheng, Miao Shi, Hongjie Zhu, Jin Li, Hongchao Lv, Mingming Zhang, Zhenwei Shang, Lian Duan, Yongshuai Jiang

**Affiliations:** ^1^ College of Bioinformatics Science and Technology, Harbin Medical University, Harbin 150086, China

**Keywords:** DNA methylation stability, evolutionary characteristics, SNP density, linkage disequilibrium

## Abstract

Different human genes often exhibit different degrees of stability in their DNA methylation levels between tissues, samples or cell types. This may be related to the evolution of human genome. Thus, we compared the evolutionary conservation between two types of genes: genes with stable DNA methylation levels (SM genes) and genes with fluctuant DNA methylation levels (FM genes). For long-term evolutionary characteristics between species, we compared the percentage of the orthologous genes, evolutionary rate dn/ds and protein sequence identity. We found that the SM genes had greater percentages of the orthologous genes, lower dn/ds, and higher protein sequence identities in all the 21 species. These results indicated that the SM genes were more evolutionarily conserved than the FM genes. For short-term evolutionary characteristics among human populations, we compared the single nucleotide polymorphism (SNP) density, and the linkage disequilibrium (LD) degree in HapMap populations and 1000 genomes project populations. We observed that the SM genes had lower SNP densities, and higher degrees of LD in all the 11 HapMap populations and 13 1000 genomes project populations. These results mean that the SM genes had more stable chromosome genetic structures, and were more conserved than the FM genes.

## INTRODUCTION

DNA methylation is an epigenetic mechanism [1, 2] that plays important roles in the regulation of gene expression [3, 4], development [5–7], X-chromosome inactivation [8] and genomic imprinting [9, 10].

Some genomic regions can exhibit different methylation statuses among multiple samples (tissues, cell types or individuals) [11]. Differences in DNA methylation levels can identify a methylation locus associated with susceptibility to diseases, such as Alzheimer's disease [12, 13], cardiovascular disease [14, 15], and cancer [16, 17]. Other genomic regions may exhibit robust or stable methylation statuses between different tissues, samples or cell types.

To explore the genetic basis of maintaining DNA methylation levels, we investigated the relationship between evolutionary conservation and the stability of DNA methylation levels. In this study, two types of genes were considered: SM genes were defined as genes that have stable DNA methylation levels in all cell types of every tissue under normal or disease conditions. In other words, the DNA methylation statuses of SM genes were robust to environmental changes. FM genes were defined as genes that have fluctuant DNA methylation levels in different cell types, tissues, organisms or samples; i.e., FM genes were sensitive to environmental changes. For SM genes and FM genes, we compared the long-term evolutionary characteristics and short-term evolutionary characteristics. We found a strong association between the stability of DNA methylation levels and evolutionary conservation.

## RESULTS

To make the results more robust, we used a unified platform (GPL13534) and a large sample size (8,676 samples). For each of the 21,231 genes, we employed a fluctuation coefficient, FC, to measure the stability of DNA methylation levels. We then constructed two gene sets, SM genes (4,247 genes) and FM genes (4,247 genes), and compared their differences in evolutionary conservation.

### SM genes had a higher percentage of orthologous genes than FM genes

**Figure 1:**
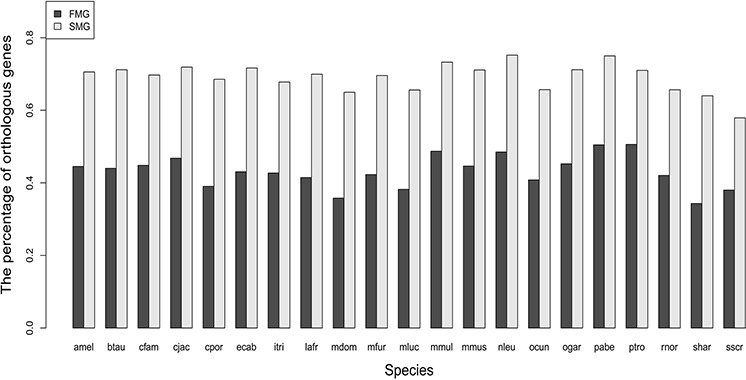
A bar chart comparing the percentage of orthologous genes between SM and FM genes

For SM and FM genes, we calculated the numbers of the one-to-one orthologous genes in each of the 21 species. The results are displayed as a bar chart in Figure 1, which shows that the bar chart of the SM genes is higher than that of the FM genes. We also used the Wilcoxon signed rank test to test the statistical significance. The null hypothesis is that the SM genes and the FM genes had the same percentage of orthologous genes in each of the 21 species. The *P*-value was 9.54E-07 and the significance level α was 0.05. This indicated that the SM genes had a higher percentage of orthologous genes than FM genes across the 21 species.

### SM genes had a lower evolutionary rate than FM genes

**Figure 2:**
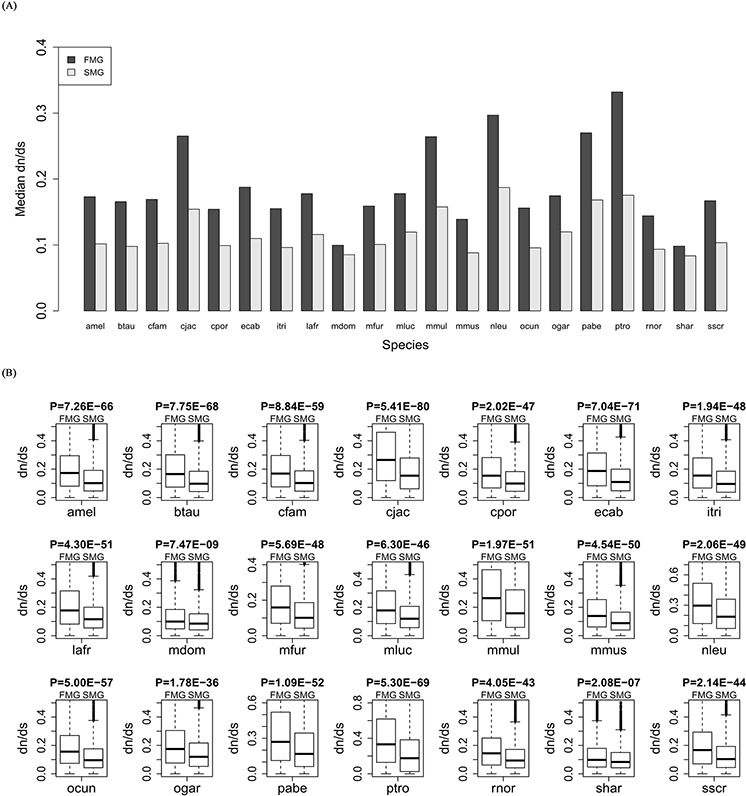
Comparison of the evolutionary rate, dn/ds, between SM and FM genes **A.** A bar chart of the SM genes against the FM genes. **B.** Box plots of the SM genes against the FM genes for each of the 21 species.

For each of the 21 species, we calculated the evolutionary rate dn/ds for orthologous genes of the SM and FM genes. The bar chart of the SM genes is lower than that of the FM genes (Figure 2A). A Wilcoxon signed rank test gave a significant *P*-value (*P* = 6.41E-05). Thus, the SM genes had lower dn/ds than the FM genes across all species. For each species, we also drew a box plot of the SM genes against the FM genes (Figure 2B). A Wilcoxon rank sum test produced significant *P*-values for each of the 21 species (for details, see Supplementary Table S1). These results indicated that the SM genes had a lower evolutionary rate than the FM genes.

### SM genes had a higher protein sequence identity than FM genes

**Figure 3:**
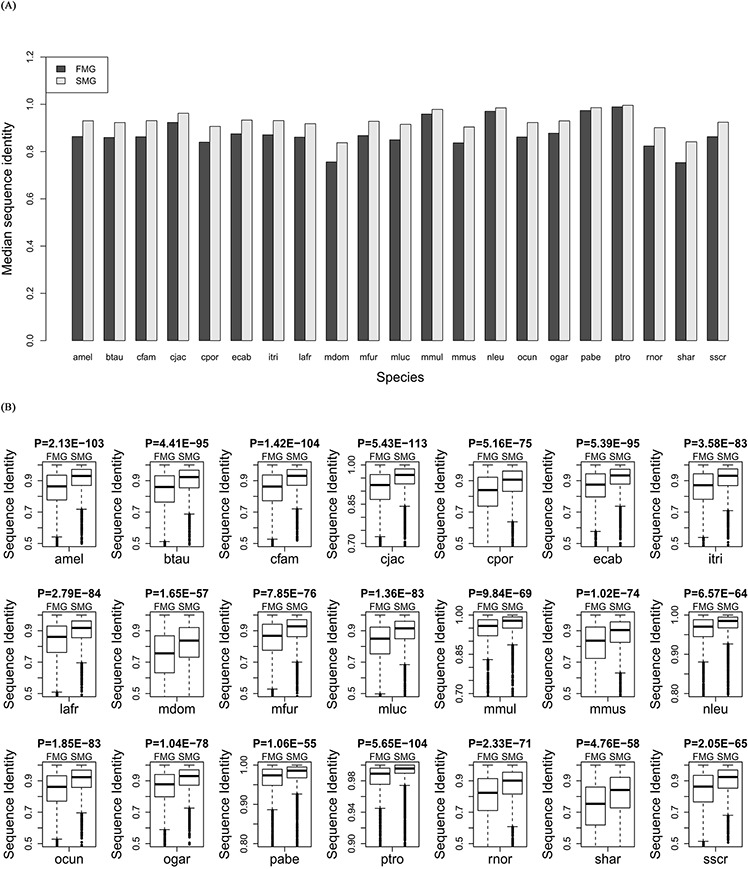
Comparison of the sequence identity between SM and FM genes **A.** A bar chart of the SM genes against the FM genes. **B.** Box plots of the SM genes against the FM genes for each of the 21 species.

We aligned the orthologous proteins between human and the other 21 species using BLASTP software. For SM and FM genes, we extracted the sequence identity from the alignment results. Comparing the medians (Figure 3A) showed that the SM genes had a higher protein sequence identity across all species (Wilcoxon signed rank test, *P* = 6.41E-05). Furthermore, we also compared the protein sequence identity between SM genes and FM genes in each of the 21 species. The protein sequence identity of SM genes was significantly higher than that of the FM genes for each species (Figure 3B). This also indicated that the SM genes were more conserved than the FM genes. Supplementary Table S2 showed the detailed information about the sequence identity for each species.

### SM genes had a lower SNP density than FM genes

**Figure 4:**
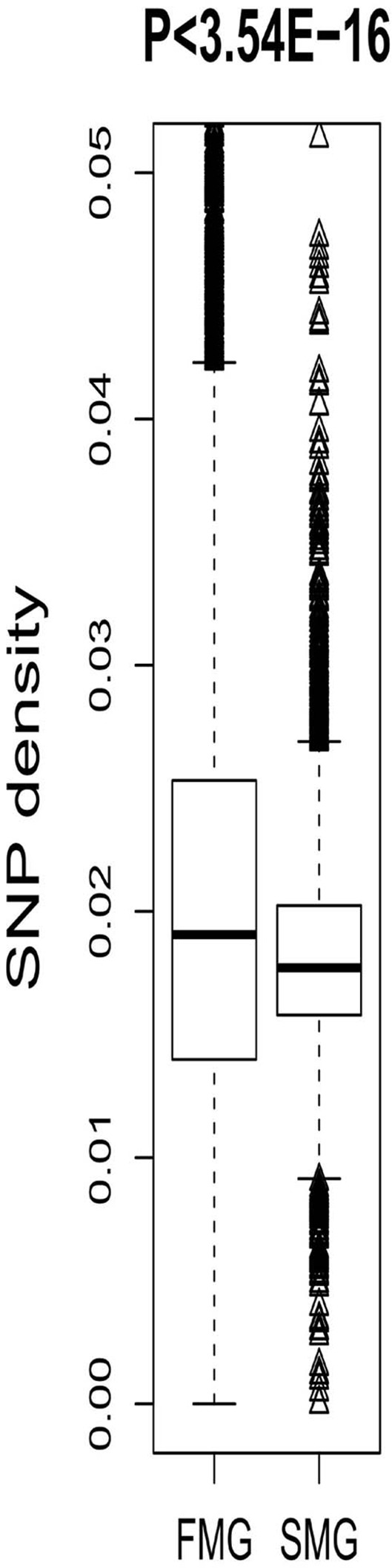
A box plot comparing the SNP density between SM and FM genes

We also compared some short-term evolutionary characteristics of the SM and FM genes. In the human genome, the most common single base genetic variation is the SNP [18, 19]. In this study, the SNP density in a gene region was used to measure the degree of genetic variation. The FM genes had higher SNP densities than the SM genes (Figure 4). A Wilcoxon rank sum test produced a significant *P*-value (*P* < 3.54E-16). This indicated that the FM genes contained significantly more genetic variations than the SM genes, i.e., the SM genes were more conservative.

### SM genes had a higher degree of LD in all 11 HapMap populations and 13 1000 genomes populations

**Figure 5:**
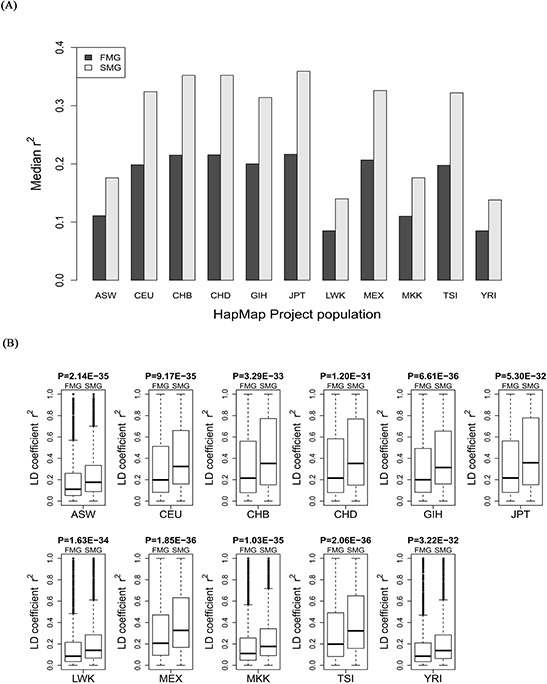
Comparison of the r^2^ value between SM and FM genes **A.** A bar chart of the SM genes against the FM genes. **B.** Box plots of the SM genes against the FM genes for each of the 11 HapMap populations.

**Figure 6:**
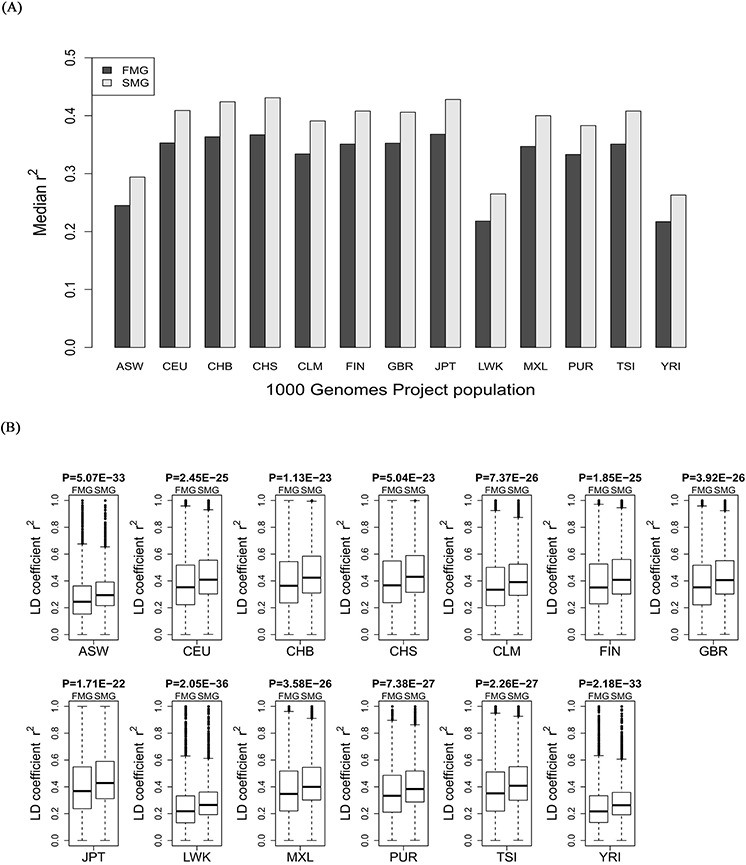
Comparison of the r^2^ value between SM and FM genes **A.** A bar chart of the SM genes against the FM genes. **B.** Box plots of the SM genes against the FM genes for each of the 13 1000 genomes project populations.

For each of the 11 HapMap and 13 1000 genomes populations, we calculated the LD coefficient, r^2^, for the SM and FM genes. We also drew a bar chart of the median r^2^ of the SM genes against the FM genes across all the HapMap populations (Figure 5A) and all the 1000 genomes populations (Figure 6A). Whether in HapMap populations or in 1000 genome populations, the bar chart of the SM genes was higher than that of the FM genes. Figure 5B and Figure 6B show that the *P*-values were significant for all the 11 HapMap populations and all the 13 1000 genomes populations. For details, see Supplementary Table S3 (the median, upper and lower quartiles of r^2^ for HapMap populations) and Supplementary Table S4 (the median, upper and lower quartiles of LD coefficient r^2^ for 1000 genomes populations). Thus, the SM genes had a higher degree of LD than the FM genes. Compared with FM genes, SM genes had undergone fewer recombination events, and had more stable chromosome genetic structures, i.e., the SM genes were more conserved.

### SM genes had different biological functions comparing with FM genes

We found that, for biological process (BP), SM genes were mainly annotated to certain metabolic processes that are critical to growth, reproduction and maintenance of structures and survival of cells, such as cellular metabolic process (GO:0044237, BP), cellular macromolecule metabolic process (GO:0044260, BP), metabolic process (GO:0008152, BP), macromolecule metabolic process (GO:0043170, BP), and primary metabolic process (GO:0044238, BP). For molecular function (MF), SM genes were mainly annotated to certain basic binding categories, such as nucleic acid binding (GO: 0003676, MF), RNA binding (GO: 0003723, MF) and binding (GO: 0005488, MF). These processes or functions are important for maintaining the stability of cells or organisms. FM genes were mainly annotated to processes (BP) or functions (MF) that interact with the environment, such as sensory perception of smell (GO: 0007608, BP), sensory perception of chemical stimulus (GO: 0007606, BP), G-protein coupled receptor protein signaling pathway (GO: 0007186, BP), sensory perception (GO: 0007600, BP) cognition (GO: 0050890, BP), olfactory receptor activity (GO: 0004984), G-protein coupled receptor activity (GO: 0004930, MF), transmembrane receptor activity (GO: 0004888, MF), receptor activity (GO: 0004872, MF) and molecular transducer activity (GO: 0060089, MF). These annotation results hint at the biological reasons underlying the differences in evolutionary conservation. Genes that maintain the basic survival of the cell tend to have stable DNA methylation levels. For details, see Supplementary File Section 2 (the GO annotation results of SM genes, from Supplementary Table 2-1 to Supplementary Table 2-3) and Supplementary File Section 3(the GO annotation results of FM genes, from Supplementary Table 3-1 to Supplementary Table 3-3).

## DISCUSSION

To ensure the reliability of the results, we first chose a large sample set (8,676 samples) to identify the SM and FM genes. Then we compared three long-term (species) evolutionary characteristics (the percentage of the orthologous genes, evolutionary rate (dn/ds) and protein sequence identity) and two short-term (human) evolutionary characteristics (the SNP density and LD degree) between the SM and FM genes. We came to the following six conclusions: (1) the SM genes had a greater percentage of the orthologous genes than FM genes for all the 21 species; (2) the SM genes had a lower evolutionary rate than the FM genes for all 21 species; (3) the SM genes had a higher protein sequence identity than FM genes for all 21 species; (4) the SM genes had a lower SNP densities than the FM genes; (5) the SM genes had a higher degree of LD than the FM genes for all 11 HapMap populations; and (6) the SM genes had a higher degree of LD than the FM genes for all 13 1000 genomes populations. These conclusions supported the hypothesis that the SM genes were more evolutionarily conserved than the FM genes.

Furthermore, we also compared the functional difference between the SM and FM genes using the GO database. The BP and MF categories were considered. The comparison showed that SM genes were mainly annotated to metabolic processes maintaining the basic survival of the cell, while FM genes were mainly annotated to processes or functions interacting with the environment, such as sensory perception of smell and sensory perception of chemical stimulus. A broad definition for metabolism is the sum of the biochemical processes of living organisms [20]. Metabolism performs a fundamental role in biology and impacts almost all functions of cells [21]. Many studies have demonstrated the relationship between metabolism and the pathogenesis of disease [20, 22, 23], implying that the SM genes involved in metabolic processes play crucial roles in the genome. These genes might have stable genomic structures, and undergo few mutations and recombination events during evolution. Compared with the SM genes, the FM genes showed less evolutionary conservation in both long-term evolutionary features and short-term evolutionary features. They were mainly involved in the response to environmental stimuli, implying that the FM genes exhibit less genomic stability and evolve faster during evolution to allow adaptation to changes in the environment.

To test whether the results of our study were strong and reliable, we also extracted top (bottom) 15% and top (bottom) 25% genes (as ranked by FC values) and investigated the difference of evolutionary features for the two extreme gene sets. We found that the results of 15% and 25% are consistent with that of top and bottom 20%. The results for the top (bottom) 15% and top (bottom) 25% of genes can be found in the Supplementary File Section 4 for 15% (from Supplementary Figure 4-1 to Supplementary Figure 4-10 and from Supplementary Table 4-1 to Supplementary Table 4-4), and Section 5 for 25% (from Supplementary Figure 5-1 to Supplementary Figure 5-10 and from Supplementary Table 5-1 to Supplementary Table 5-4).

In this study, considering the nonuniform of methylation levels along the gene region, we re-analyzed the relationship between the fluctuation of methylation levels and evolutionary conservation on 5 different DNA elements including exon, intron, 3′UTR, 5′UTR and upstream regions of the transcription start site (TSS1500). For each region, we found that the results still supported that the SM genes had higher evolutionary conservation than FM genes. The results for 5 different regions can be found in the Supplementary File Section 6 for exon (from Supplementary Figure 6-1 to Supplementary Figure 6-10 and from Supplementary Table 6-1 to Supplementary Table 6-4), Section 7 for intron (from Supplementary Figure 7-1 to Supplementary Figure 7-10 and from Supplementary Table 7-1 to Supplementary Table 7-4), Section 8 for 3′UTR (from Supplementary Figure 8-1 to Supplementary Figure 8-10 and from Supplementary Table 8-1 to Supplementary Table 8-4), Section 9 for 5′UTR (from Supplementary Figure 9-1 to Supplementary Figure 9-10 and from Supplementary Table 9-1 to Supplementary Table 9-4) and Section 10 for TSS1500 (from Supplementary Figure 10-1 to Supplementary Figure 10-10 and from Supplementary Table 10-1 to Supplementary Table 10-4). These results indicated that the nonuniform of methylation levels along the gene region had little effect on the analysis result.

As mentioned above, SM genes were defined as genes that have stable DNA methylation levels in all cell types of every tissue under normal or disease conditions. Therefore, all 8676 samples from the 108 series of GPL13534 were put together to form a large data set and used to compare the evolutionary conservation between SM and FM genes. To illustrate whether the disease condition could influence the results, we used a methylation dataset (GSE40699) which included 60 different normal cell lines from ENCODE Project and investigated the relationship between the stability of methylation status and evolutionary conservation. The results still supported the hypothesis that the SM genes had higher evolutionary conservation than FM genes (for details, see Supplementary File Section 11, from Supplementary Figure 11-1 to Supplementary Figure 11-10 and from Supplementary Table 11-1 to Supplementary Table 11-4). The consistency of the results suggested that the disease information did not affect the results. These tests indicated that our results are highly reliable.

By comparing of the evolutionary features between the SM genes and the FM genes, we got results and conclusions supporting that genes with stable DNA methylation levels show higher evolutionary conservation than genes with fluctuant DNA methylation levels. These results and conclusions may benefit for the following researches associated with the stability of methylation levels or evolutionary conservation. Based on these results, we could further explore the relationship between the stability of methylation levels and the stability of other omics such as genomics, transcriptome and proteomics. We also could try to investigate the relationship between the stability of methylation levels and complex diseases. Ultimately, we expect that our results will improve our understanding of the stabilizing mechanisms of biological systems.

## MATERIALS AND METHODS

### DNA methylation data

The DNA methylation data for human genes were obtained from the NCBI GEO database [24, 25]. We downloaded methylated and unmethylated signal data of all the 108 series from the GPL13534 platform (Illumina HumanMethylation450 BeadChip, including 485,577 probes). All the methylated and unmethylated signal values were normalized [26]. The total number of samples was 8,676. For each sample, we used the *β*-value to represent the methylation status at a probed location. The *β*-value was defined as

β=M/(U+M+100) (1),

where *M* is the methylated signal and *U* is the unmethylated signal at a probed location and 100 is a constant for regularizing the beta value when both methylated and unmethylated probe intensities are low [27]. We then mapped the probes to genes based on the annotation information of the platform. The methylation level of a gene, *β*_*g*_, was defined as the average *β*-values of probes mapped to the gene *g*. Considering the possible batch effect among different series, we performed adjustment for *β*_*g*_ in different series using the Empirical Bayes (EB) batch correction method [28]. Ultimately, the DNA methylation data set included 21,231 genes and their DNA methylation levels in 8,676 samples.

### SM genes and FM genes

For a gene *g*, we use the variance of *β*_*g*_ to measure the variations of DNA methylation levels. In this study, the variance of *β*_*g*_ is also called the fluctuation coefficient, FC. The FC of a human gene *g* is defined as:

$$FC_{g}=\mathrm{var}(\beta_{g})=\frac{1}{n-1}\sum_{i=1}^{n}{\left(\beta_{g,i}-{\bar{\beta }}_{g}\right)}^{2}$$ (2)

where *β*_*g,i*_ is the DNA methylation level of gene *g* in the *i* th sample, *n* is the number of samples examined, and *β*_*g*_ is the average DNA methylation level of gene *g* across the *n* samples. A higher *FC*_*g*_ value indicates larger variations in DNA methylation levels across samples, and a lower *FC*_*g*_ value indicates lower variations (or higher stability) in DNA methylation levels across samples. We then sorted the genes from low *FC*_*g*_ values at the top to highest *FC*_*g*_ values at the bottom. The top 20% of sorted genes are used as SM genes, and the bottom 20% of sorted genes are used as FM genes. Finally, we obtained 4,247 SM genes and 4,247 FM genes.

### Orthologous genes

Orthologous genes of the human genes were downloaded from the Ensemble database (ftp://ftp.ensembl.org/pub/release-69/mysql/ensembl_mart_69) [29–31]. There were 21 species (Full names and abbreviations of the 21 species can be seen in Supplementary Table S5) which had non-null data. We extracted the one-to-one orthologous genes [32], and the dn (rate of non-synonymous substitutions) and ds (synonymous substitutions) values from the downloaded files of 21 species. For SM and FM genes, the percentage of orthologous genes for each species was calculated. For each pair of human-other species orthologous genes, we calculated the evolutionary rate dn/ds.

To calculate the sequence identity, we downloaded the protein sequences between pairwise human-other species orthologous genes from BioMart (http://www.ensembl.org/biomart/martview) [33, 34]. The BLASTP program and the BLOSUM62 matrix [35, 36] were used to align the orthologous sequences. The sequence identity was defined as the percent identity of the match.

### SNP data

The SNP data were downloaded from the NCBI SNP database (http://www.ncbi.nlm.nih.gov/SNP/) [37]. The ID numbers (rs#) and positions of the SNPs were extracted. We then downloaded the location information (start and end position) of SM and FM genes from NCBI (ftp://ftp.ncbi.nlm.nih.gov/genomes/MapView/), and mapped the SNPs to the SM and FM genes based on the chromosomal location. Finally, we calculated the SNP density (the number of SNPs divided by the length of the gene) for each SM or FM gene.

### The HapMap project data

We used public genotype data of common SNPs from the HapMap project [38, 39]. The raw data were downloaded from the NCBI HapMap website (ftp://ftp.ncbi.nih.gov/hapmap). 1,117 unrelated individuals from 11 global populations [40] were extracted from the raw data (Full names and abbreviations of the 11 HapMap populations can be seen in Supplementary Table S5). The SNPs included in this study passed the following quality control (QC) criteria: minor allele frequency (MAF) is greater than 0.01, P-value of the Hardy-Weinberg equilibrium (HWE) test is greater than 0.001, call frequency is greater than 0.75, and the SNP must be detected in all 11 populations. We then mapped the SNPs to the SM and FM genes based on the location information, and calculated the pairwise linkage disequilibrium (LD) coefficient, r^2^, for all the SNPs in an SM or FM gene region using the Haploview software [41]. The median pairwise r^2^ in a gene region was calculated and compared between SM and FM genes.

### The 1000 genomes project data

To verify our conclusions, we used another high-quality data set, the 1000 genomes project [42], to compare the degree of LD between SM and FM genes. The raw genotype data were downloaded from the NCBI 1000 genomes website (http://ftp.ncbi.nlm.nih.gov/1000genomes/). 1,063 unrelated individuals from 14 global populations were extracted from the raw dataset (for details, see Supplementary Table S5). Only 13 populations were included in this study, because the IBS population only had 14 samples. The SNP genotype data of the 1000 genomes project were filtered based on the same QC criteria as the HapMap genotype data. Ultimately, the r^2^ for each of the SM and FM genes were calculated.

### Gene ontology (GO) annotation

To better understand the biological reasons for the observed differences in evolutionary conservation, we compared the Gene Ontology (GO) of the SM and FM genes [43–45]. We used the DAVID software [46] to annotate the SM and FM genes. The biological process (BP) and molecular function (MF) were used to compare the functional differences between SM and SM genes. The cell component (CC) annotation results are also listed in Section 2 and 3 of the Supplementary File.

### Statistical analysis

We used the Wilcoxon rank sum test to compare whether an evolutionary feature was significantly different between the SM and FM genes for each individual species or population. We used the Wilcoxon signed rank test [47] to test whether the median of an evolutionary feature was significantly different between the SM and FM genes across all species or populations. All data were processed using Perl scripts (http://www.activestate.com/activeperl). All statistical graphics and calculations were completed using R scripts (http://cran.r-project.org). All the Perl scripts and R scripts can be found at the website: http://www.bioapp.org/research/SMvsFM.

## SUPPLEMENTARY DATA TABLES AND FIGURES


