# Supplementary material for: Genes with stable DNA methylation levels show higher evolutionary conservation than genes with fluctuant DNA methylation levels
Source: Oncotarget. 2015 Oct 19;6(37):40235–46. doi: 10.18632/oncotarget.5504 (PMC4741891; doi:10.18632/oncotarget.5504)
Supplement: Supplementary file 1 [file oncotarget-06-40235-s001.pdf]

SUPPLEMENTARY DATA

SECTION 1: SUPPLEMENTARY TABLES OF THE MANUSCRIPT

Supplementary Table S1: Summary statistics for the comparisons of dn/ds in species

Supplementary Table S2: Summary statistics for the comparisons of sequence identity in species

Supplementary Table S3: Summary statistics for the comparisons of r2 in HapMap population

Supplementary Table S4: Summary statistics for the comparisons of r2 in 1000 genomes populations

Supplementary Table S5: Full names and abbreviations

SECTION 2: THE DETAILED GO ANNOTATION RESULTS OF THE HUMAN SM GENES

Supplementary Table S2-1: BP results of the human SM genes

Supplementary Table S2-2: MF results of the human SM genes

Supplementary Table S2-3: CC results of the human FM genes

SECTION 3: THE DETAILED GO ANNOTATION RESULTS OF THE HUMAN FM GENES

Supplementary Table S3-1: BP results of the human FM genes

Supplementary Table S3-2: MF results of the human FM genes

Supplementary Table S3-3: CC results of the human SM genes

SECTION 4: COMPARISON OF EVOLUTIONARY CONSERVATION BETWEEN SM (TOP 15%) AND FM GENES (BOTTOM 15%)

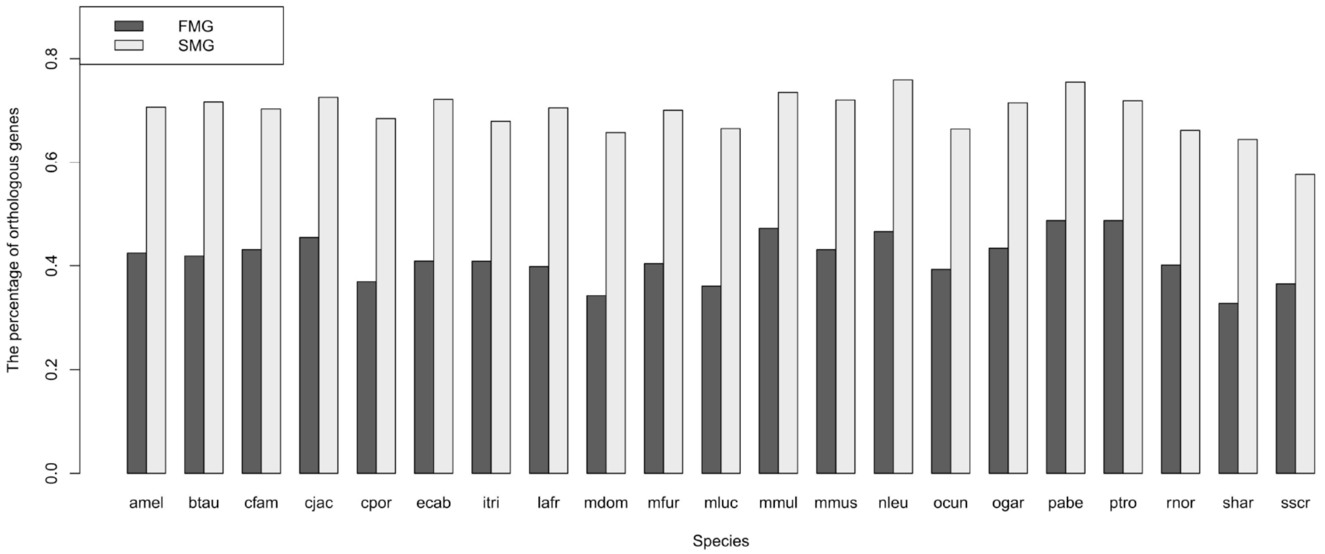

Supplementary Figure S4-1: The bar plots of the SM genes against the FM genes in the percentage of orthologous genes for each of the 21 species.

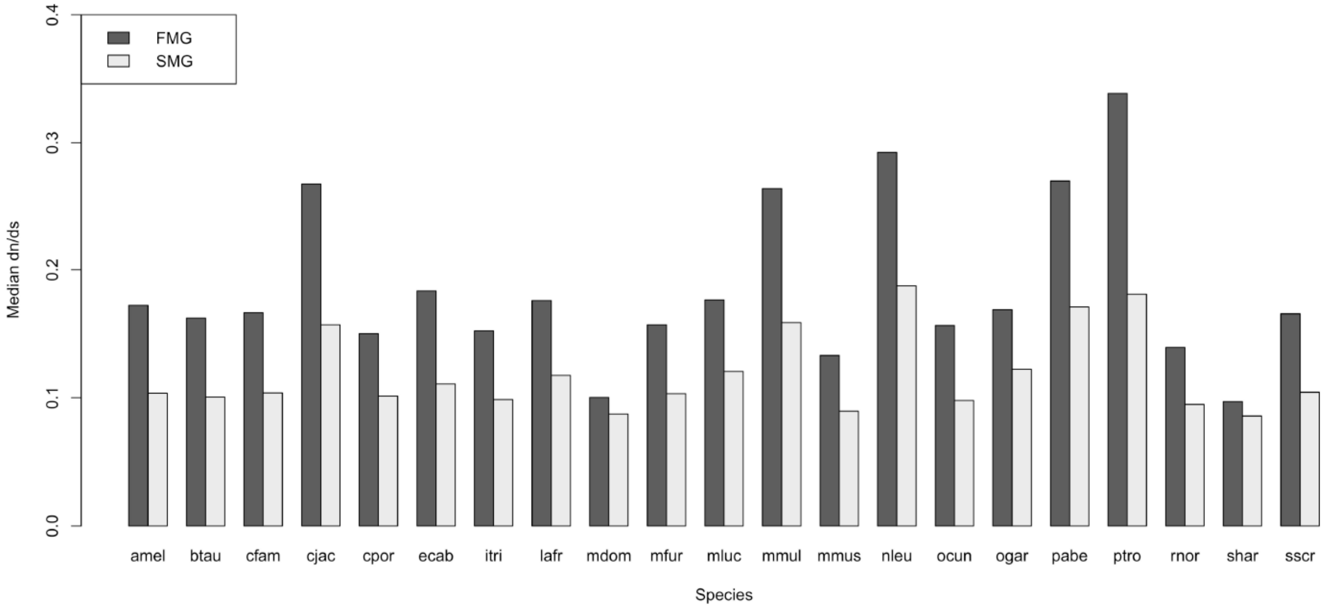

Supplementary Figure S4-2: The bar plots of the SM genes against the FM genes in the evolutionary rate for each of the 21 species.

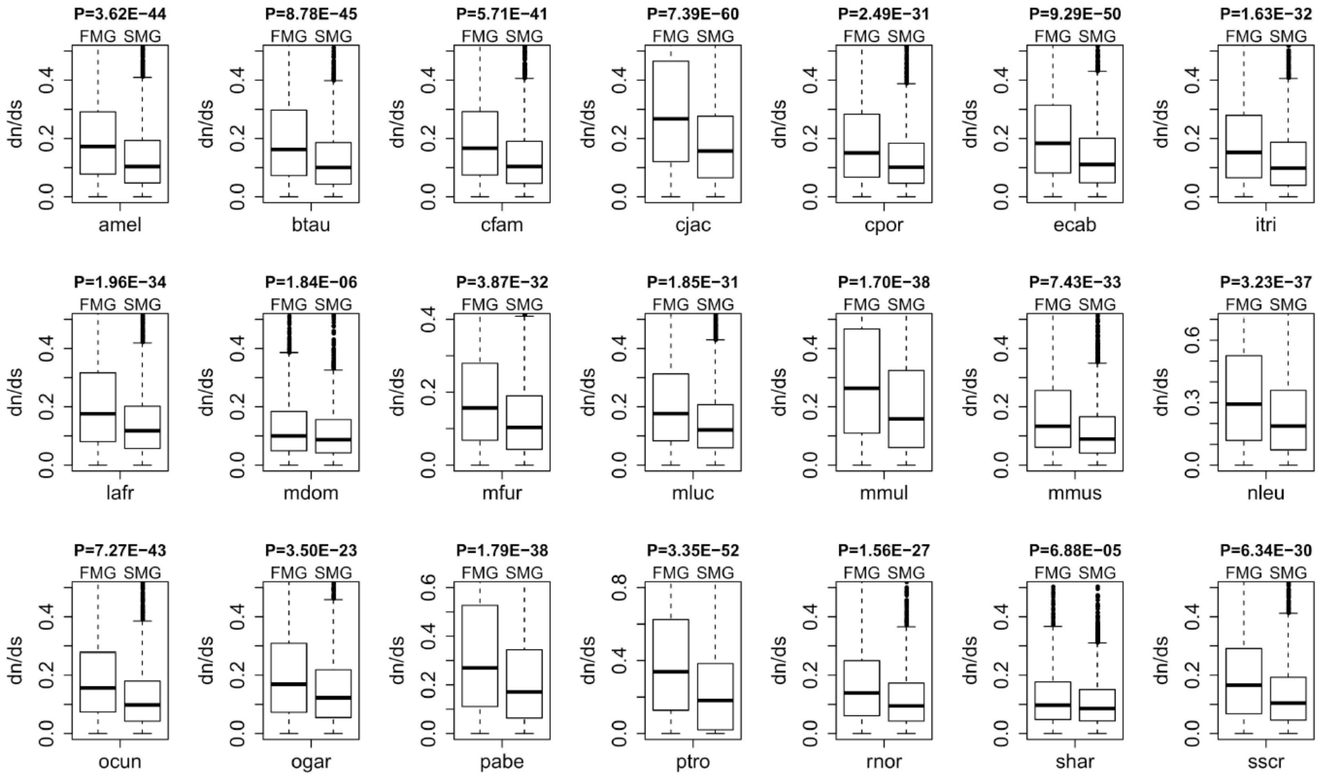

Supplementary Figure S4-3: The box plots of the SM genes against the FM genes in the evolutionary rate for each of the 21 species.

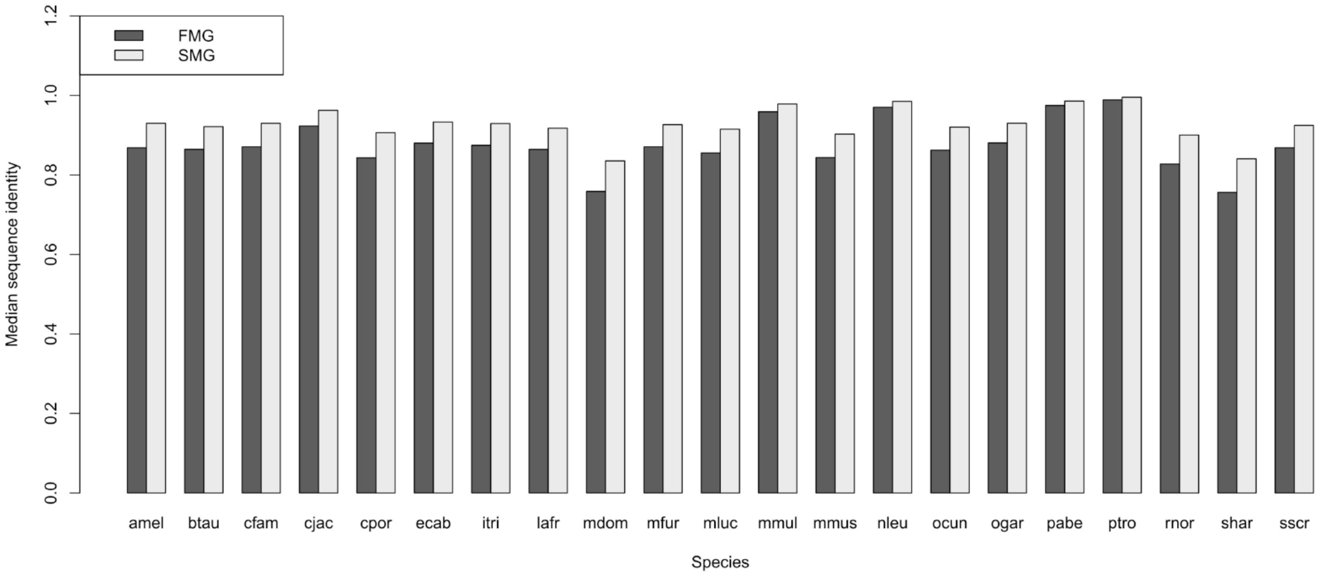

Supplementary Figure S4-4: The bar plots of the SM genes against the FM genes in the sequence identity for each of the 21 species.

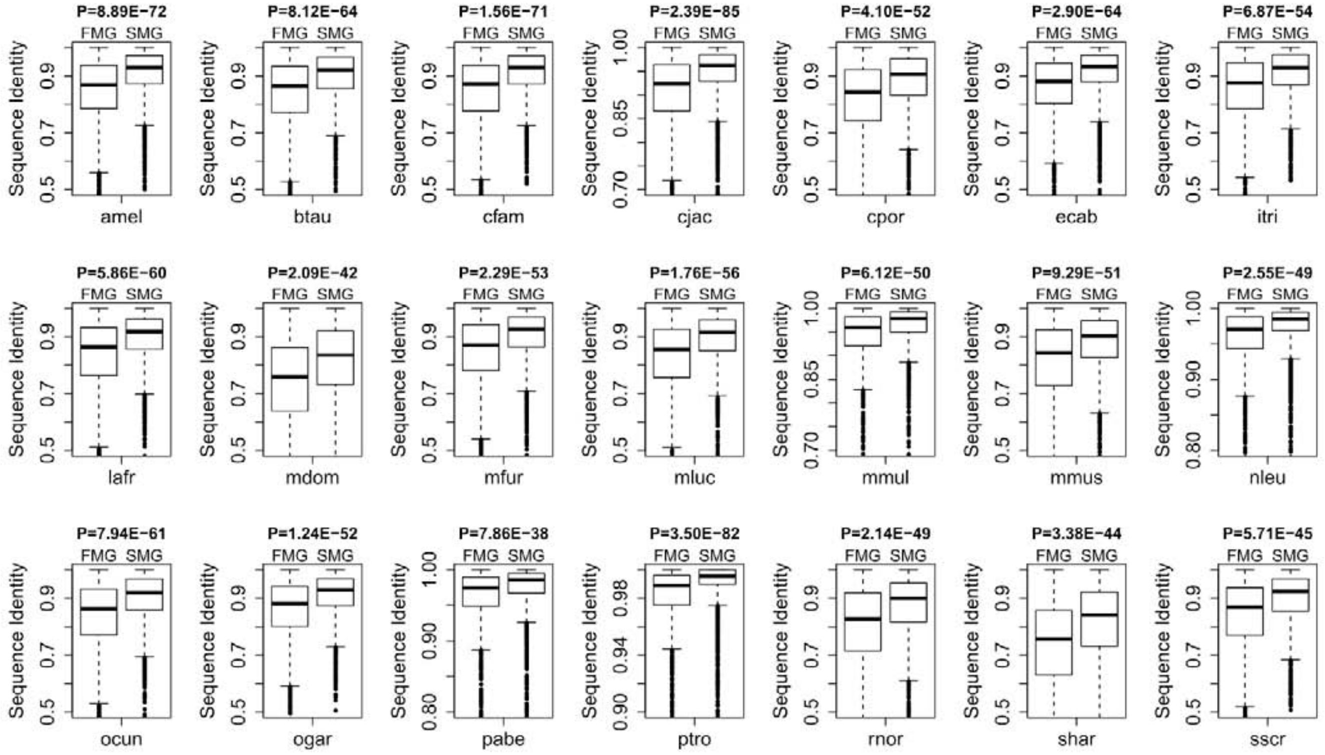

Supplementary Figure S4-5: The box plots of the SM genes against the FM genes in the sequence identity for each of the 21 species.

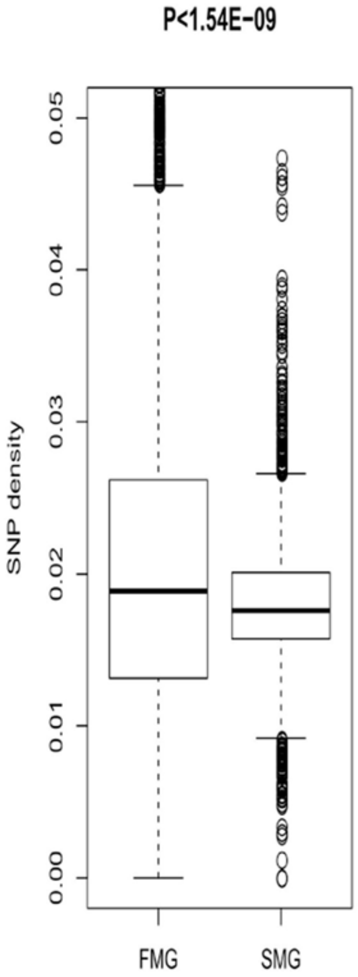

Supplementary Figure S4-6: The box plot of the SM genes against the FM genes in the SNP density.

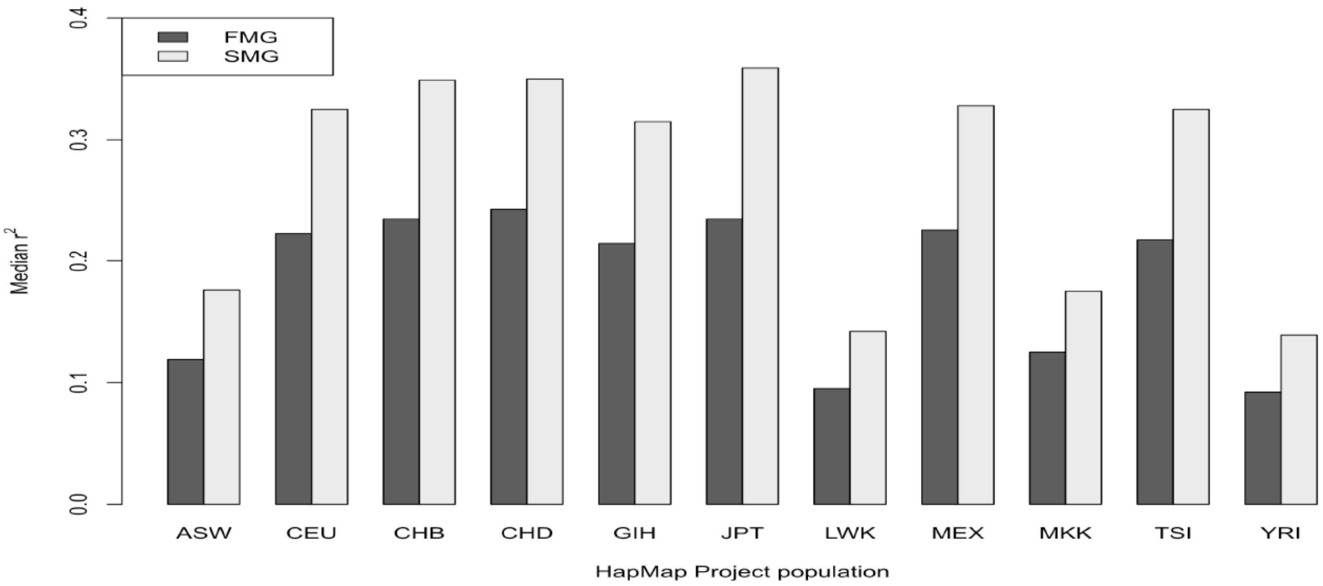

Supplementary Figure S4-7: The bar plots of the SM genes against the FM genes in the LD coefficient r2 for each of the 11 populations of HapMap Project.

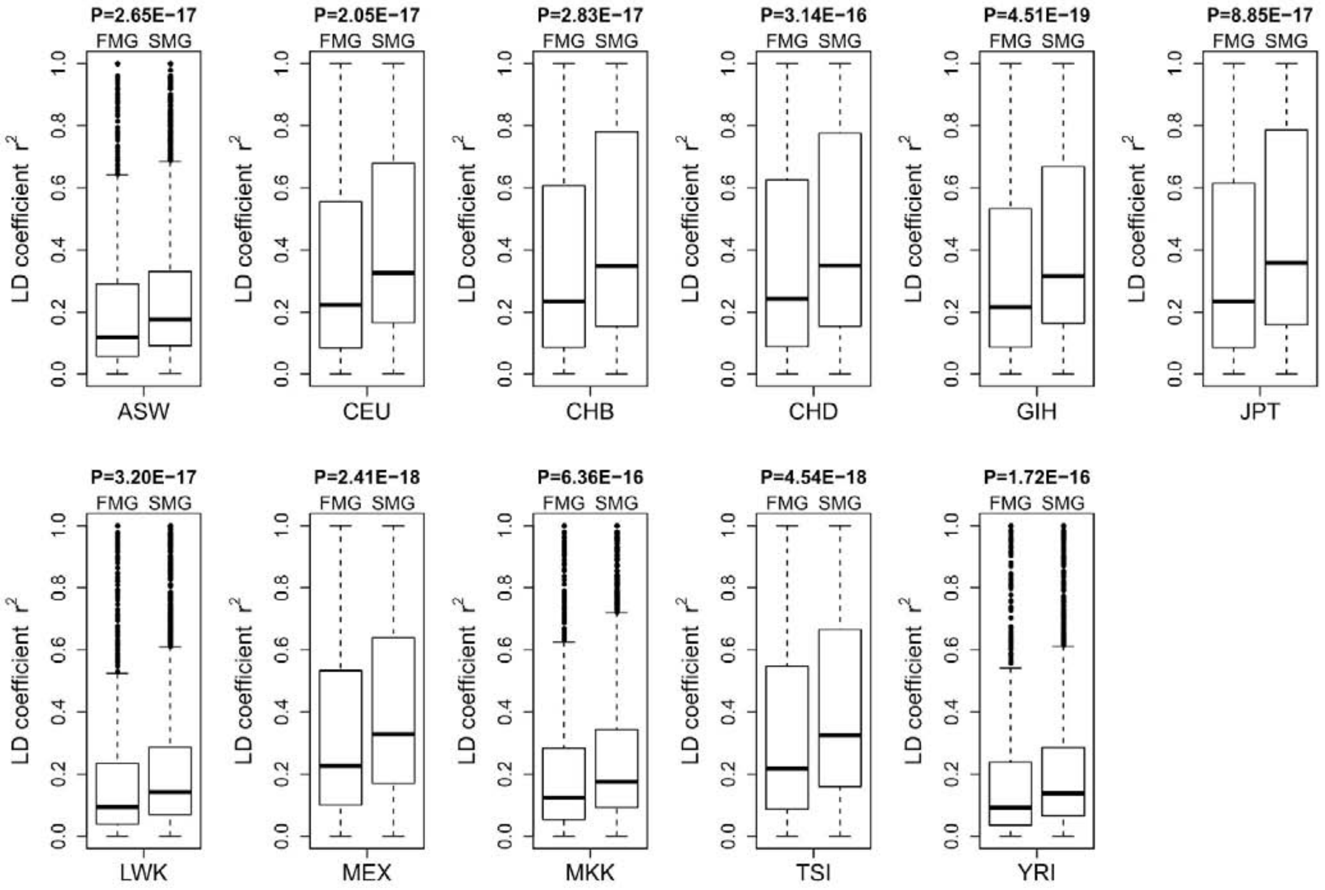

Supplementary Figure S4-8: The box plots of the SM genes against the FM genes in the LD coefficient r2 for each of the 11 populations of HapMap Project.

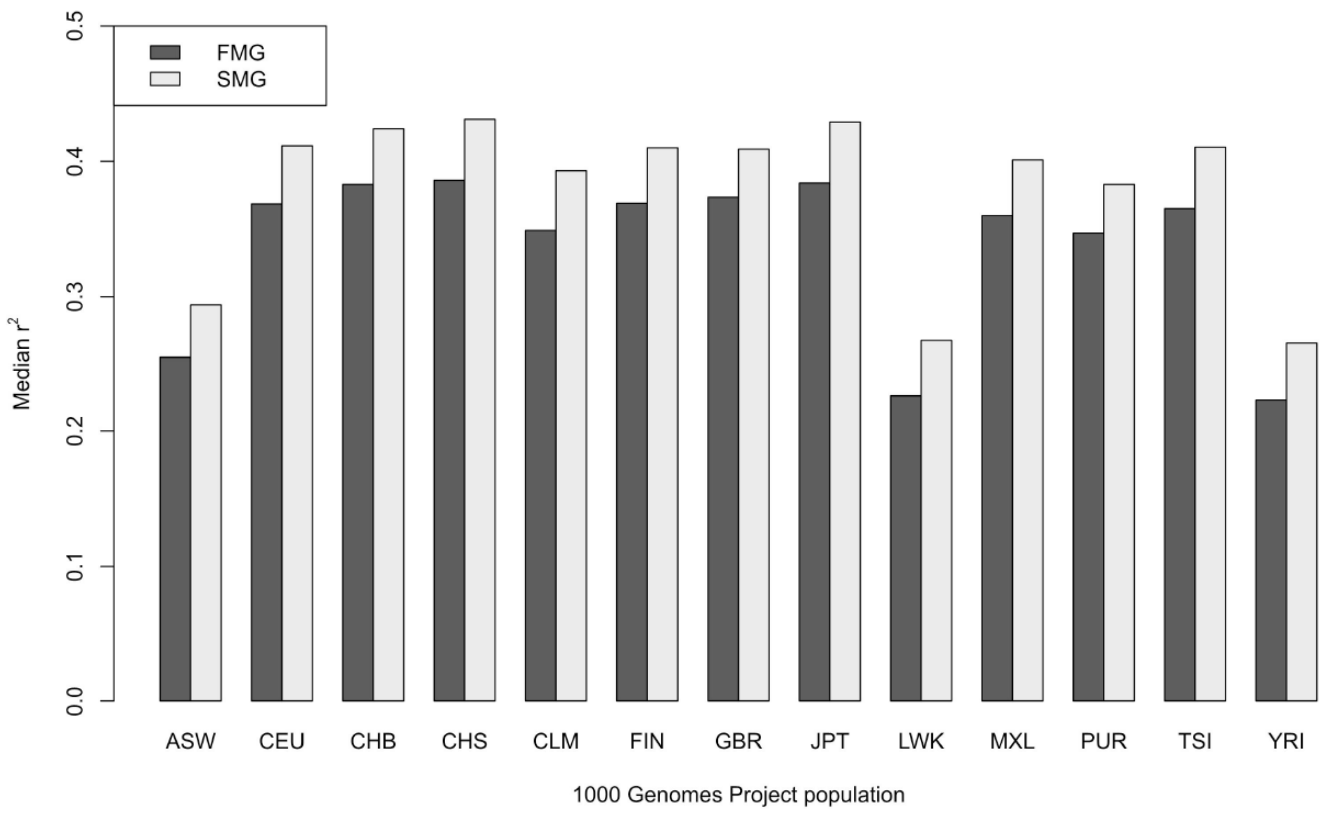

Supplementary Figure S4-9: The bar plots of the SM genes against the FM genes in the LD coefficient r2 for each of the 13 populations of 1000 Genomes Project.

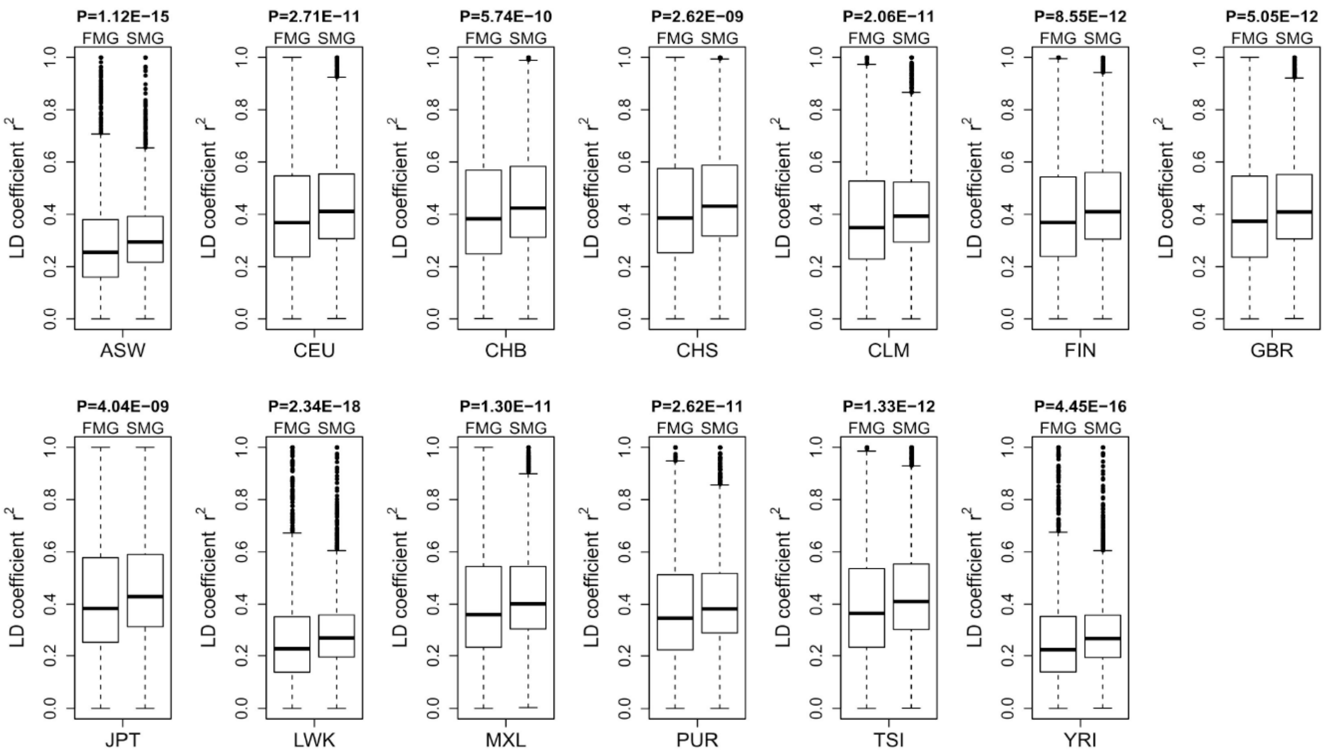

Supplementary Figure S4-10: The box plots of the SM genes against the FM genes in the LD coefficient r2 for each of the 13 populations of 1000 Genomes Project.

Supplementary Table S4-1: Summary statistics for the comparisons of dn/ds in 21 species

Supplementary Table S4-2: Summary statistics for the comparisons of sequence identity in 21 species

Supplementary Table S4-3: Summary statistics for the comparisons of r^2 in HapMap population.

Supplementary Table S4-4: Summary statistics for the comparisons of r^2 in 1000 genomes populations

SECTION 5: COMPARISON OF EVOLUTIONARY CONSERVATION BETWEEN SM (TOP 25%) AND FM GENES (BOTTOM 25%)

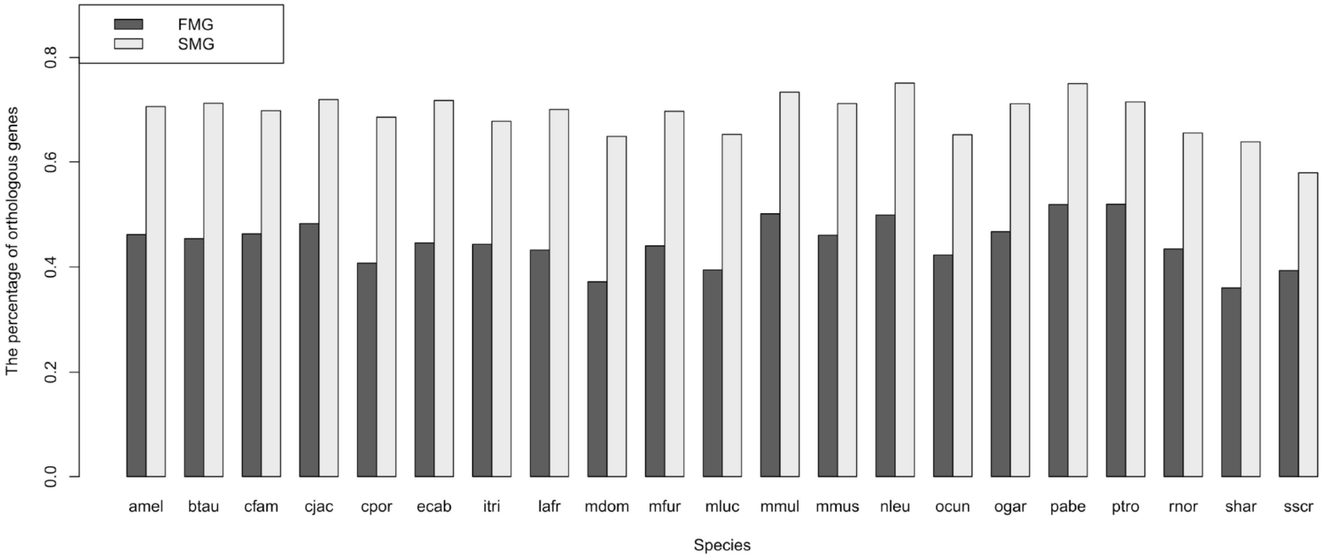

Supplementary Figure S5-1: The bar plots of the SM genes against the FM genes in the percentage of orthologous genes for each of the 21 species.

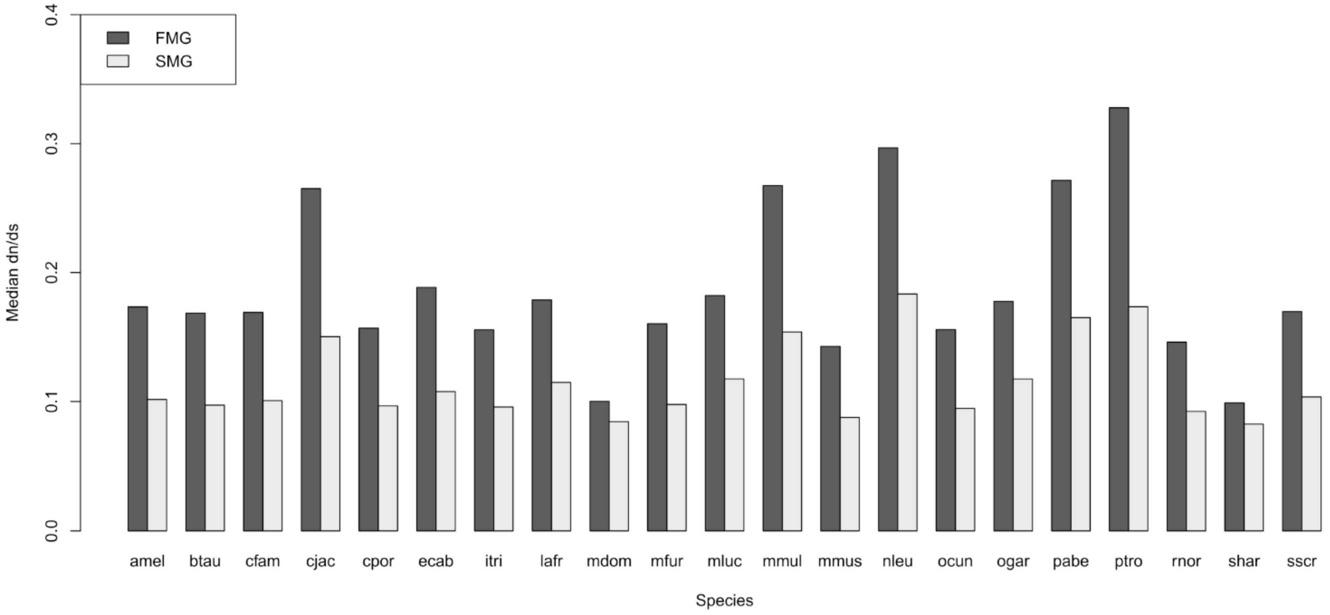

Supplementary Figure S5-2: The bar plots of the SM genes against the FM genes in the evolutionary rate for each of the 21 species.

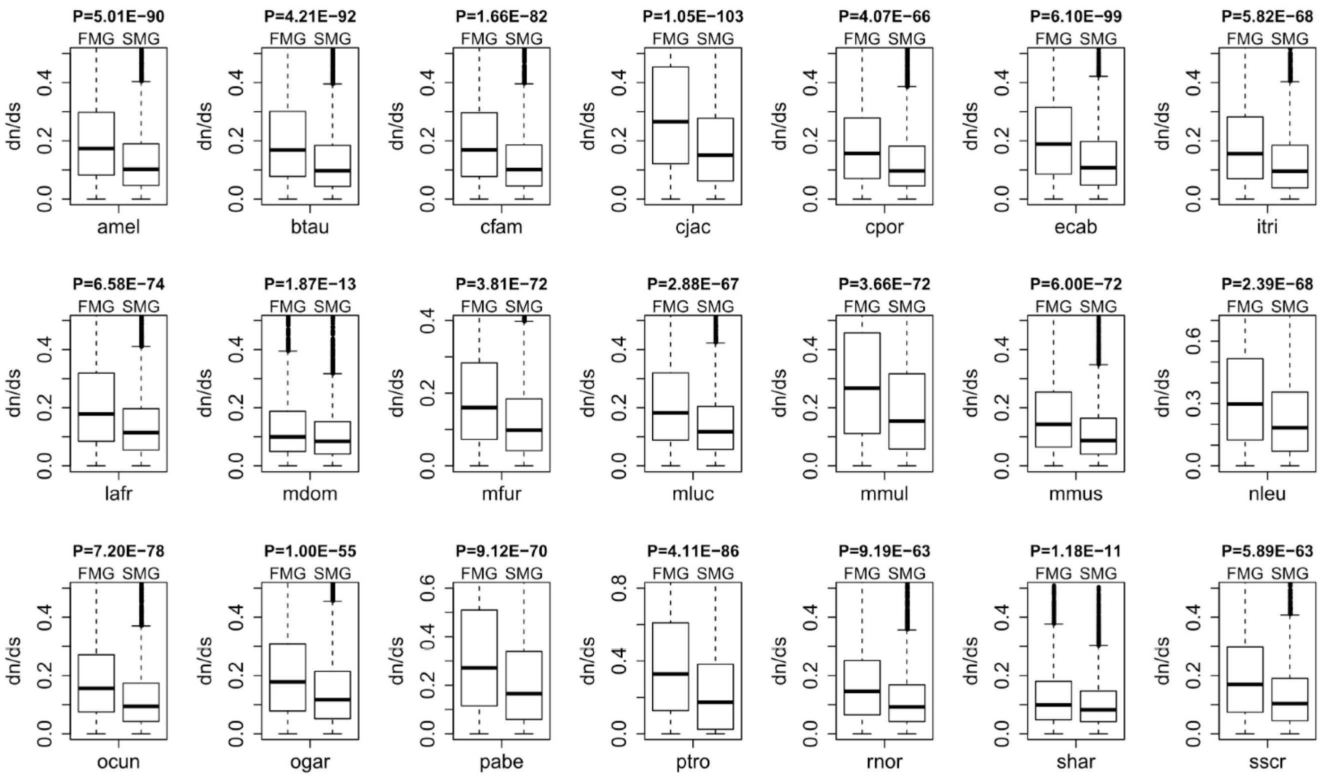

Supplementary Figure S5-3: The box plots of the SM genes against the FM genes in the evolutionary rate for each of the 21 species.

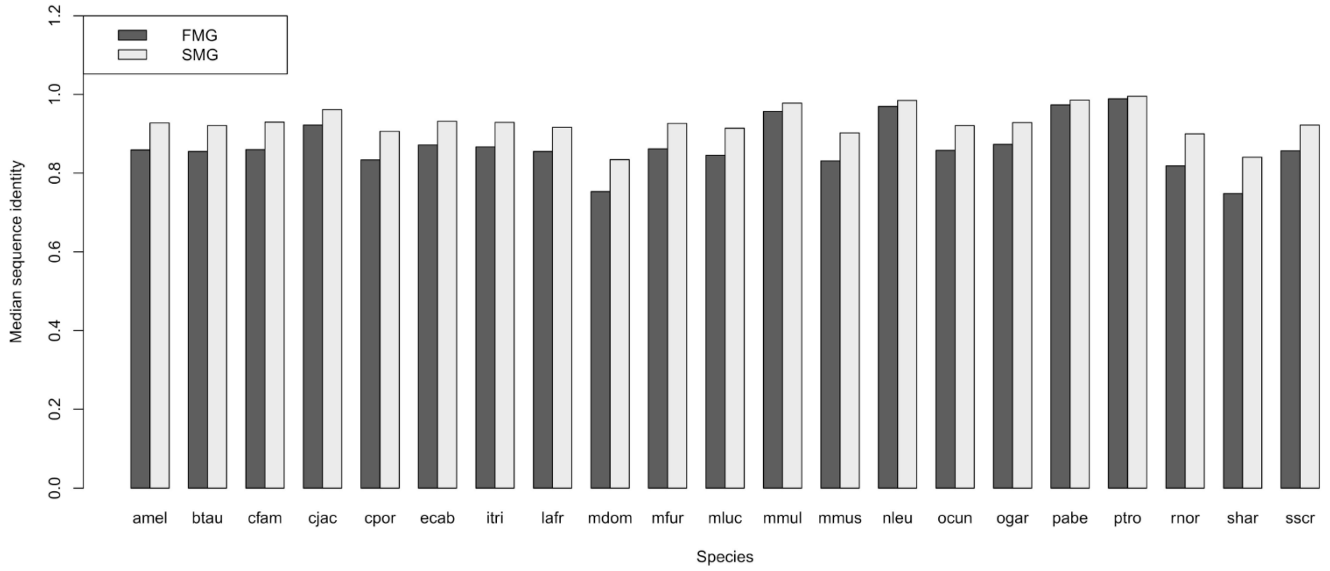

Supplementary Figure S5-4: The bar plots of the SM genes against the FM genes in the sequence identity for each of the 21 species.

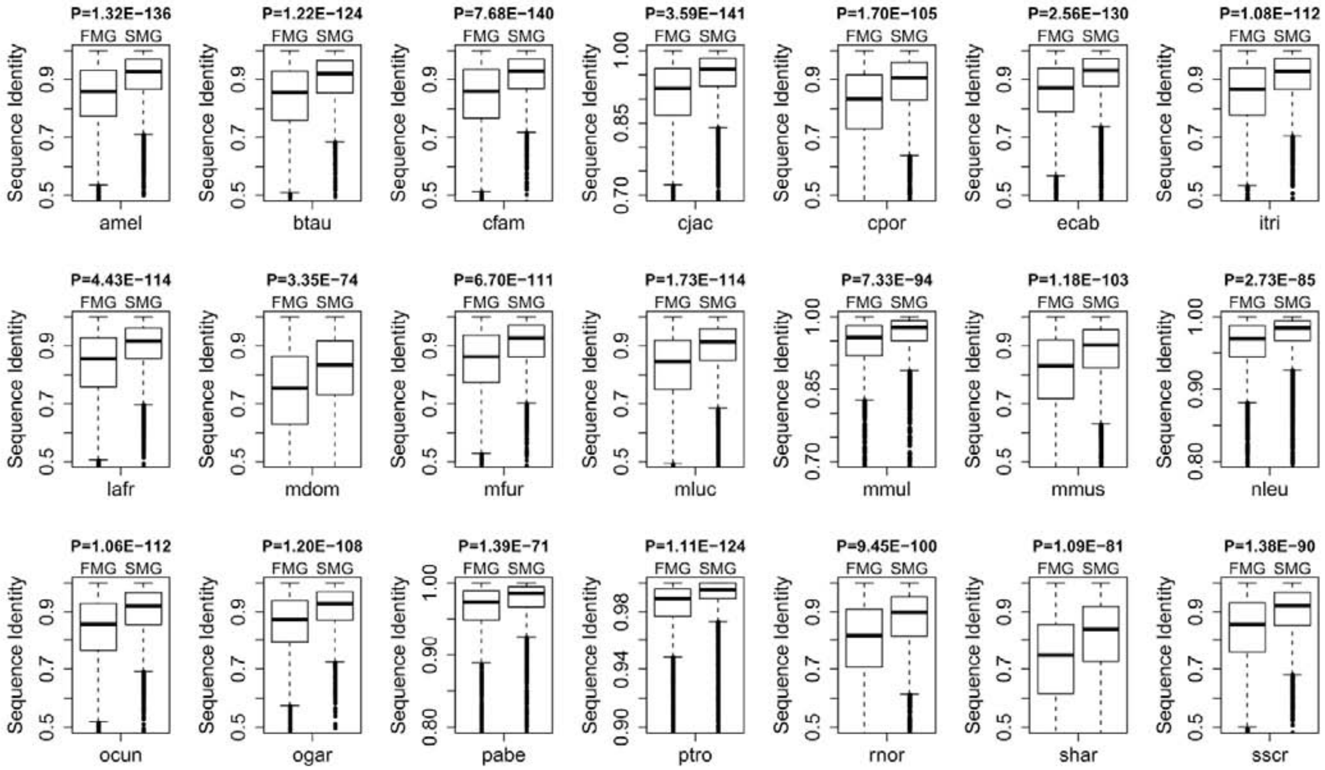

Supplementary Figure S5-5: The box plots of the SM genes against the FM genes in the sequence identity for each of the 21 species.

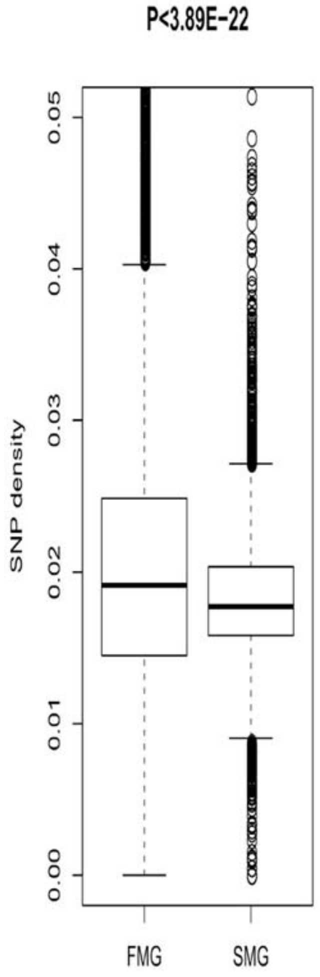

Supplementary Figure S5-6: The box plot of the SM genes against the FM genes in the SNP density.

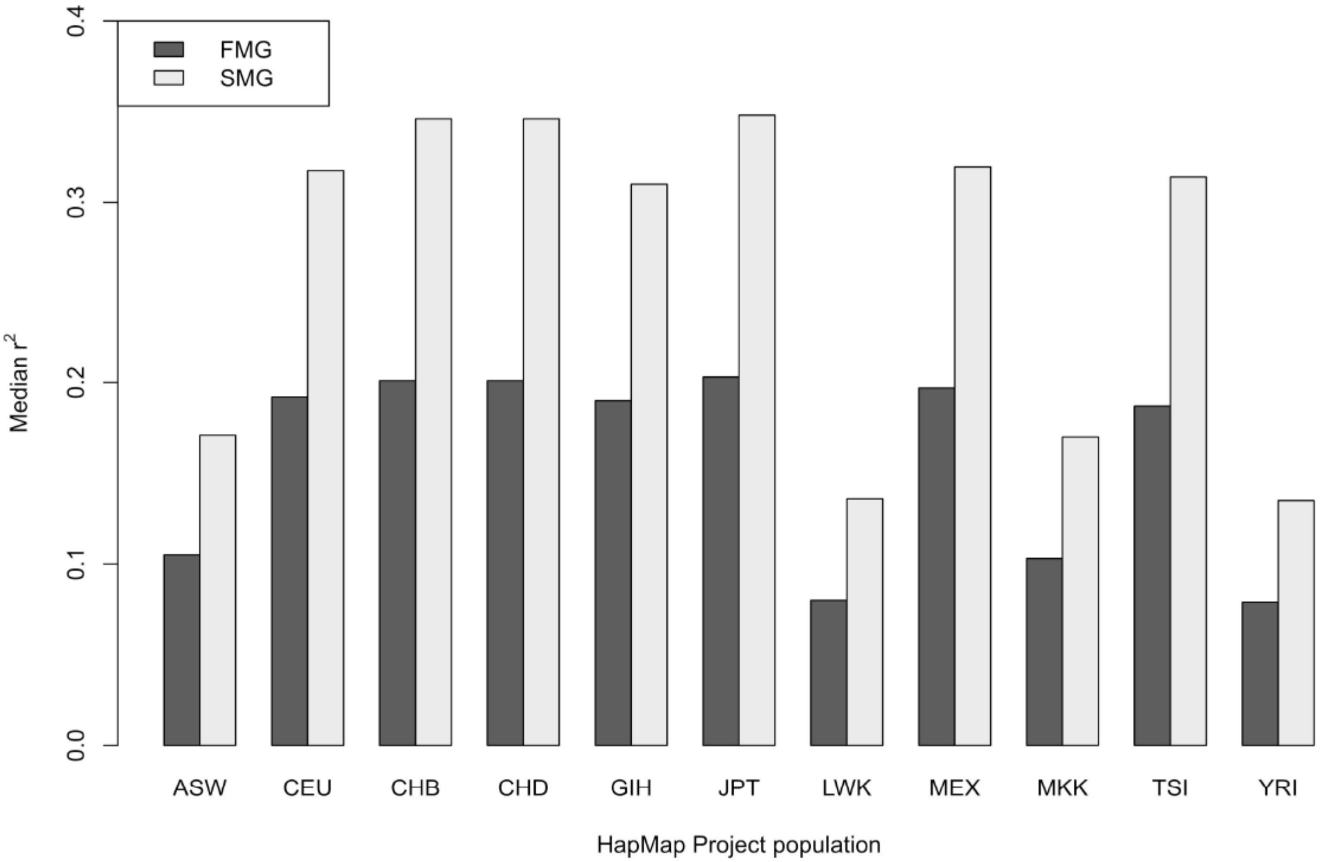

Supplementary Figure S5-7: The bar plots of the SM genes against the FM genes in the LD coefficient r2 for each of the 11 populations of HapMap Project.

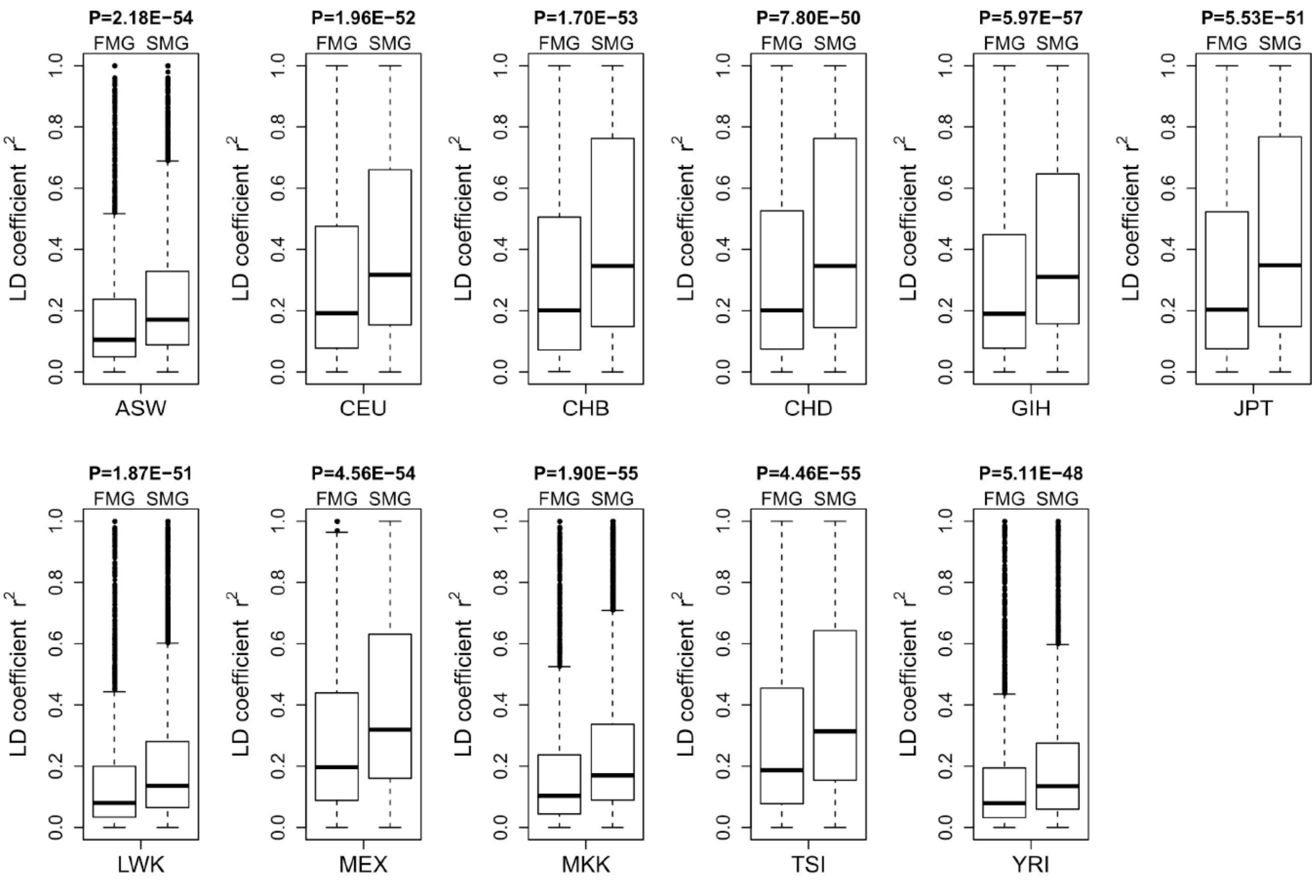

Supplementary Figure S5-8: The box plots of the SM genes against the FM genes in the LD coefficient r2 for each of the 11 populations of HapMap Project.

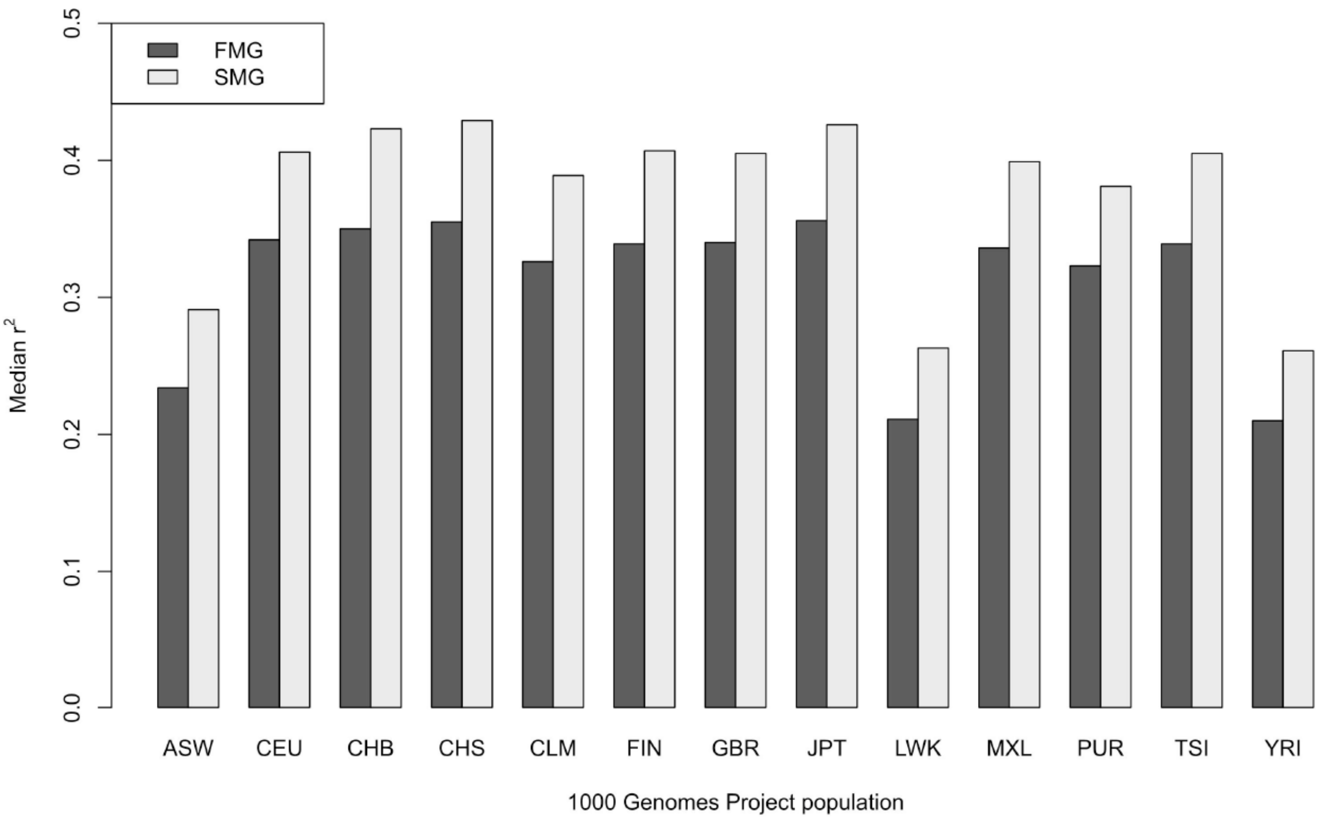

Supplementary Figure S5-9: The bar plots of the SM genes against the FM genes in the LD coefficient r2 for each of the 13 populations of 1000 Genomes Project.

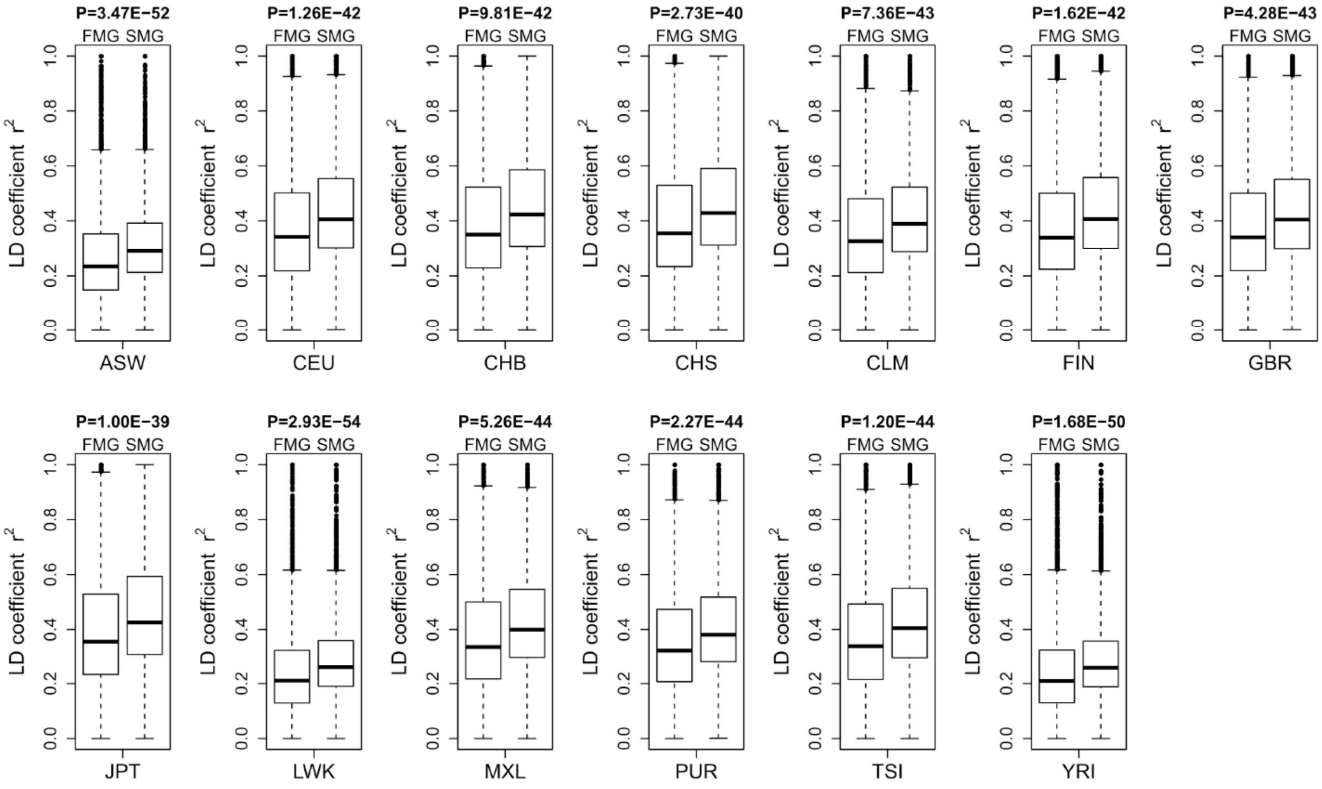

Supplementary Figure S5-10: The box plots of the SM genes against the FM genes in the LD coefficient r2 for each of the 13 populations of 1000 Genomes Project.

Supplementary Table S5-1: Summary statistics for the comparisons of dn/ds in 21 species

Supplementary Table S5-2: Summary statistics for the comparisons of sequence identity in 21 species

Supplementary Table S5-3: Summary statistics for the comparisons of r^2 in HapMap population

Supplementary Table S5-4: Summary statistics for the comparisons of r^2 in 1000 genomes populations

SECTION 6: COMPARISON OF EVOLUTIONARY CONSERVATION BETWEEN SM (TOP 20%) AND FM GENES (BOTTOM 20%) USING EXON DATA

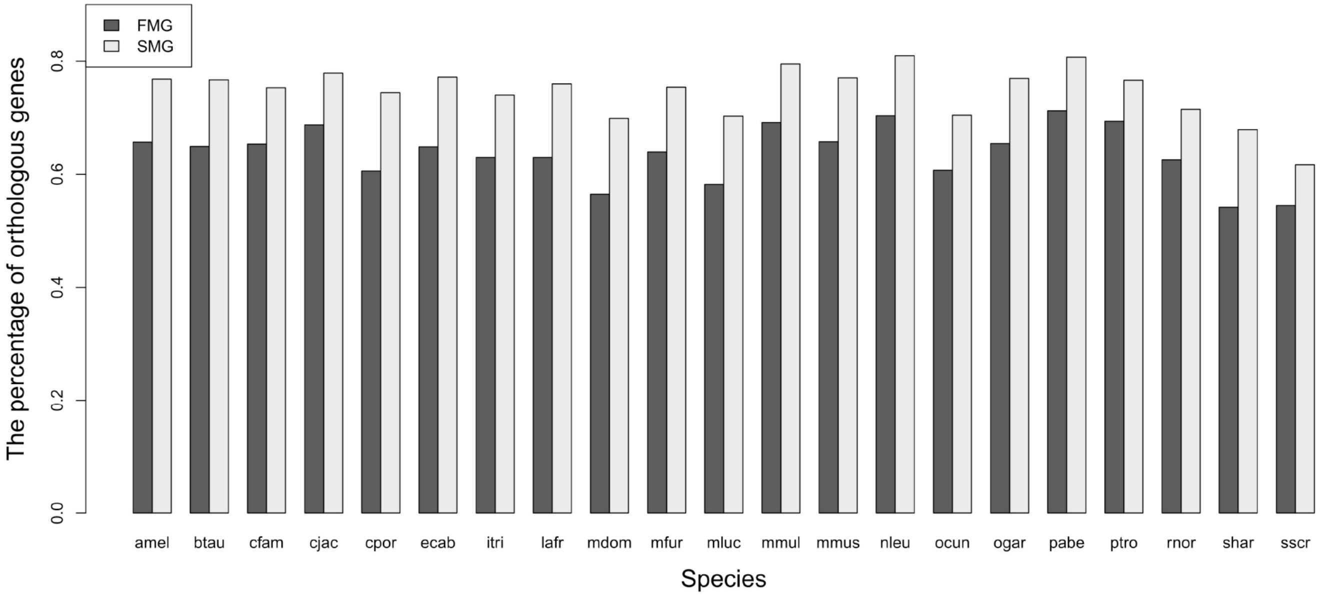

Supplementary Figure S6-1: The bar plots of the SM genes against the FM genes in the percentage of orthologous genes for each of the 21 species.

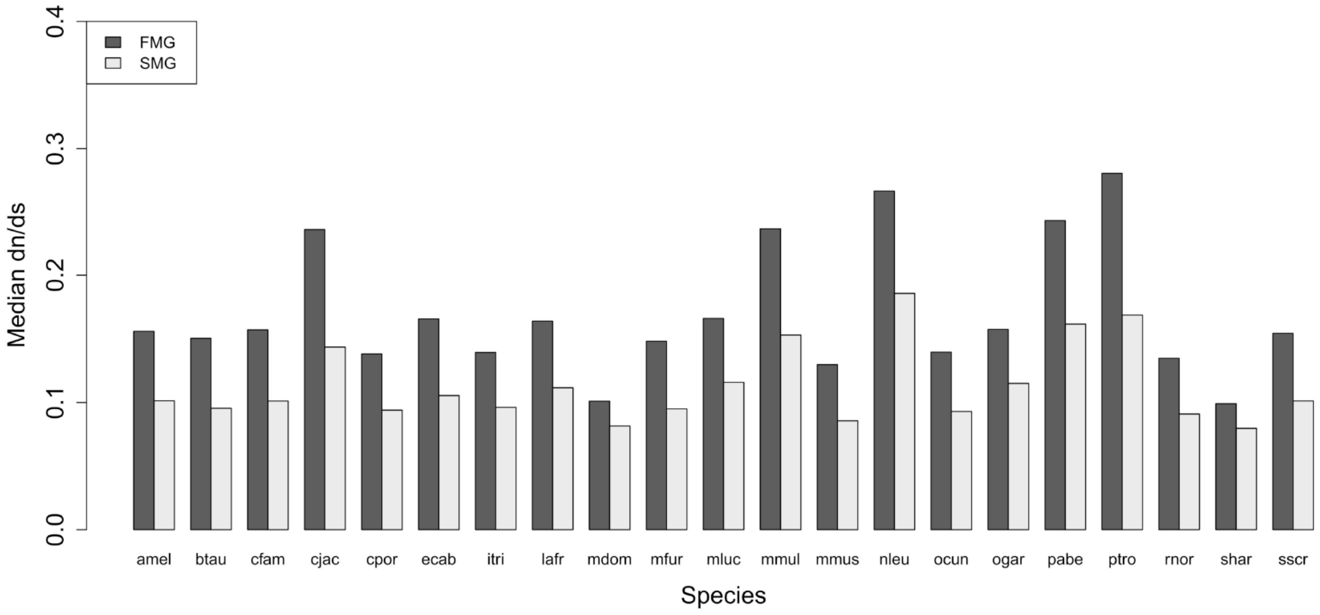

Supplementary Figure S6-2: The bar plots of the SM genes against the FM genes in the evolutionary rate for each of the 21 species.

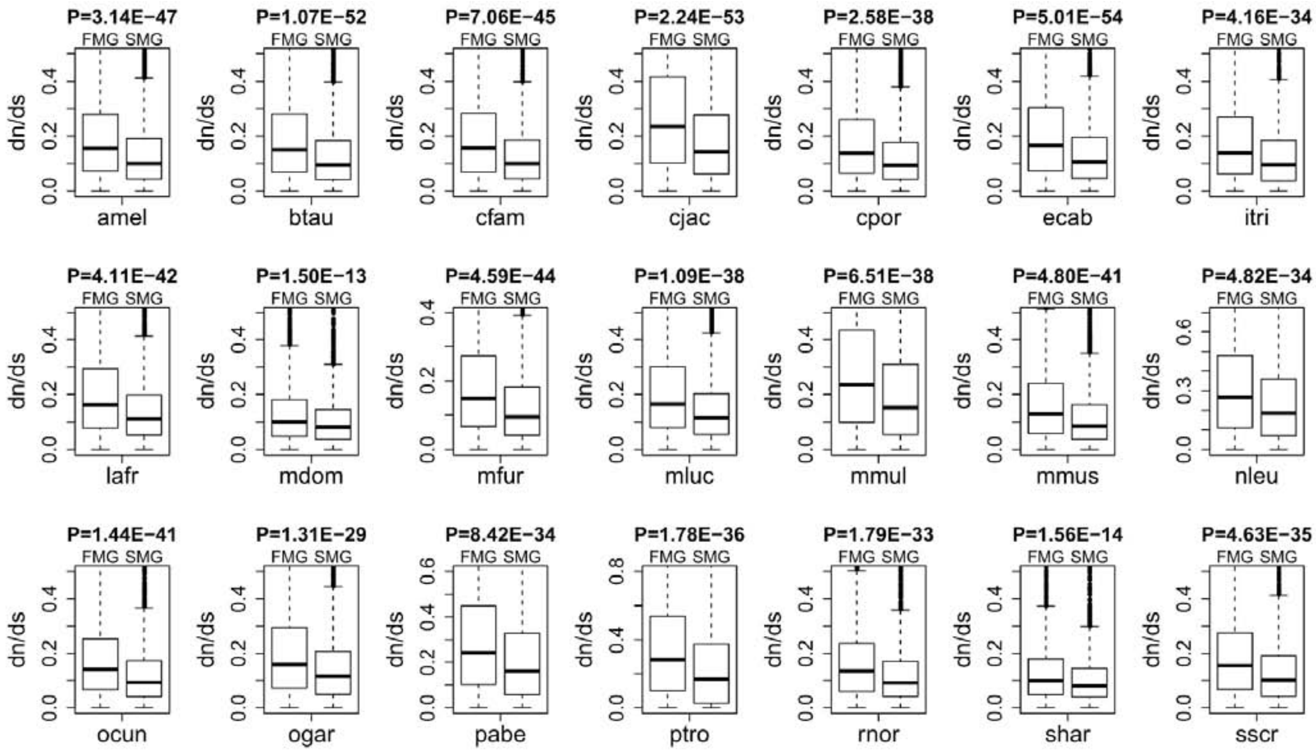

Supplementary Figure S6-3: The box plots of the SM genes against the FM genes in the evolutionary rate for each of the 21 species.

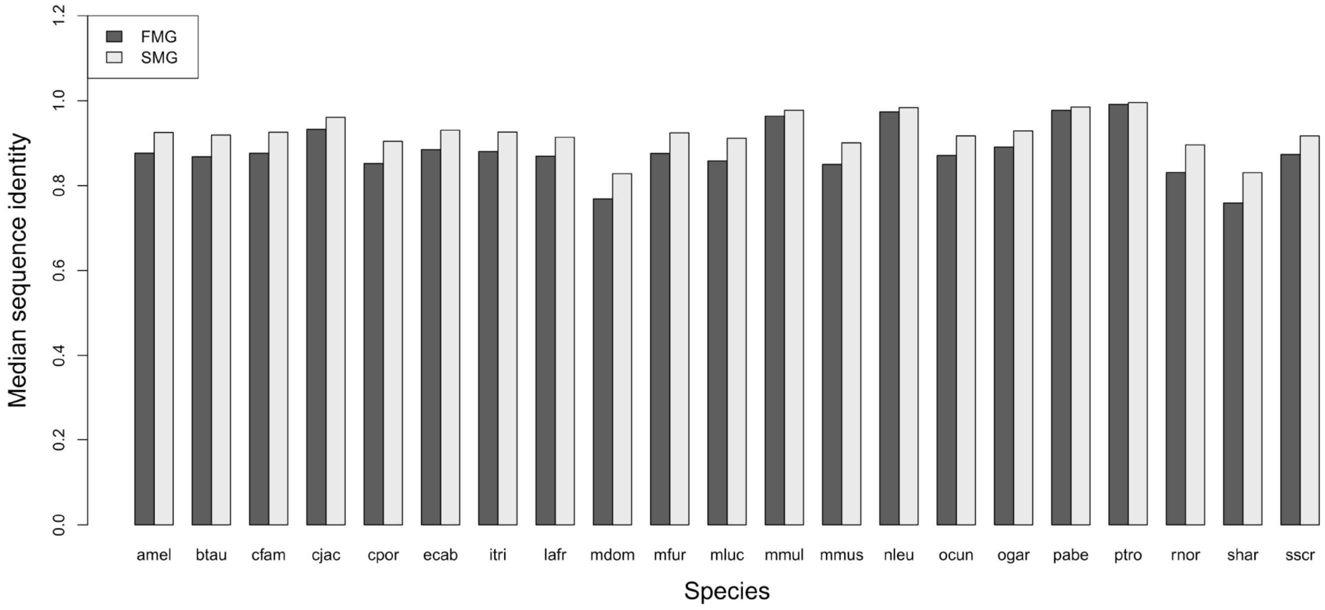

Supplementary Figure S6-4: The bar plots of the SM genes against the FM genes in the sequence identity for each of the 21 species.

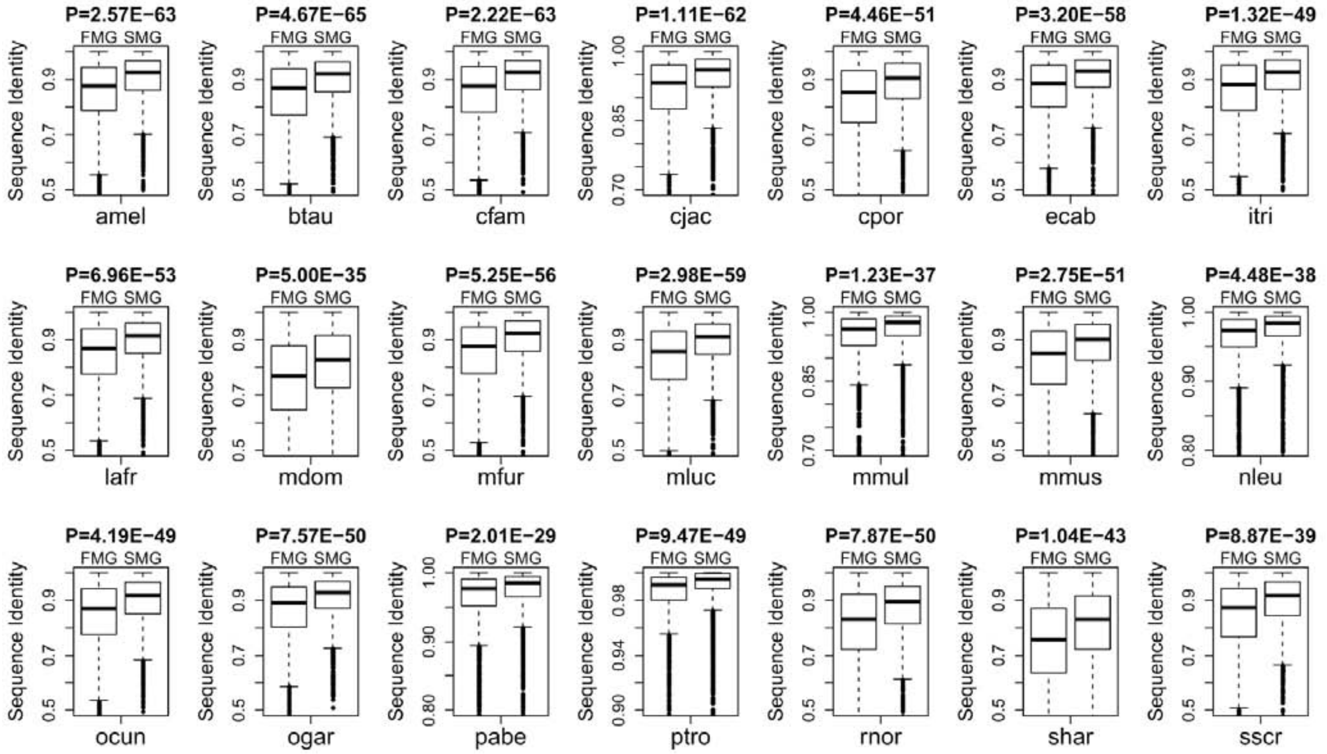

Supplementary Figure S6-5: The box plots of the SM genes against the FM genes in the sequence identity for each of the 21 species.

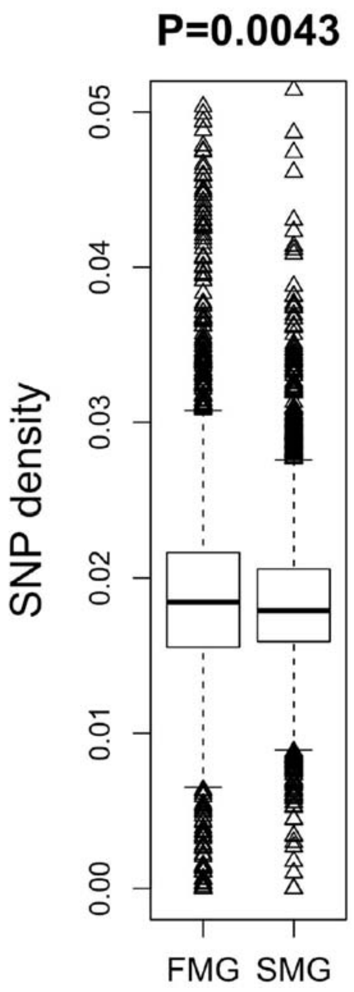

Supplementary Figure S6-6: The box plot of the SM genes against the FM genes in the SNP density.

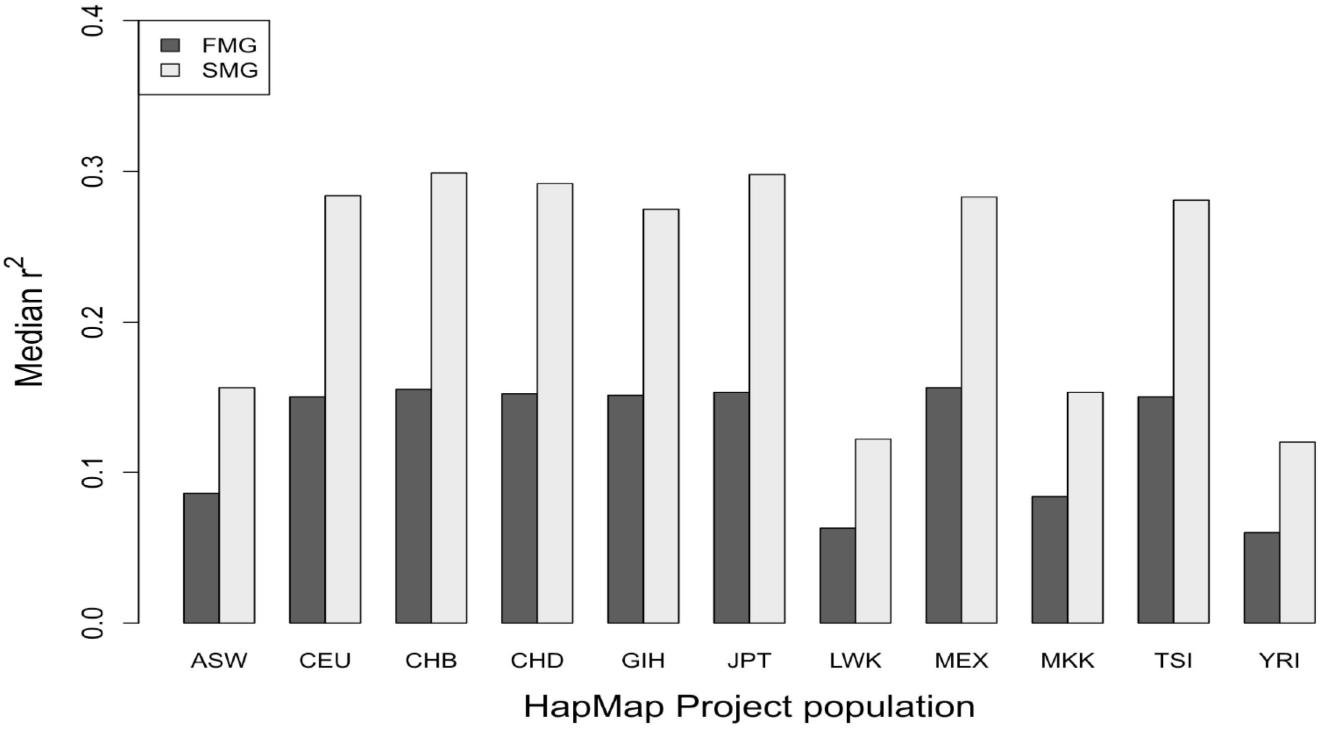

Supplementary Figure S6-7: The bar plots of the SM genes against the FM genes in the LD coefficient r2 for each of the 11 populations of HapMap Project.

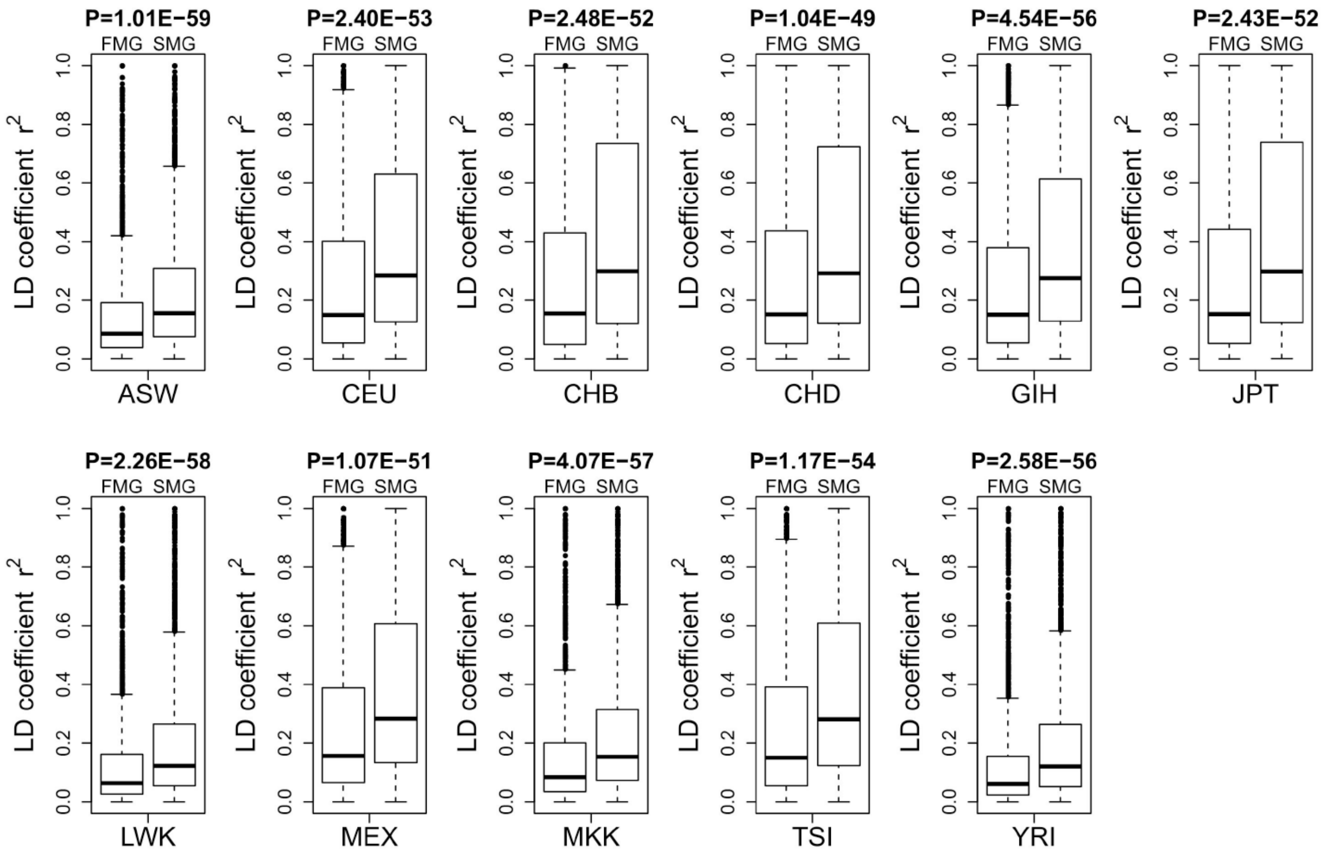

Supplementary Figure S6-8: The box plots of the SM genes against the FM genes in the LD coefficient r2 for each of the 11 populations of HapMap Project.

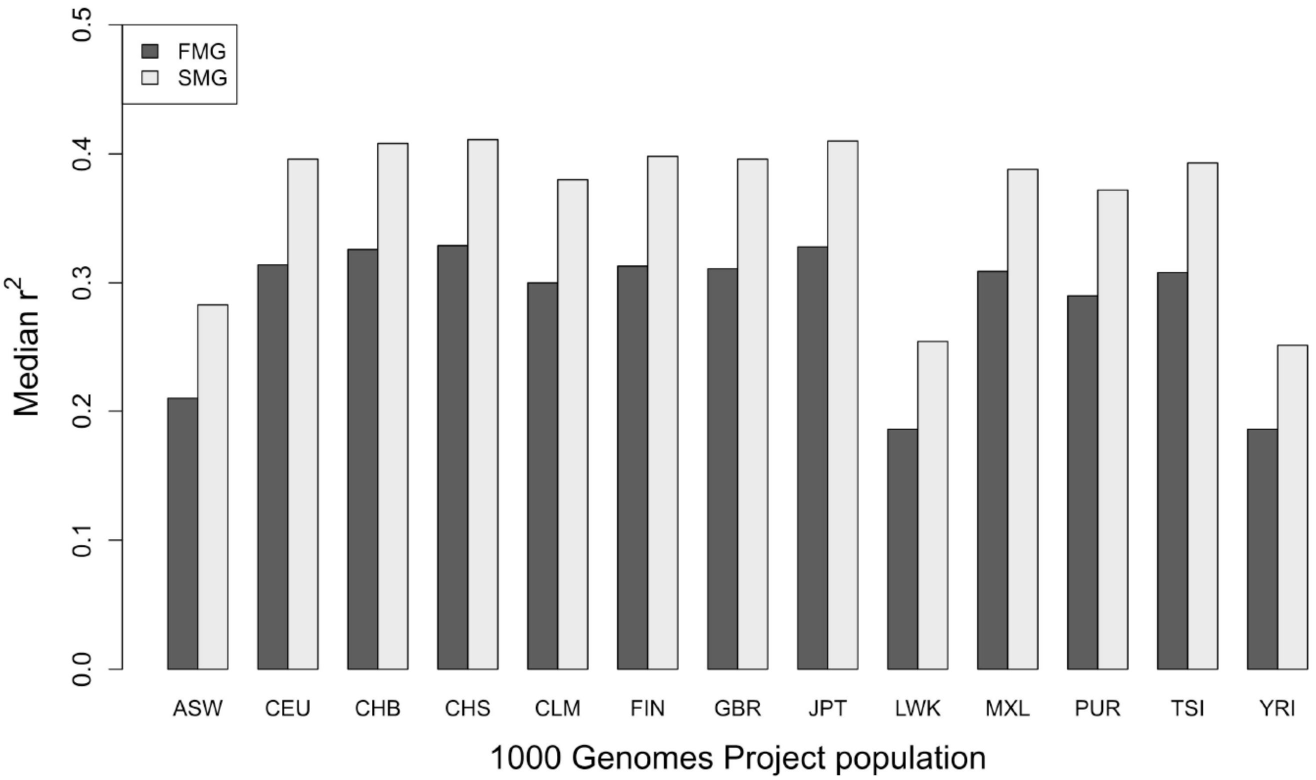

Supplementary Figure S6-9: The bar plots of the SM genes against the FM genes in the LD coefficient r2 for each of the 13 populations of 1000 Genomes Project.

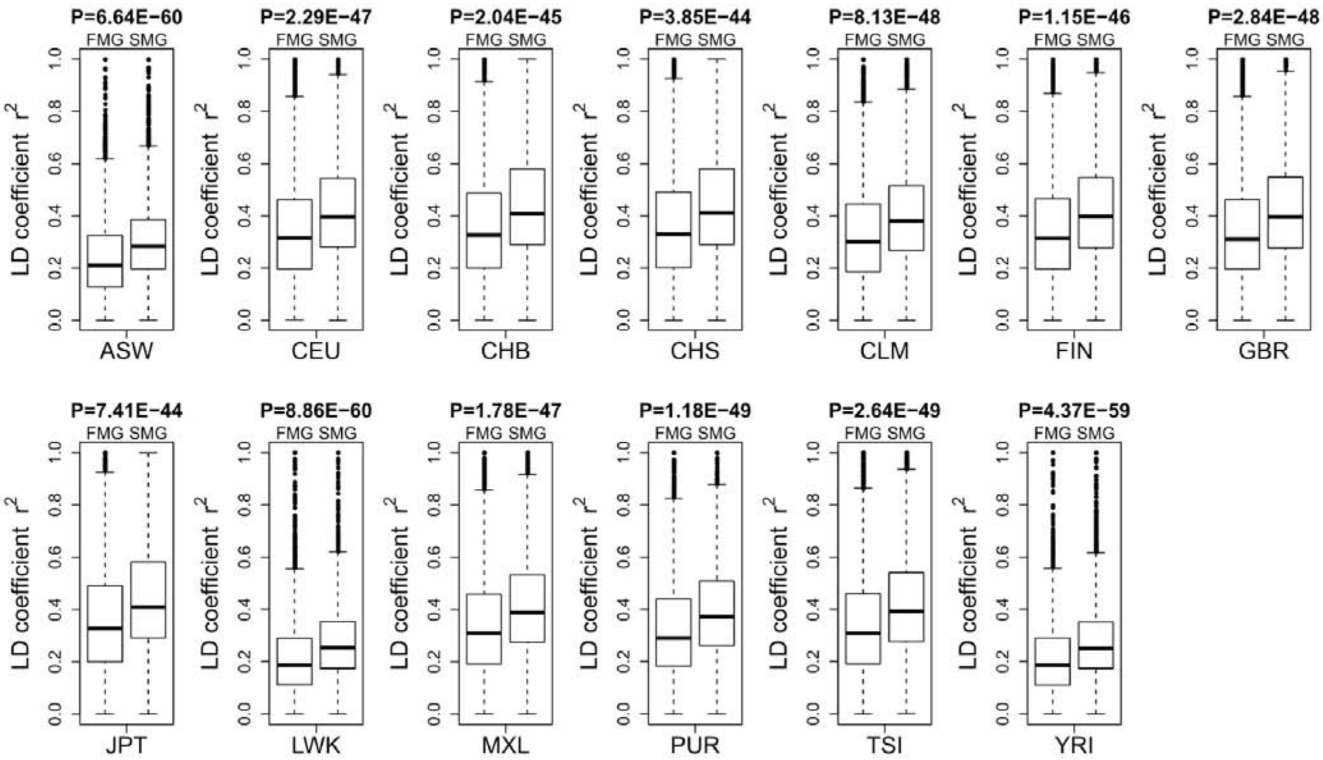

Supplementary Figure S6-10: The box plots of the SM genes against the FM genes in the LD coefficient r2 for each of the 13 populations of 1000 Genomes Project.

Supplementary Table S6-1: Summary statistics for the comparisons of dn/ds in 21 species

Supplementary Table S6-2: Summary statistics for the comparisons of sequence identity in 21 species

Supplementary Table S6-3: Summary statistics for the comparisons of r^2 in HapMap population

Supplementary Table S6-4: Summary statistics for the comparisons of r^2 in 1000 genomes populations

SECTION 7: COMPARISON OF EVOLUTIONARY CONSERVATION BETWEEN SM (TOP 20%) AND FM GENES (BOTTOM 20%) USING INTRON DATA

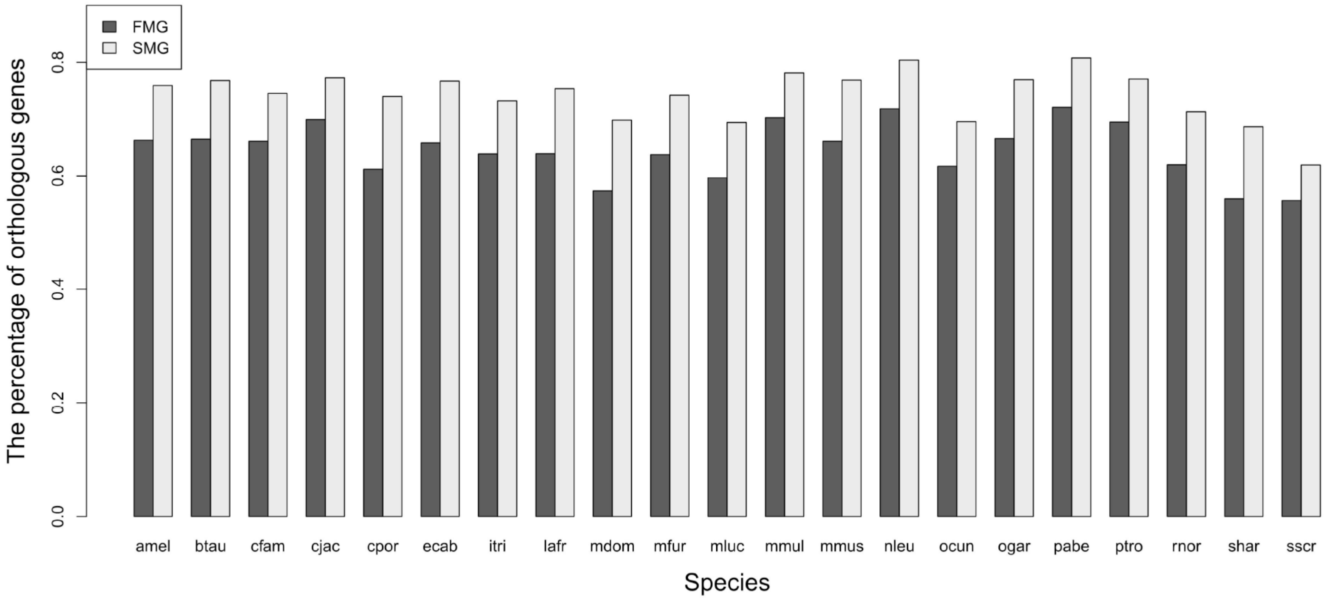

Supplementary Figure S7-1: The bar plots of the SM genes against the FM genes in the percentage of orthologous genes for each of the 21 species.

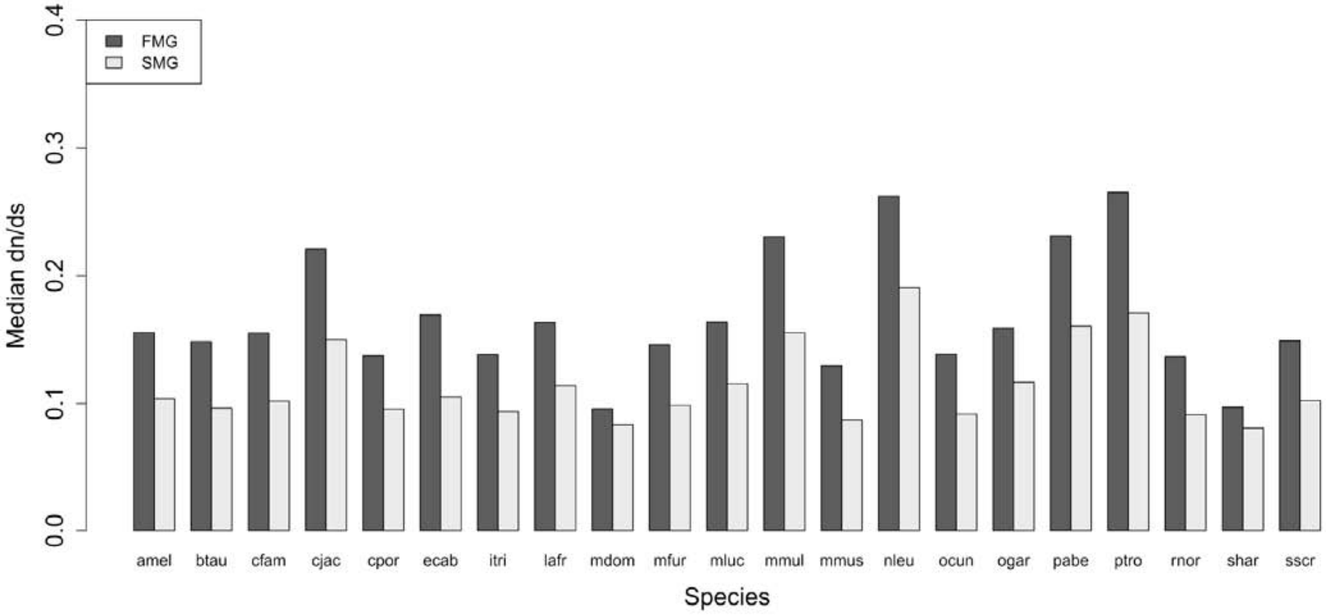

Supplementary Figure S7-2: The bar plots of the SM genes against the FM genes in the evolutionary rate for each of the 21 species.

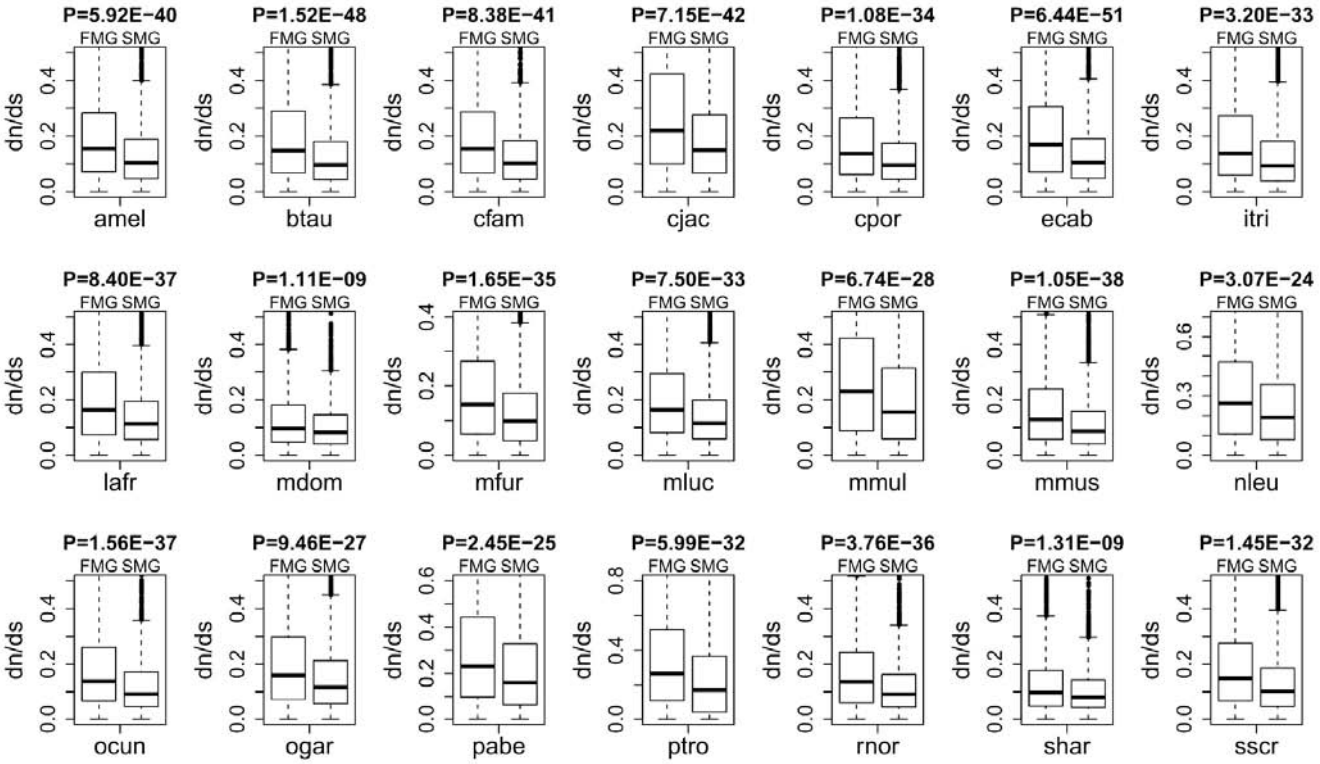

Supplementary Figure S7-3: The box plots of the SM genes against the FM genes in the evolutionary rate for each of the 21 species.

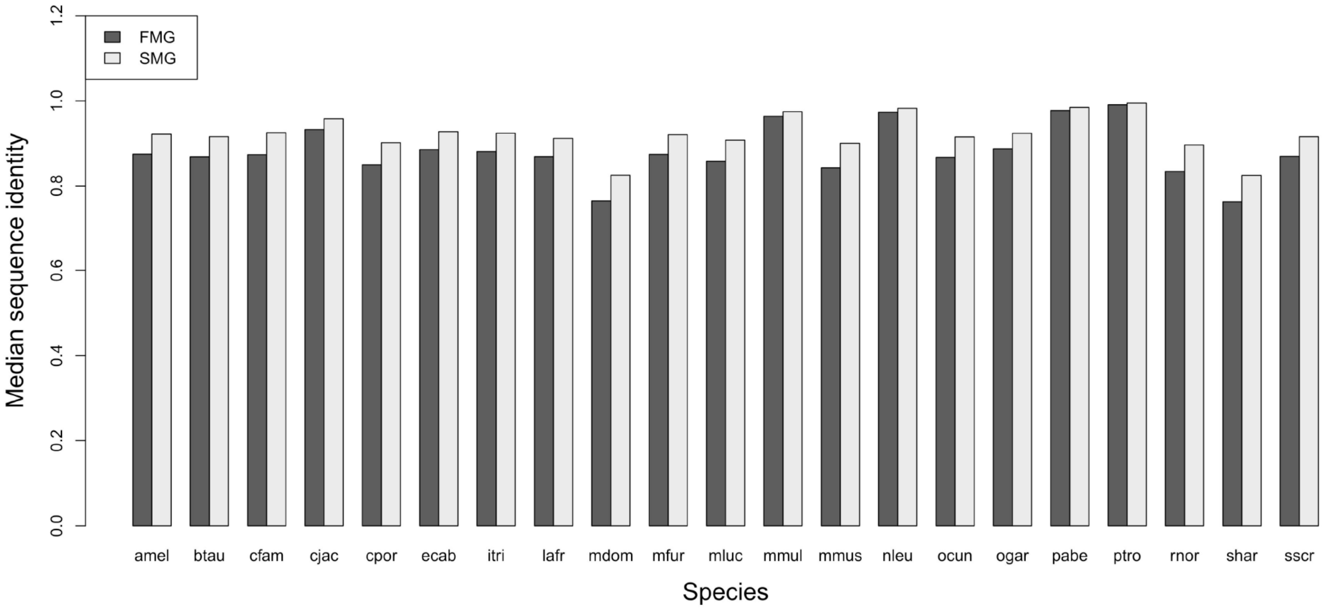

Supplementary Figure S7-4: The bar plots of the SM genes against the FM genes in the sequence identity for each of the 21 species.

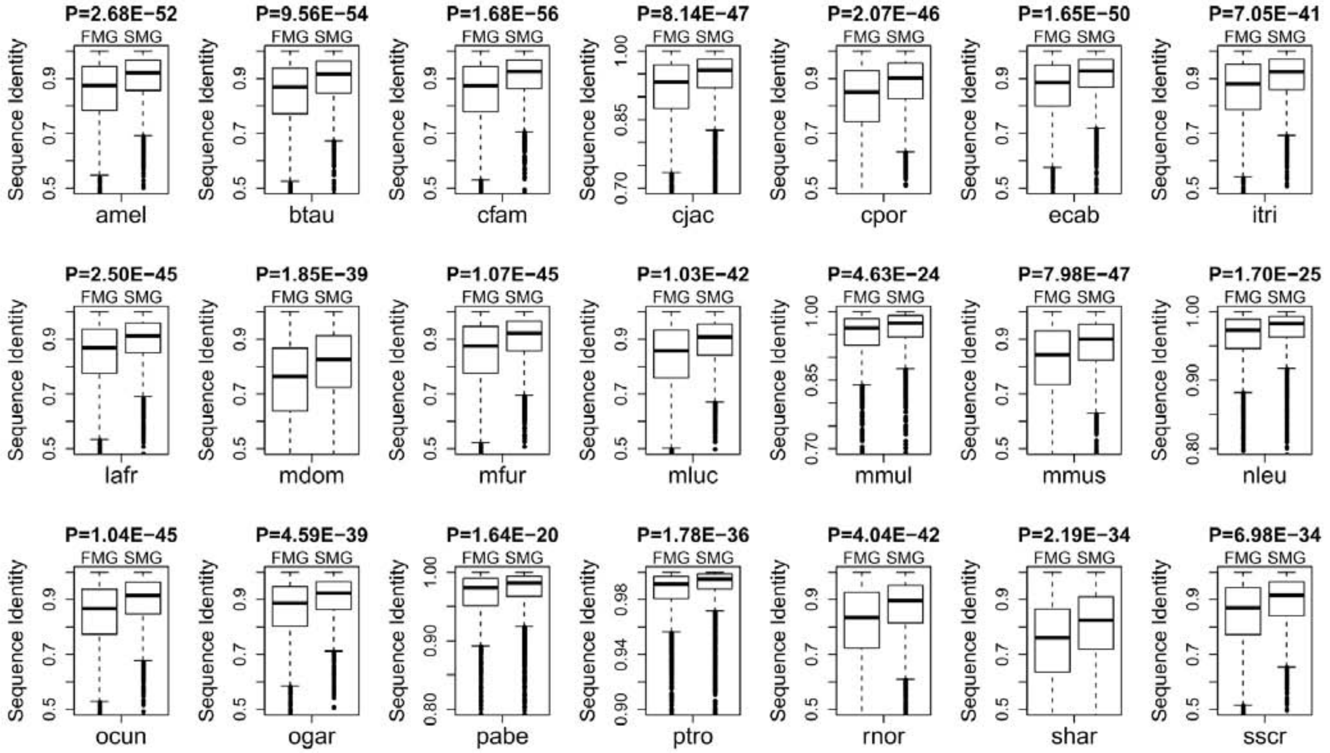

Supplementary Figure S7-5: The box plots of the SM genes against the FM genes in the sequence identity for each of the 21 species.

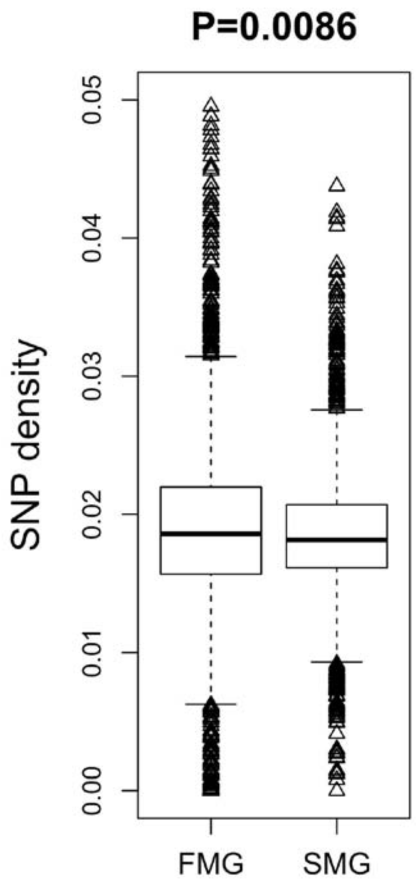

Supplementary Figure S7-6: The box plot of the SM genes against the FM genes in the SNP density.

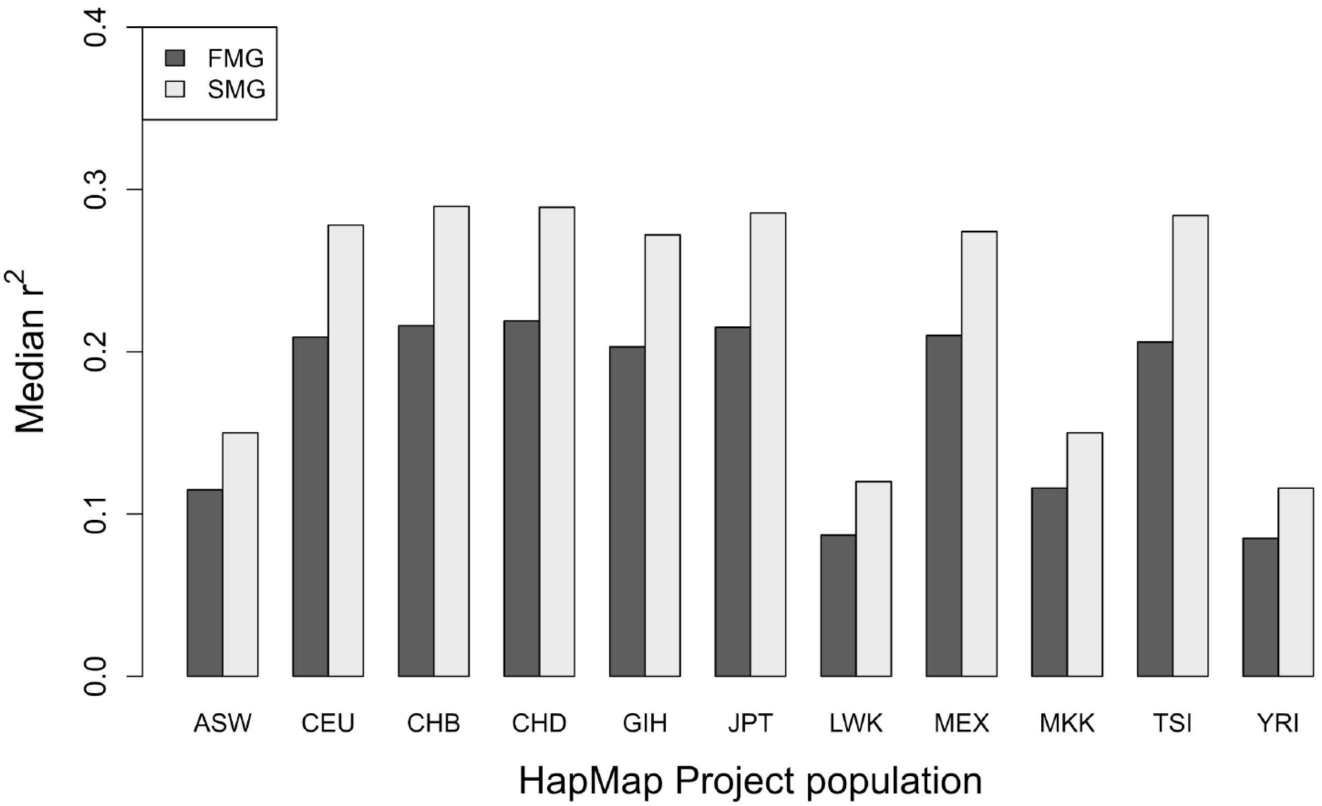

Supplementary Figure S7-7: The bar plots of the SM genes against the FM genes in the LD coefficient r2 for each of the 11 populations of HapMap Project.

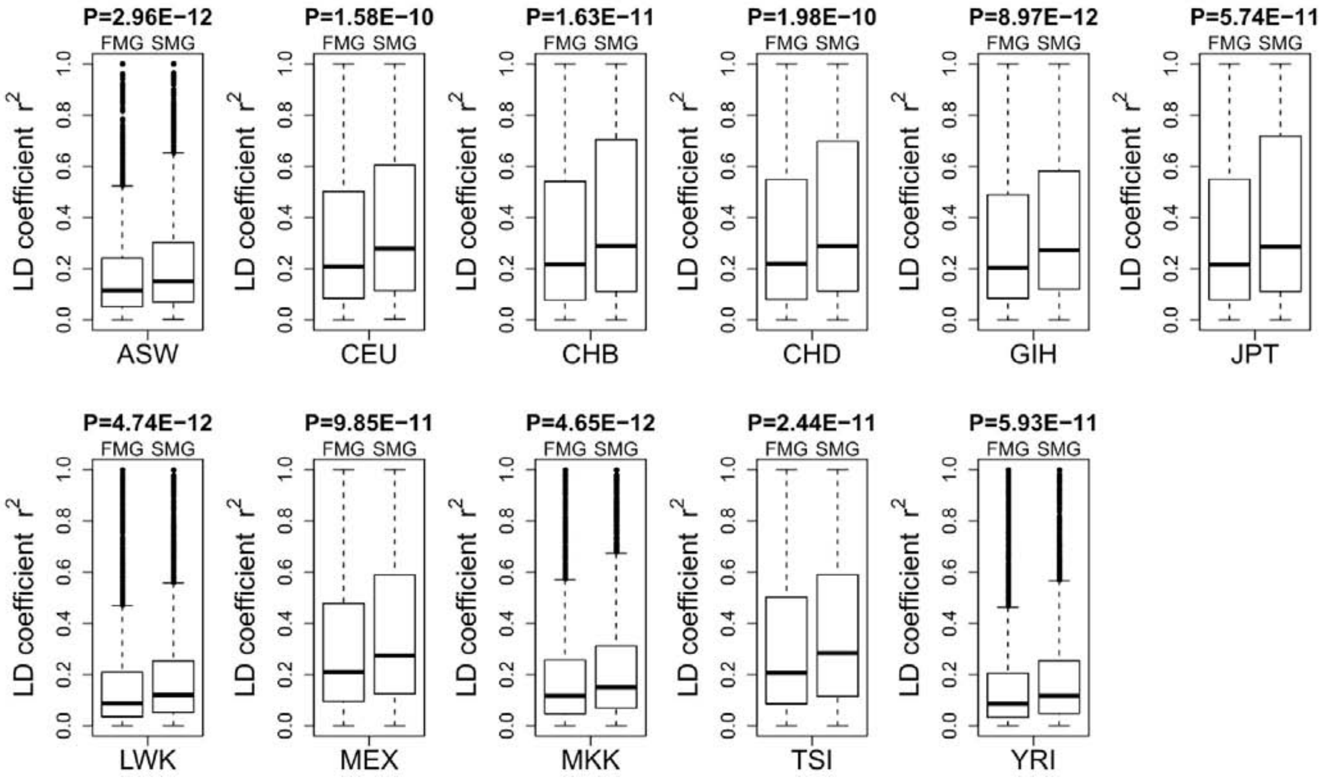

Supplementary Figure S7-8: The box plots of the SM genes against the FM genes in the LD coefficient r2 for each of the 11 populations of HapMap Project.

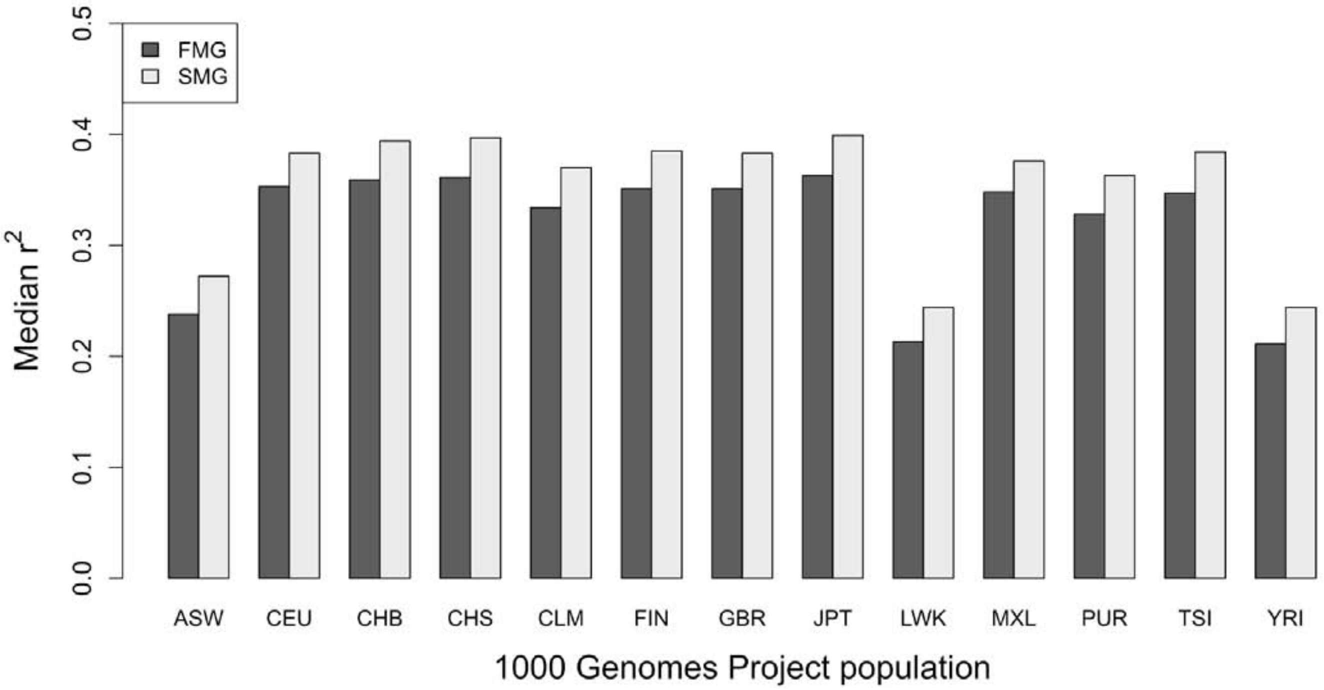

Supplementary Figure S7-9: The bar plots of the SM genes against the FM genes in the LD coefficient r2 for each of the 13 populations of 1000 Genomes Project.

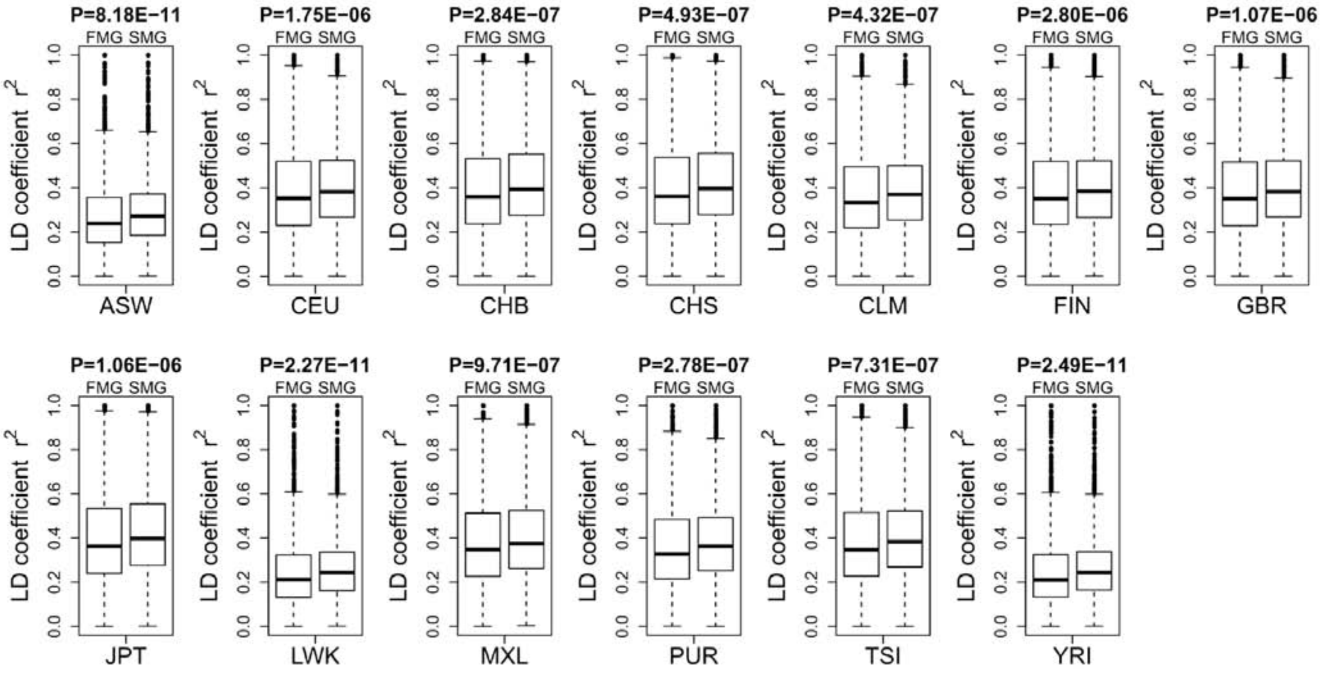

Supplementary Figure S7-10: The box plots of the SM genes against the FM genes in the LD coefficient r2 for each of the 13 populations of 1000 Genomes Project.

Supplementary Table S7-1: Summary statistics for the comparisons of dn/ds in 21 species

Supplementary Table S7-2: Summary statistics for the comparisons of sequence identity in 21 species

Supplementary Table S7-3: Summary statistics for the comparisons of r^2 in HapMap population

Supplementary Table S7-4: Summary statistics for the comparisons of r^2 in 1000 genomes populations

SECTION 8: COMPARISON OF EVOLUTIONARY CONSERVATION BETWEEN SM (TOP 20%) AND FM GENES (BOTTOM 20%) USING 3'UTR DATA

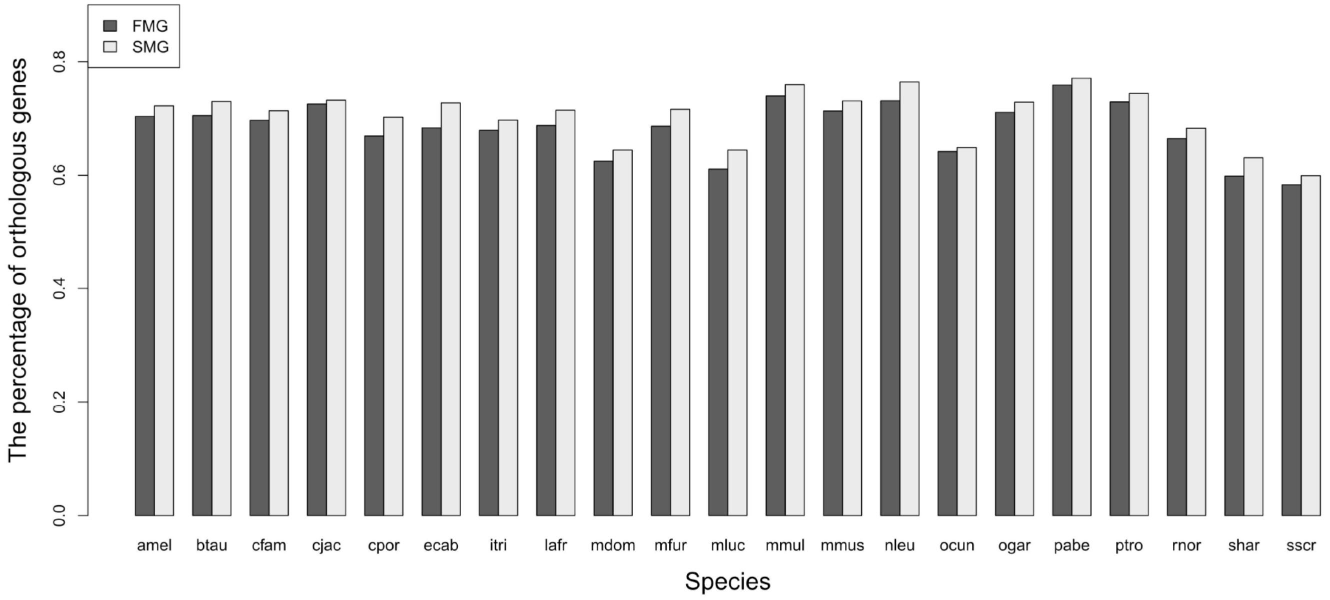

Supplementary Figure S8-1: The bar plots of the SM genes against the FM genes in the percentage of orthologous genes for each of the 21 species.

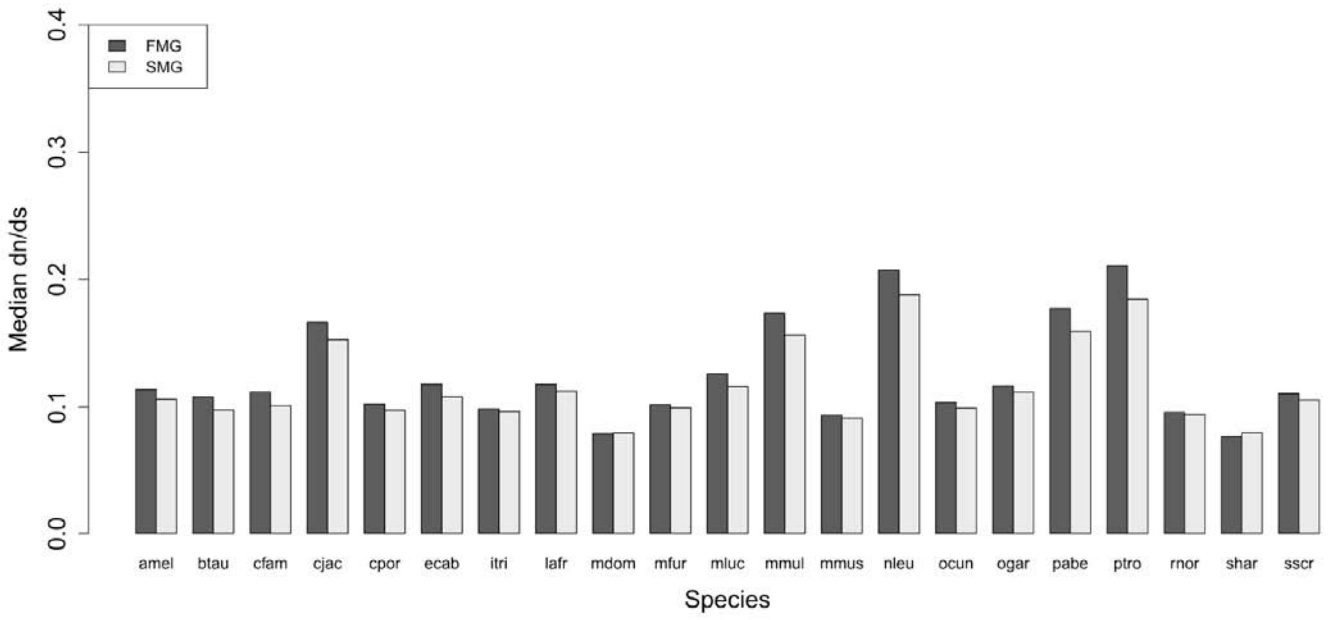

Supplementary Figure S8-2: The bar plots of the SM genes against the FM genes in the evolutionary rate for each of the 21 species.

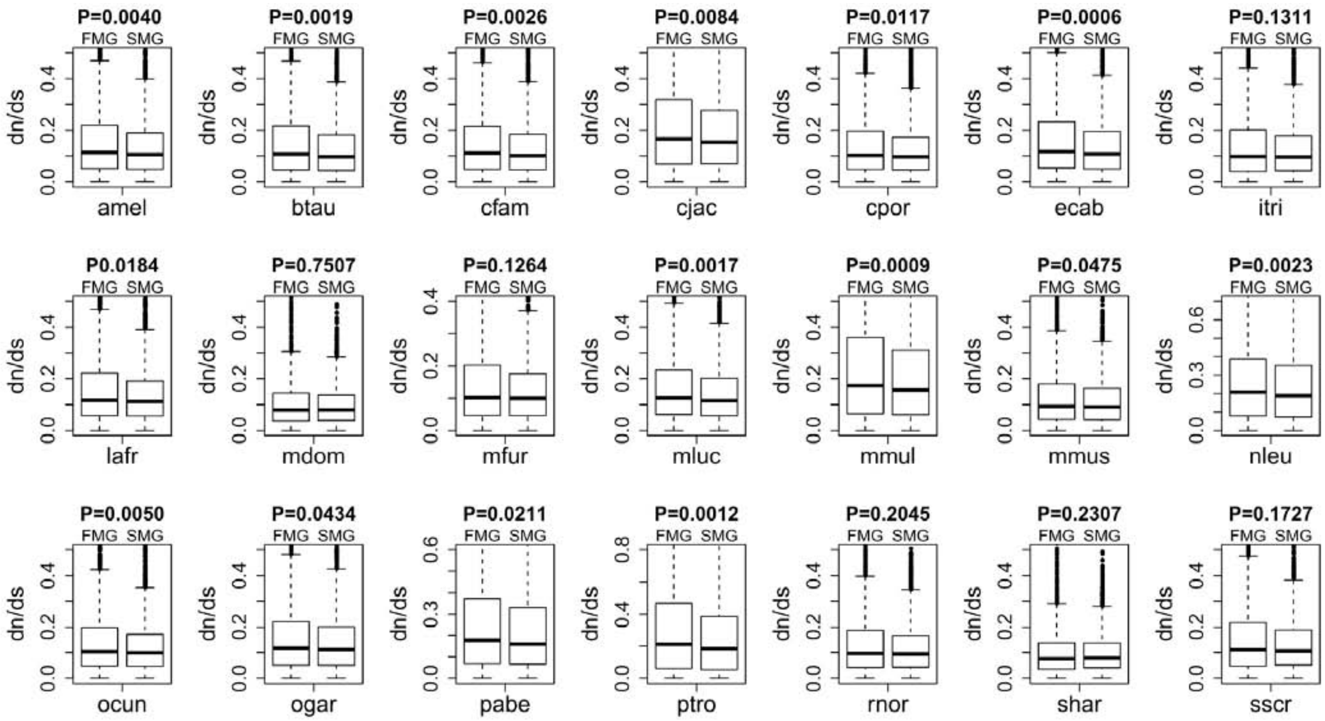

Supplementary Figure S8-3: The box plots of the SM genes against the FM genes in the evolutionary rate for each of the 21 species.

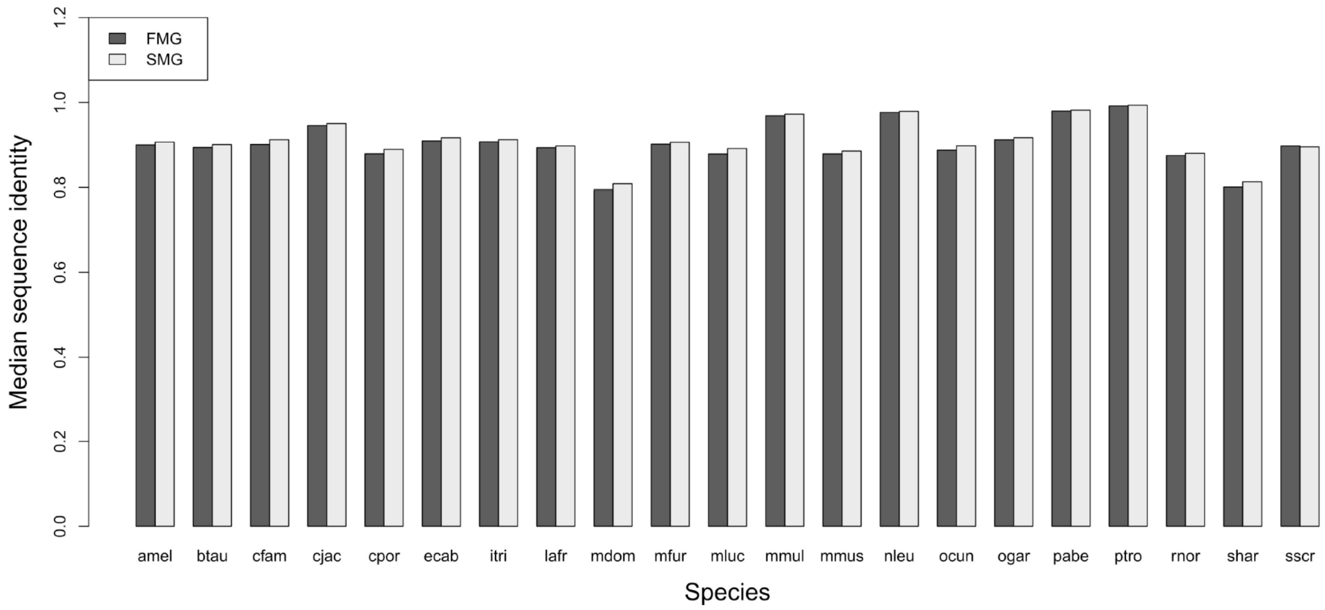

Supplementary Figure S8-4: The bar plots of the SM genes against the FM genes in the sequence identity for each of the 21 species.

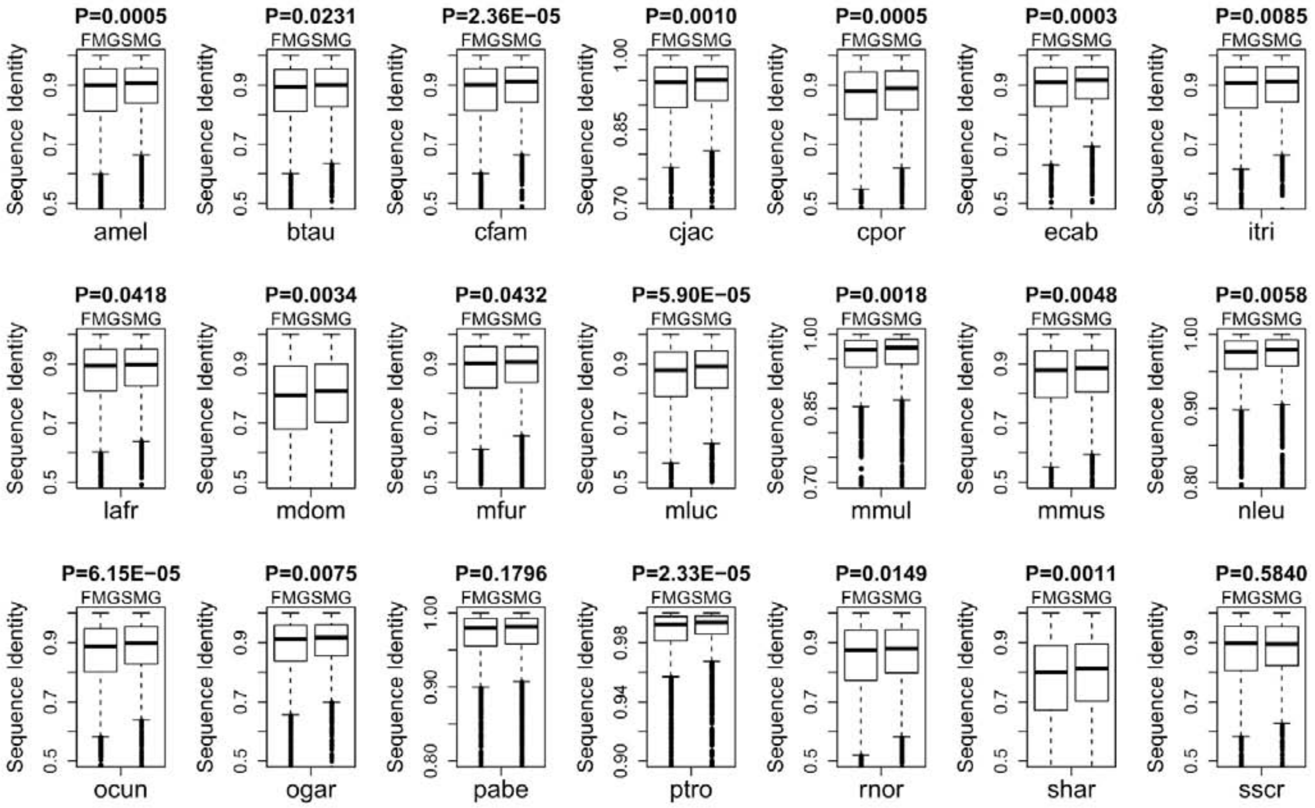

Supplementary Figure S8-5: The box plots of the SM genes against the FM genes in the sequence identity for each of the 21 species.

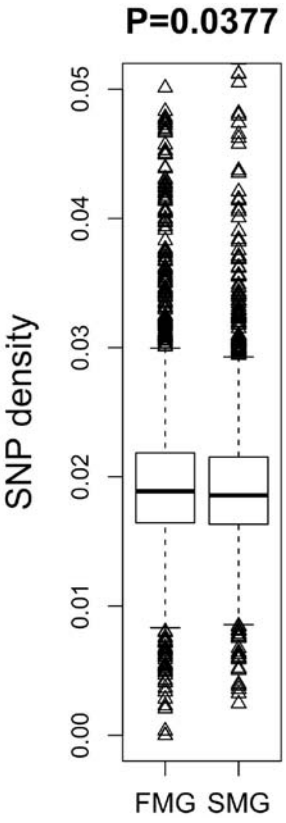

Supplementary Figure S8-6: The box plot of the SM genes against the FM genes in the SNP density.

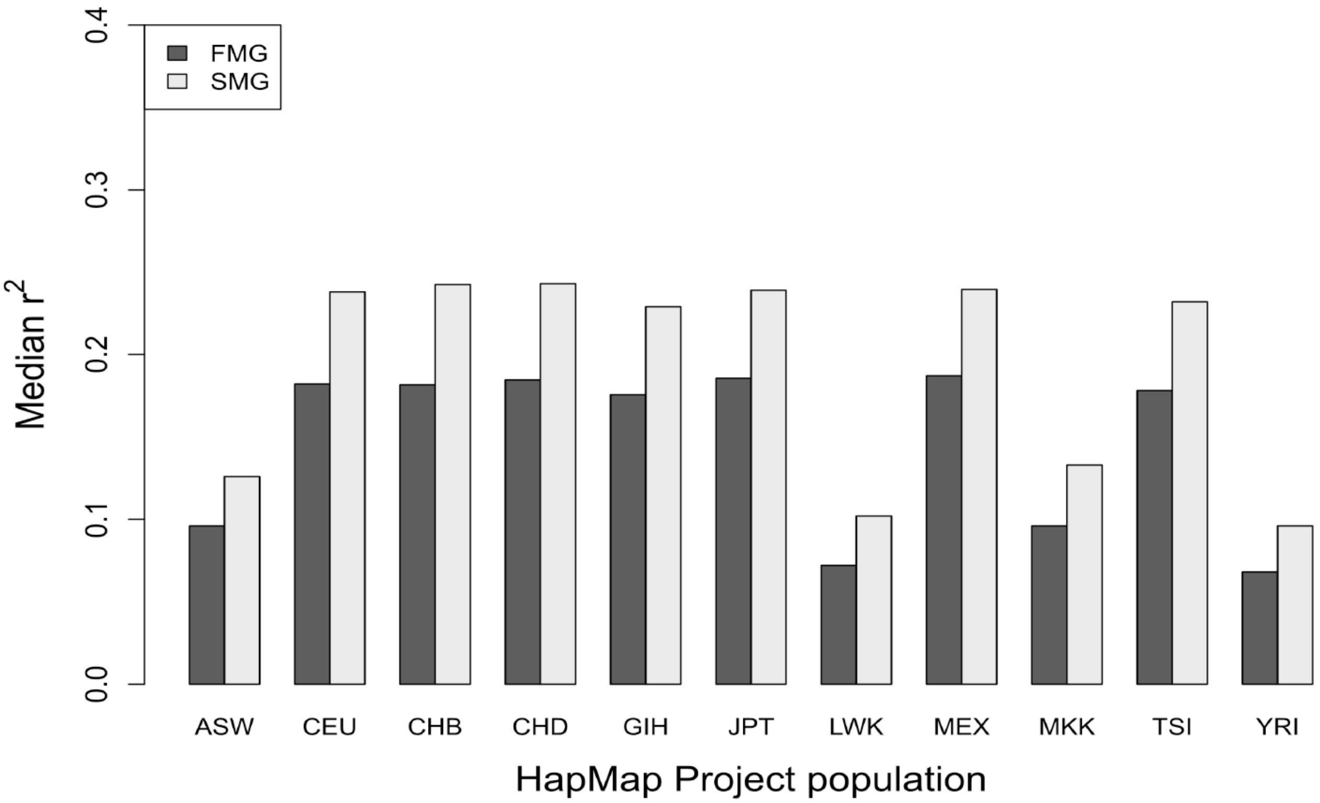

Supplementary Figure S8-7: The bar plots of the SM genes against the FM genes in the LD coefficient r2 for each of the 11 populations of HapMap Project.

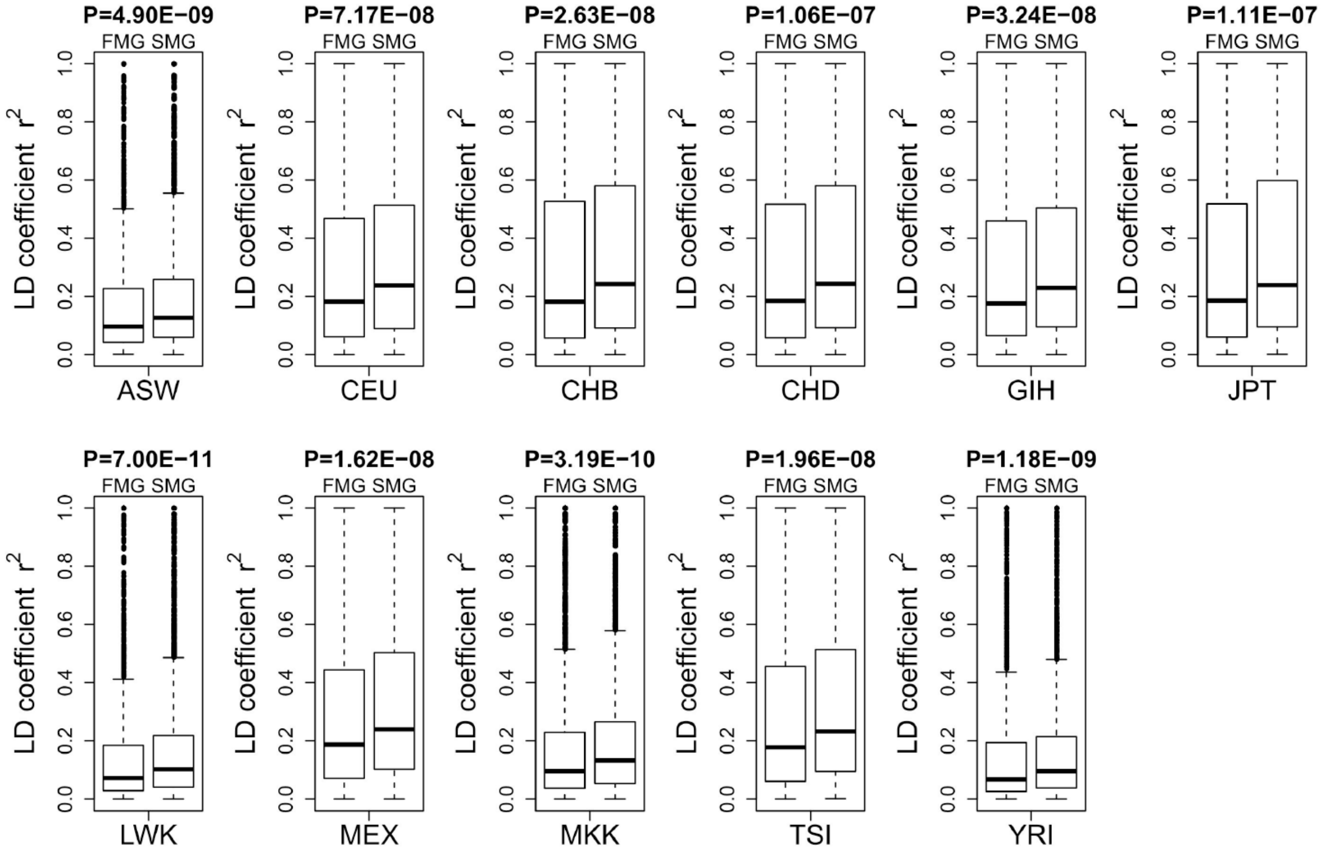

Supplementary Figure S8-8: The box plots of the SM genes against the FM genes in the LD coefficient r2 for each of the 11 populations of HapMap Project.

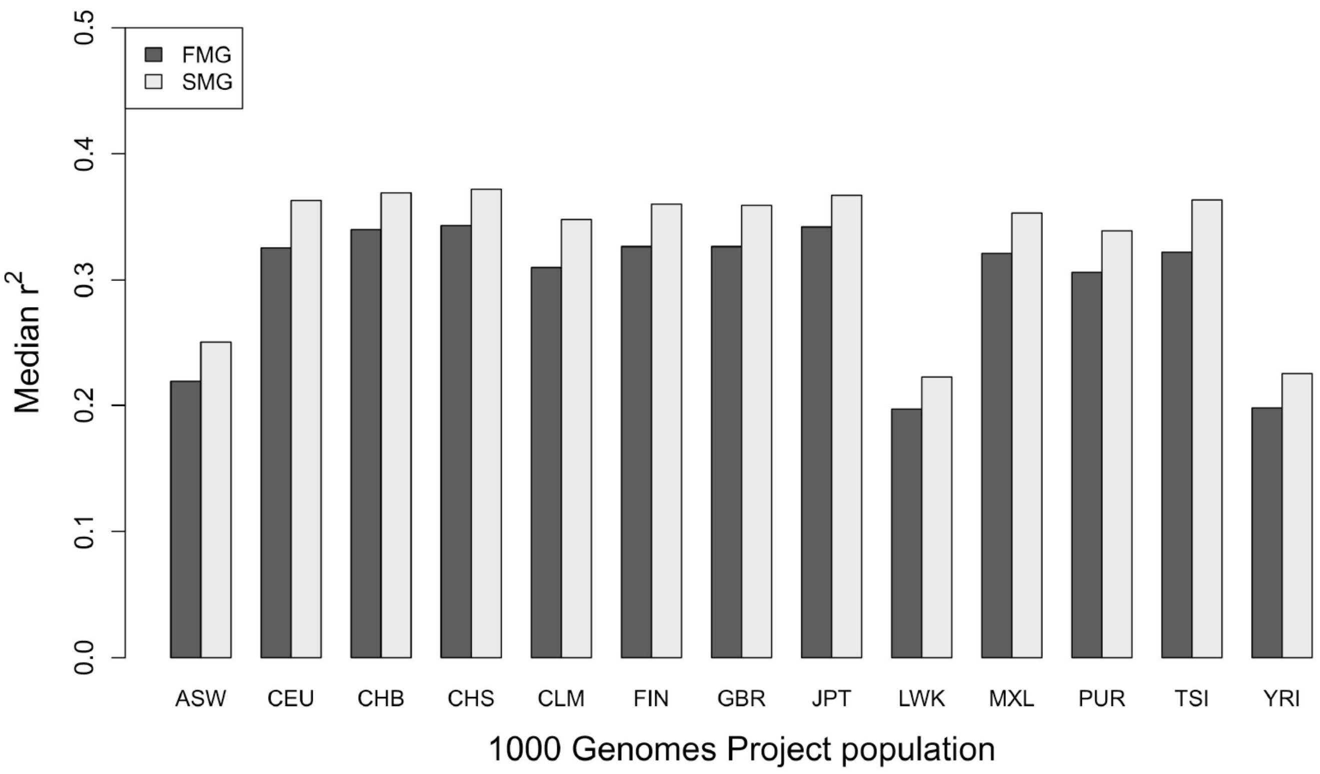

Supplementary Figure S8-9: The bar plots of the SM genes against the FM genes in the LD coefficient r2 for each of the 13 populations of 1000 Genomes Project.

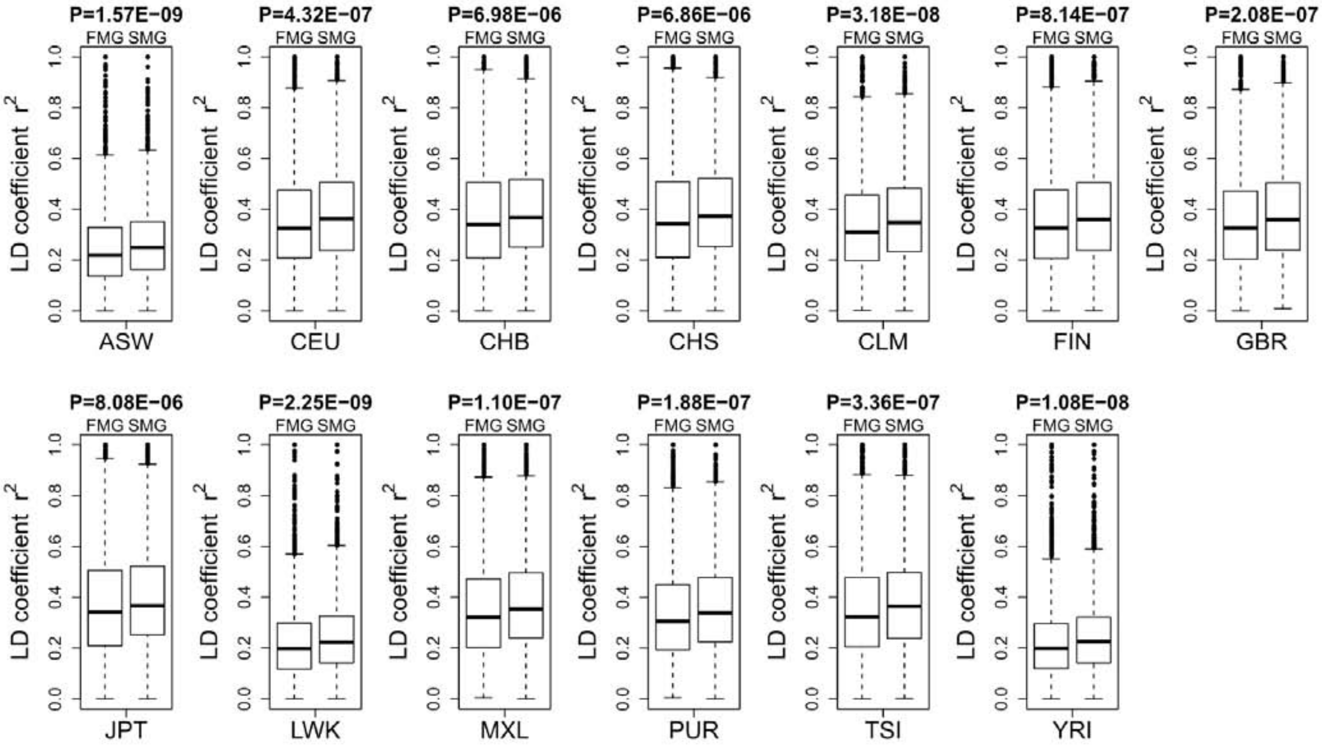

Supplementary Figure S8-10: The box plots of the SM genes against the FM genes in the LD coefficient r2 for each of the 13 populations of 1000 Genomes Project.

Supplementary Table S8-1: Summary statistics for the comparisons of dn/ds in 21 species

Supplementary Table S8-2. Summary statistics for the comparisons of sequence identity in 21 species.

Supplementary Table S8-3: Summary statistics for the comparisons of r^2 in HapMap population

Supplementary Table S8-4: Summary statistics for the comparisons of r^2 in 1000 genomes populations

SECTION 9: COMPARISON OF EVOLUTIONARY CONSERVATION BETWEEN SM (TOP 20%) AND FM GENES (BOTTOM 20%) USING 5'UTR DATA

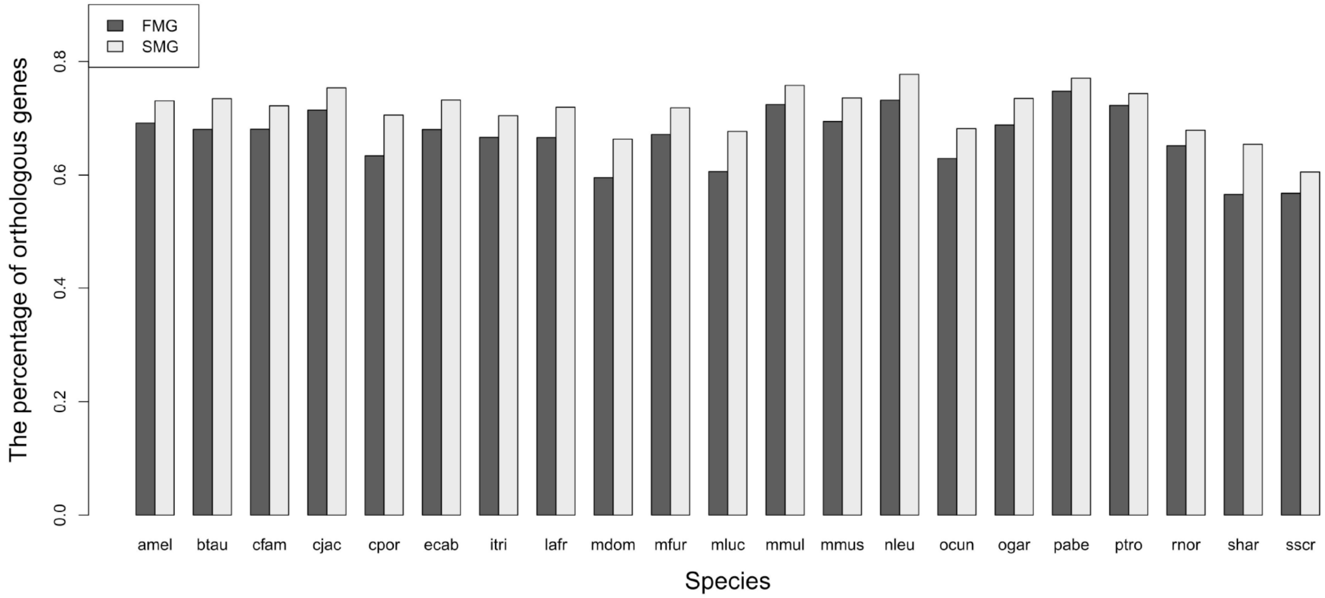

Supplementary Figure S9-1: The bar plots of the SM genes against the FM genes in the percentage of orthologous genes for each of the 21 species.

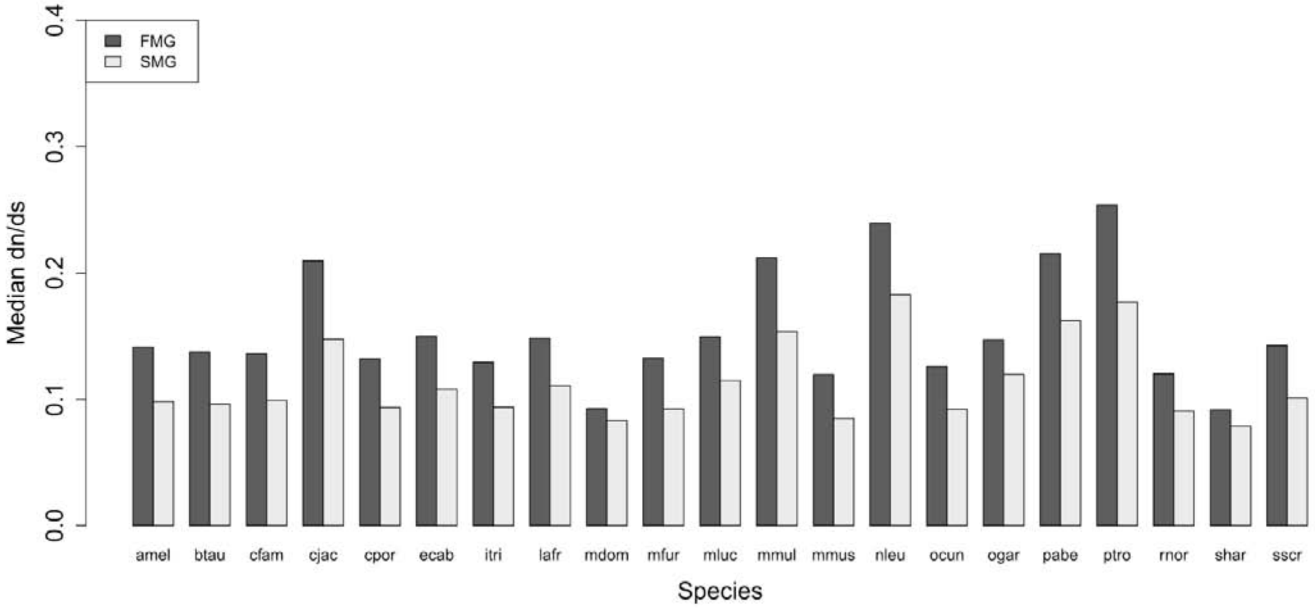

Supplementary Figure S9-2: The bar plots of the SM genes against the FM genes in the evolutionary rate for each of the 21 species.

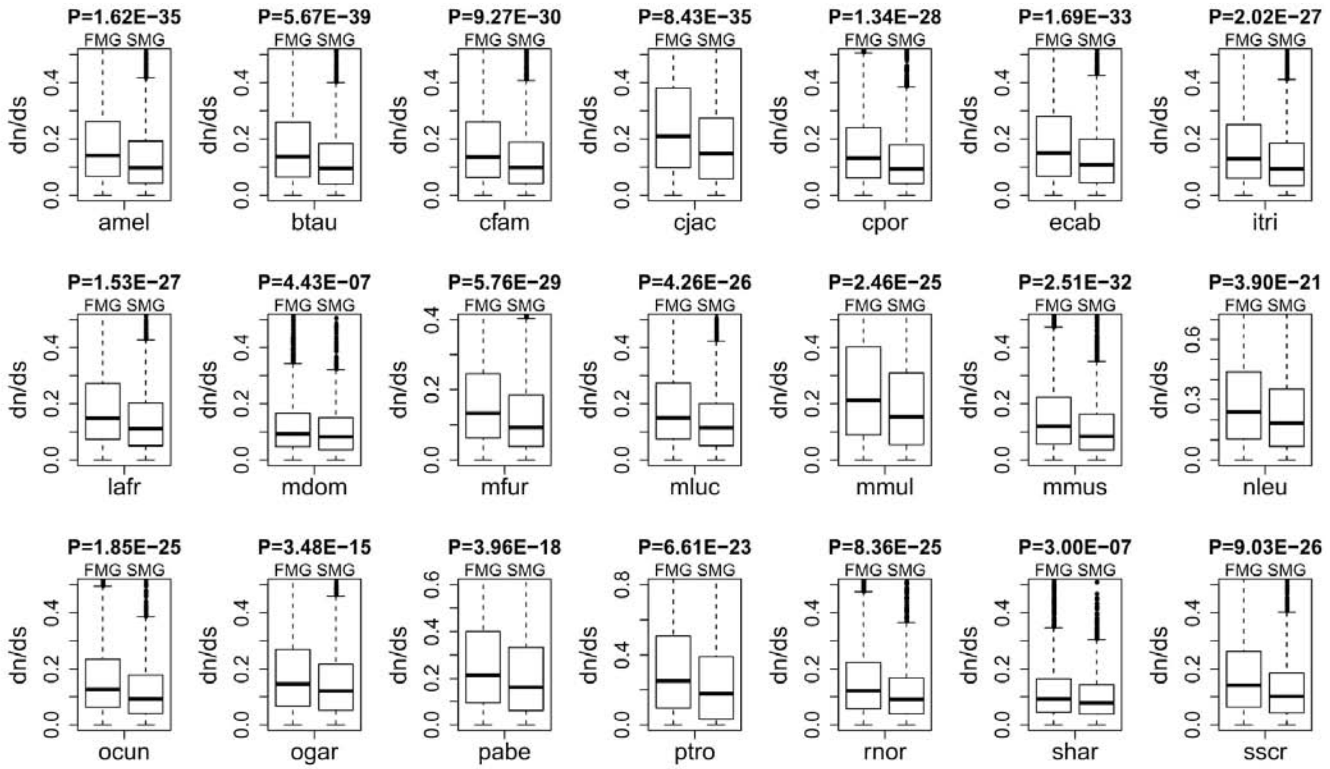

Supplementary Figure S9-3: The box plots of the SM genes against the FM genes in the evolutionary rate for each of the 21 species.

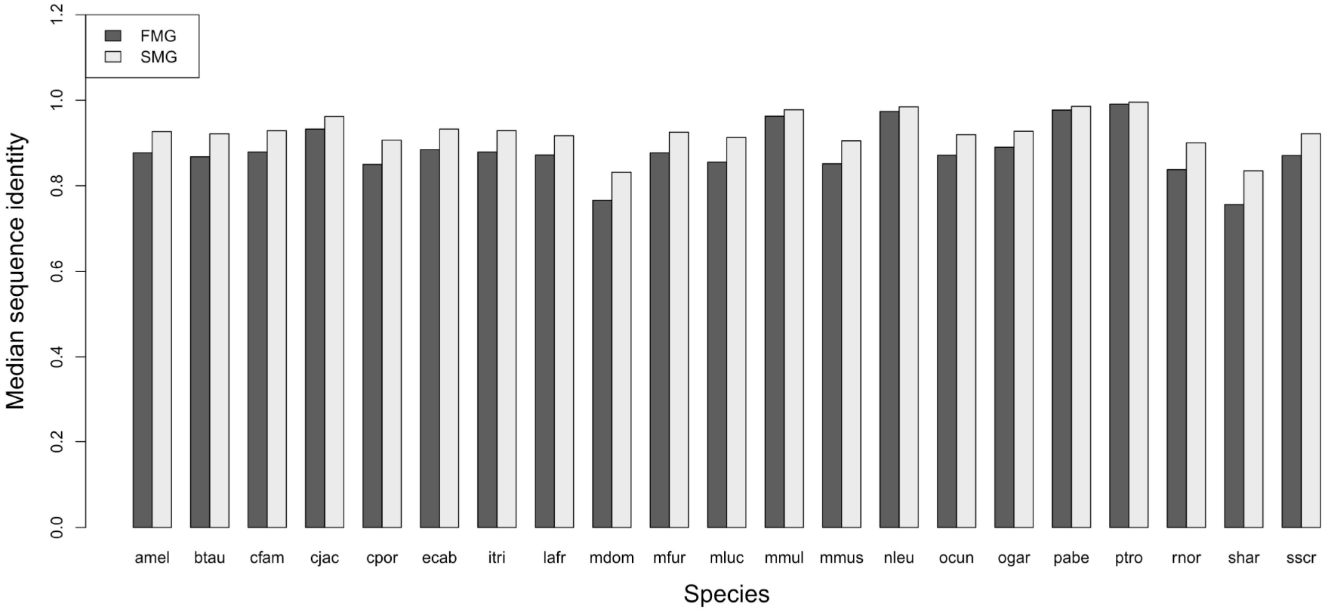

Supplementary Figure S9-4: The bar plots of the SM genes against the FM genes in the sequence identity for each of the 21 species.

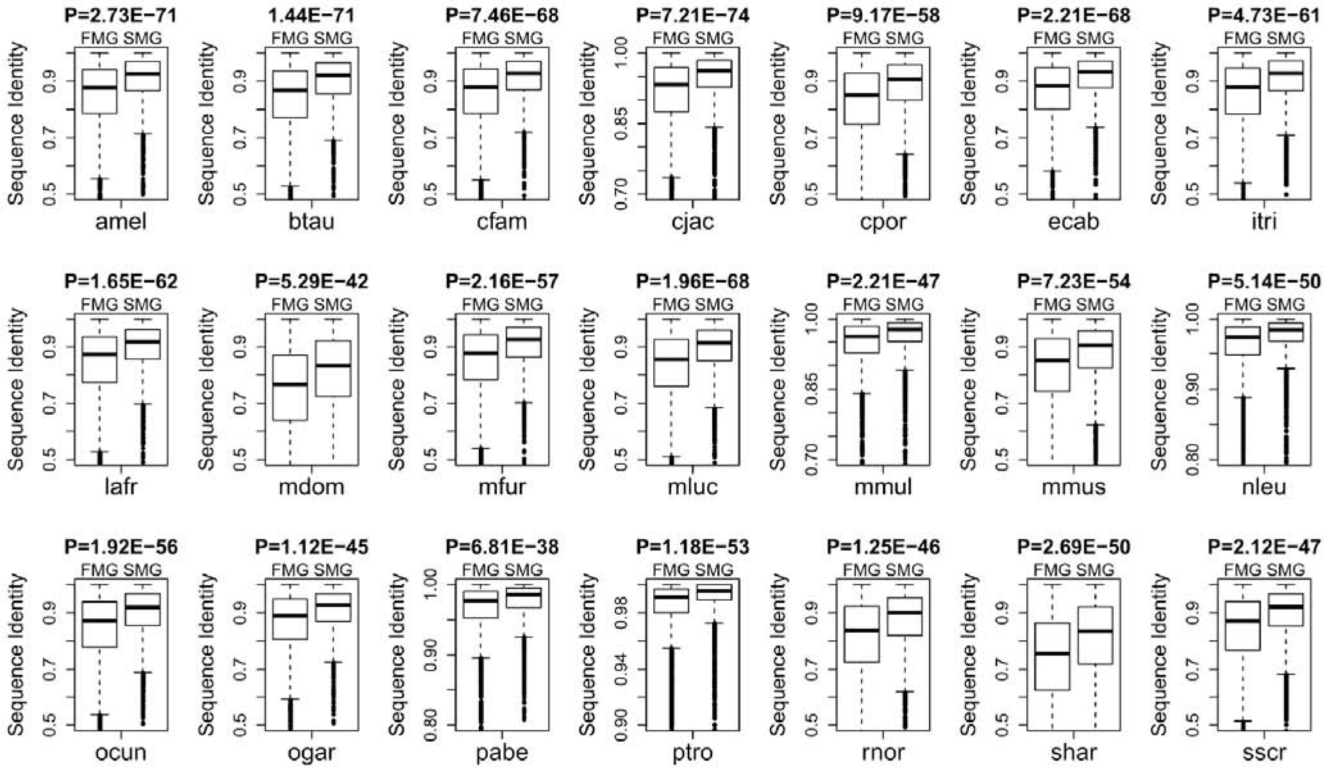

Supplementary Figure S9-5: The box plots of the SM genes against the FM genes in the sequence identity for each of the 21 species.

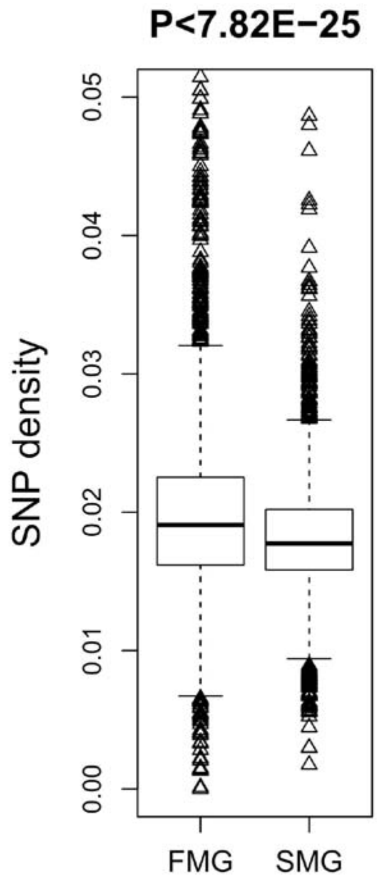

Supplementary Figure S9-6: The box plot of the SM genes against the FM genes in the SNP density.

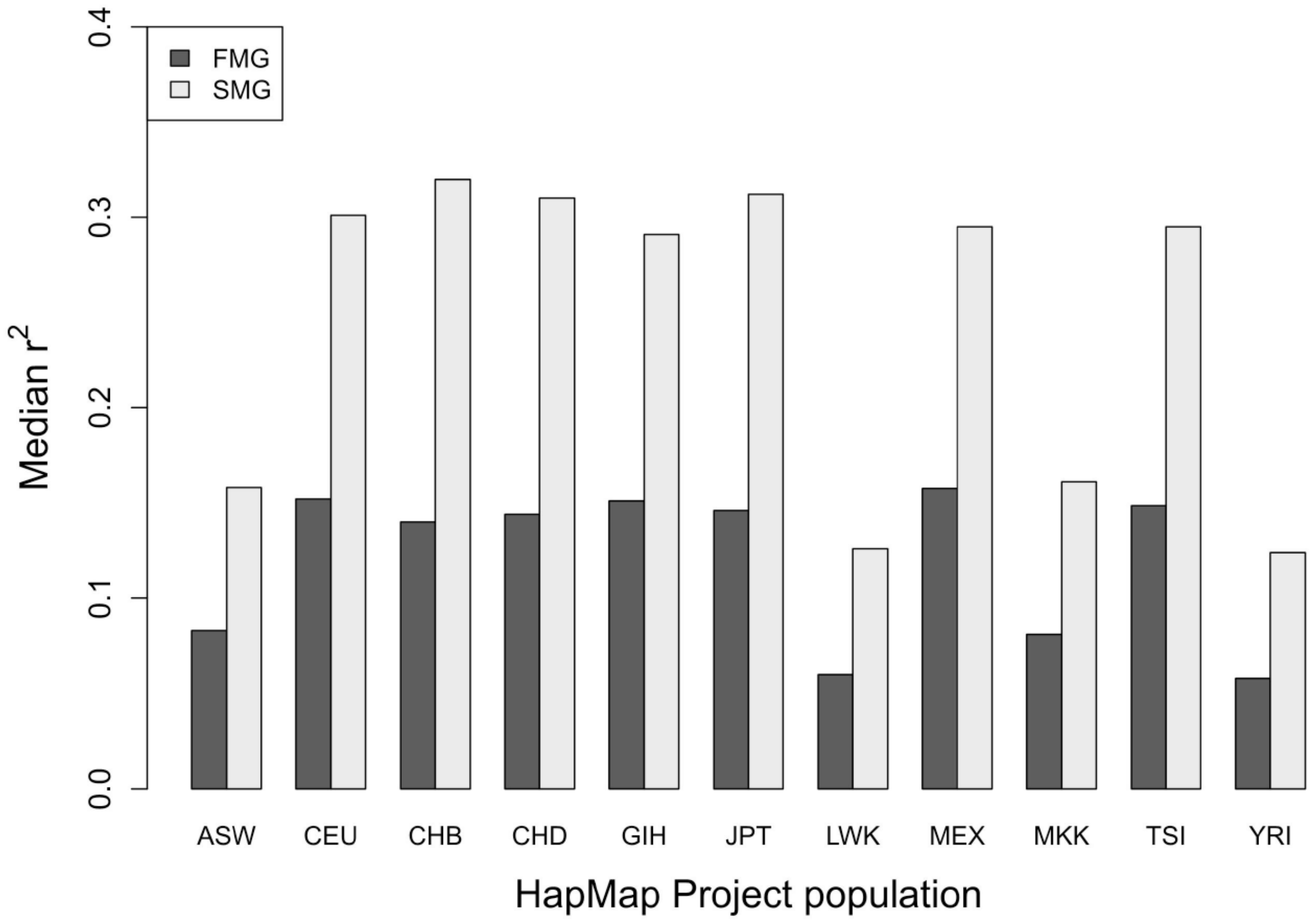

Supplementary Figure S9-7: The bar plots of the SM genes against the FM genes in the LD coefficient r2 for each of the 11 populations of HapMap Project.

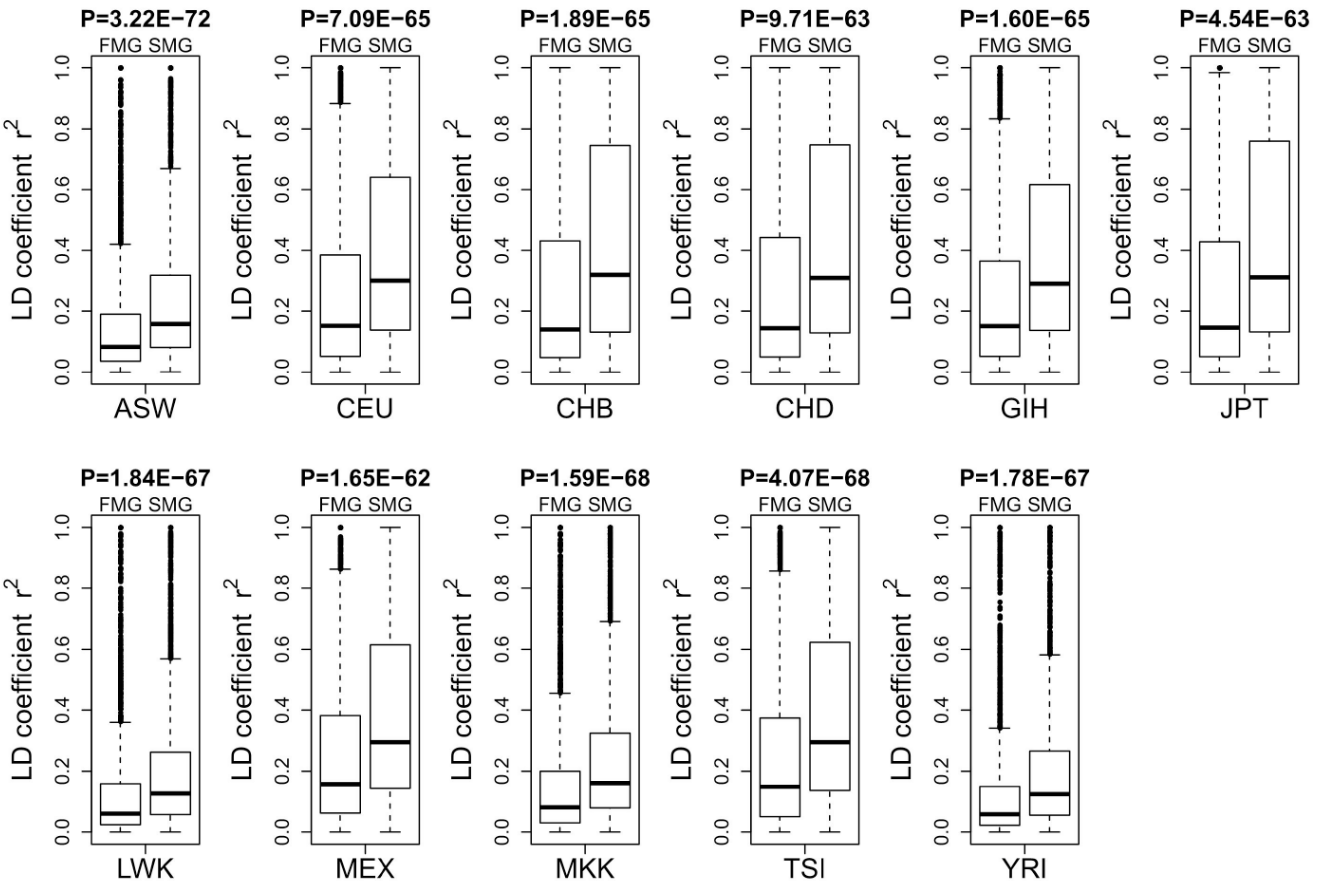

Supplementary Figure S9-8: The box plots of the SM genes against the FM genes in the LD coefficient r2 for each of the 11 populations of HapMap Project.

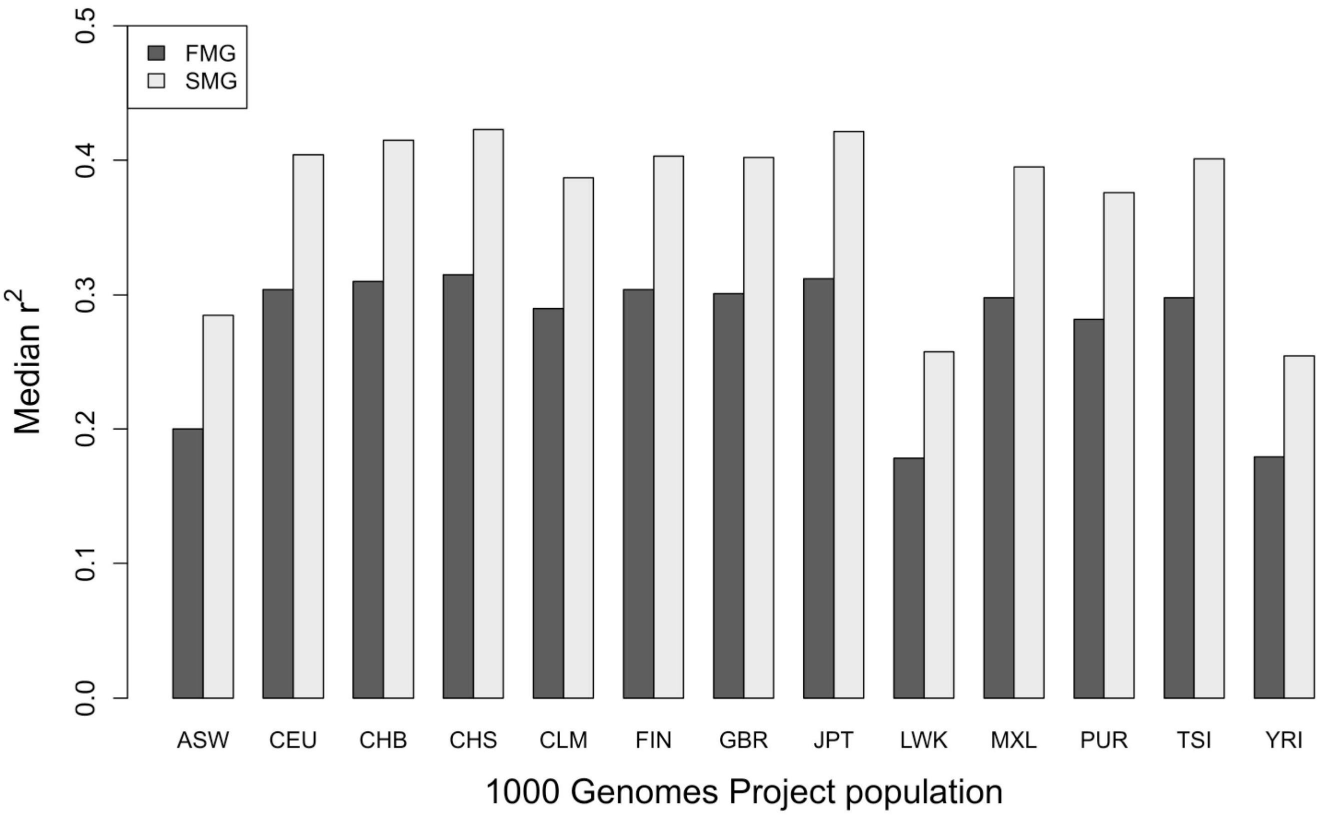

Supplementary Figure S9-9: The bar plots of the SM genes against the FM genes in the LD coefficient r2 for each of the 13 populations of 1000 Genomes Project.

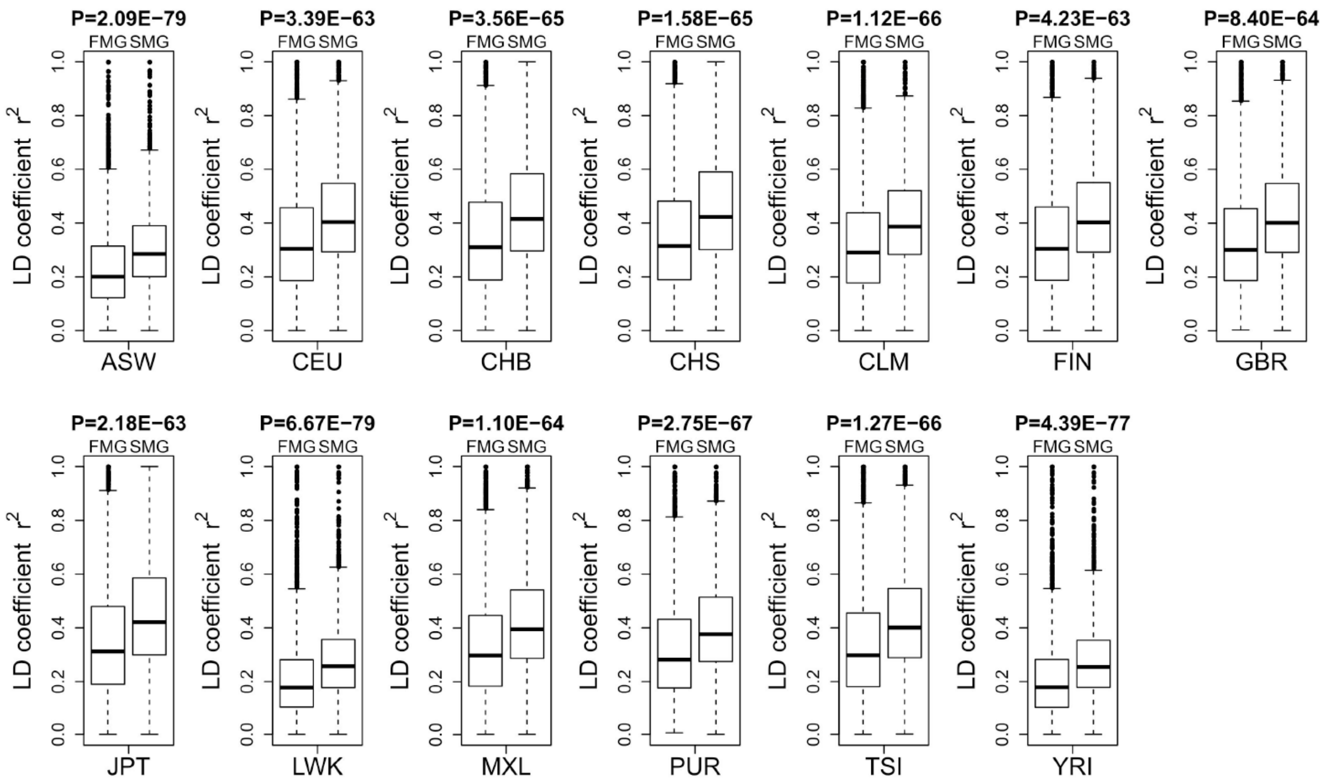

Supplementary Figure S9-10: The box plots of the SM genes against the FM genes in the LD coefficient r2 for each of the 13 populations of 1000 Genomes Project.

Supplementary Table S9-1: Summary statistics for the comparisons of dn/ds in 21 species

Supplementary Table S9-2. Summary statistics for the comparisons of sequence identity in 21 species.

Supplementary Table S9-3: Summary statistics for the comparisons of r^2 in HapMap population

Supplementary Table S9-4: Summary statistics for the comparisons of r^2 in 1000 genomes populations

SECTION 10: COMPARISON OF EVOLUTIONARY CONSERVATION BETWEEN SM (TOP 20%) AND FM GENES (BOTTOM 20%) USING TSS1500 DATA

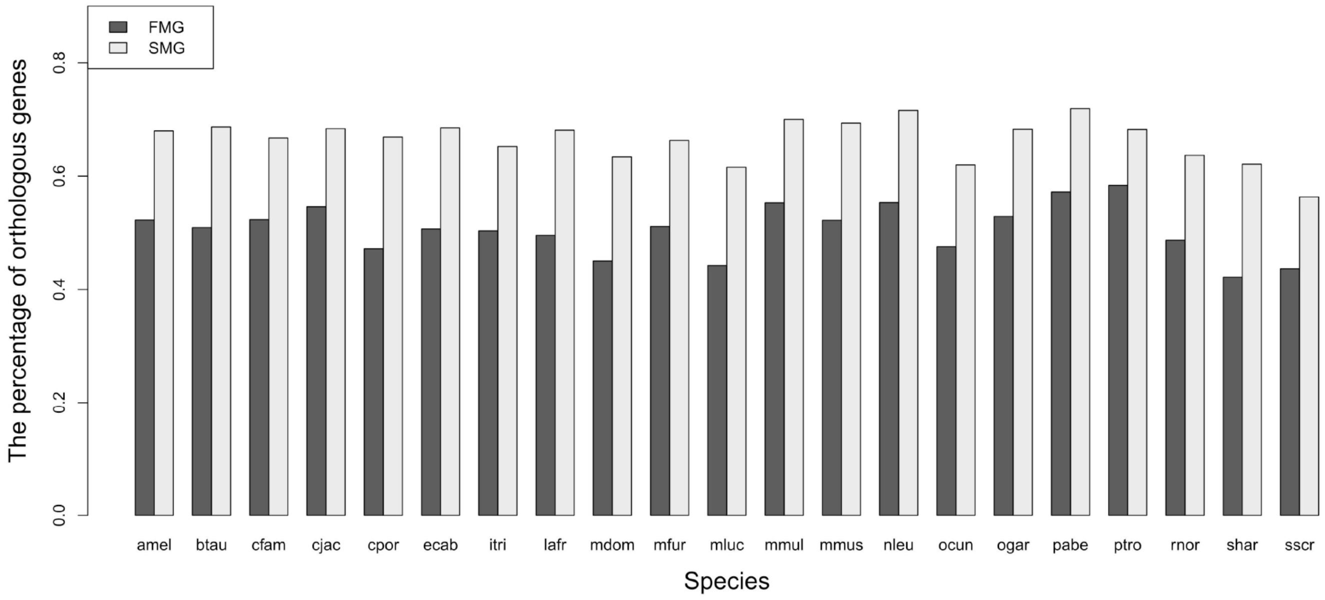

Supplementary Figure S10-1: The bar plots of the SM genes against the FM genes in the percentage of orthologous genes for each of the 21 species.

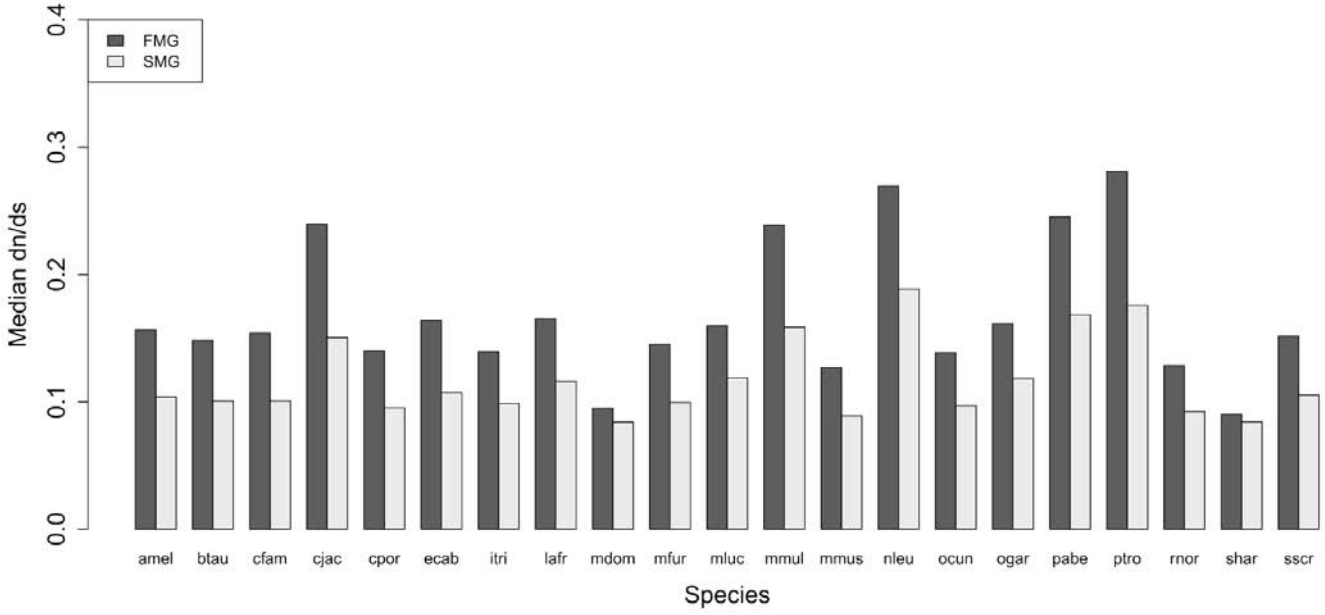

Supplementary Figure S10-2: The bar plots of the SM genes against the FM genes in the evolutionary rate for each of the 21 species.

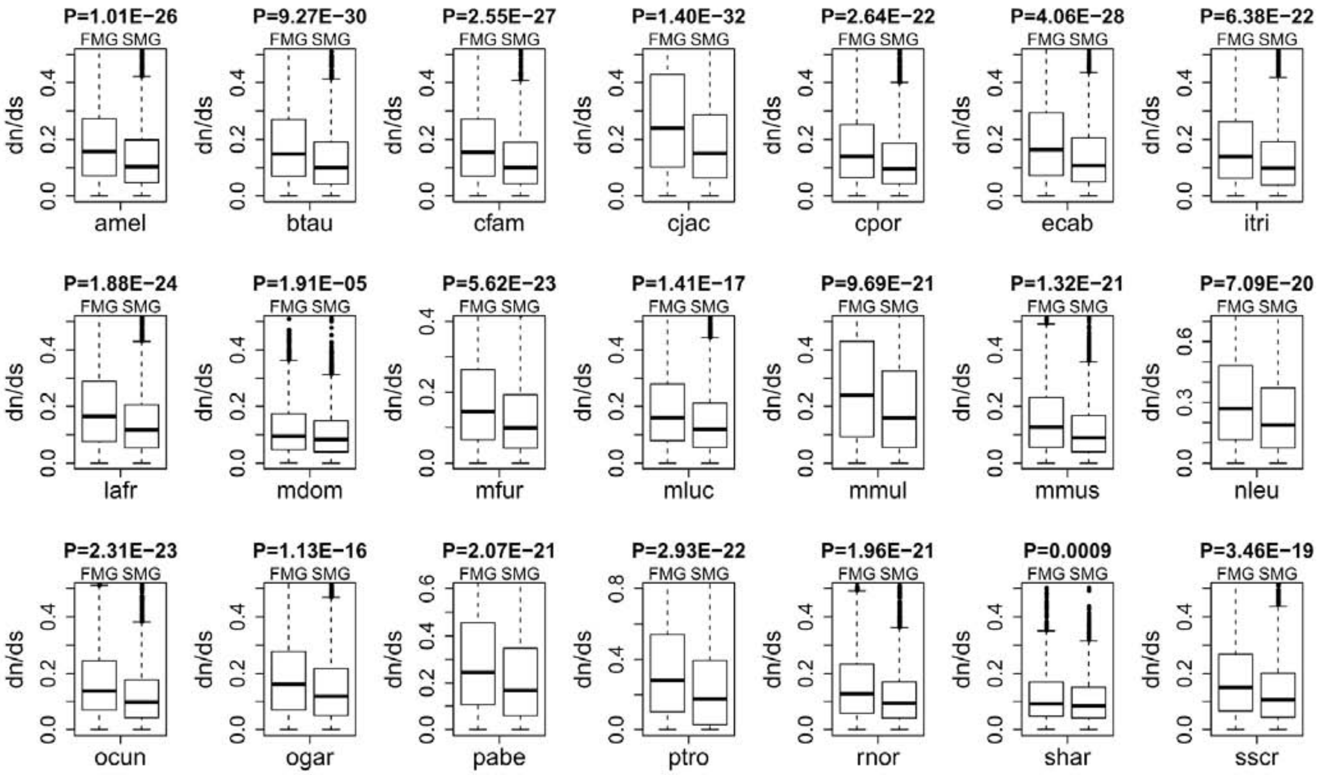

Supplementary Figure S10-3: The box plots of the SM genes against the FM genes in the evolutionary rate for each of the 21 species.

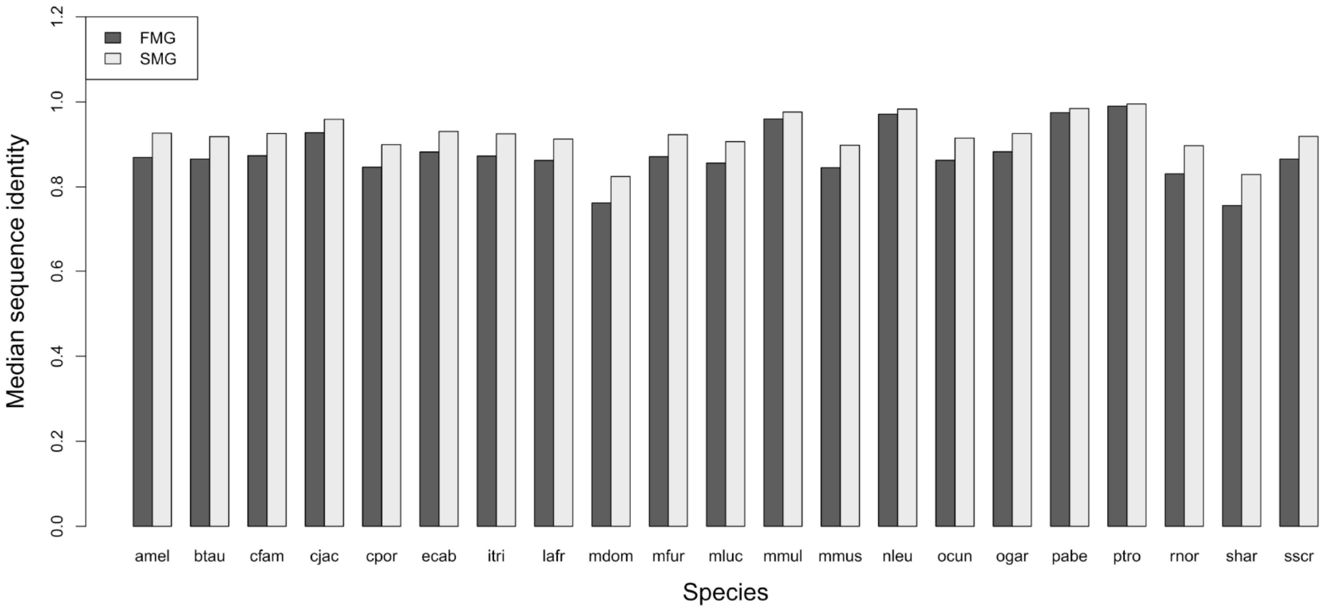

Supplementary Figure S10-4: The bar plots of the SM genes against the FM genes in the sequence identity for each of the 21 species.

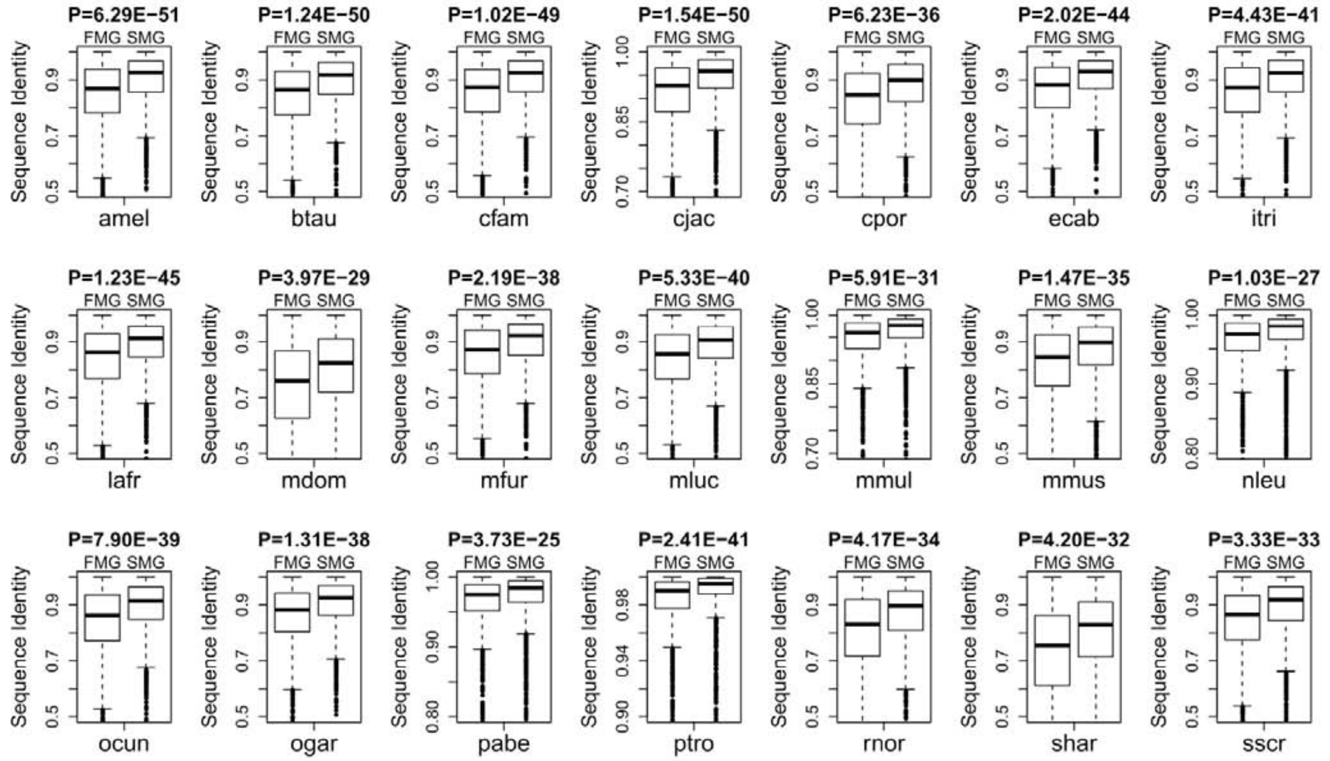

Supplementary Figure S10-5: The box plots of the SM genes against the FM genes in the sequence identity for each of the 21 species.

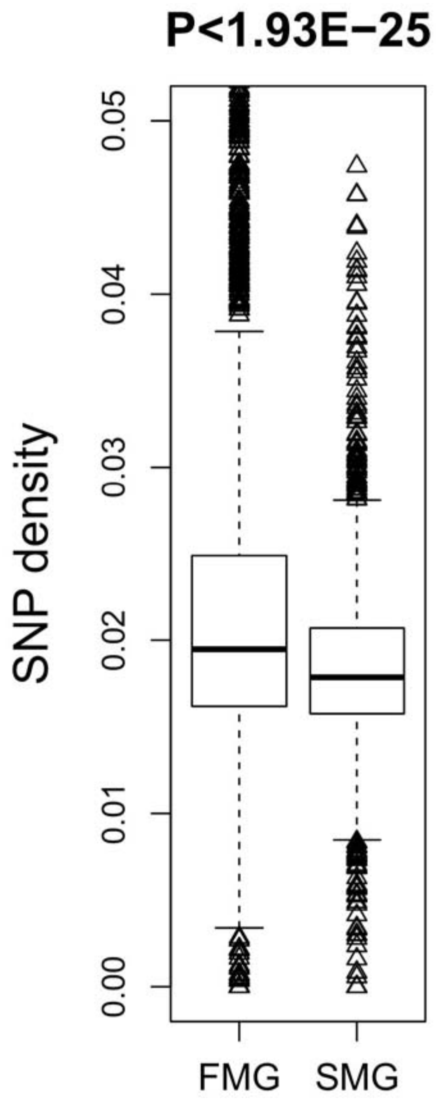

Supplementary Figure S10-6: The box plot of the SM genes against the FM genes in the SNP density.

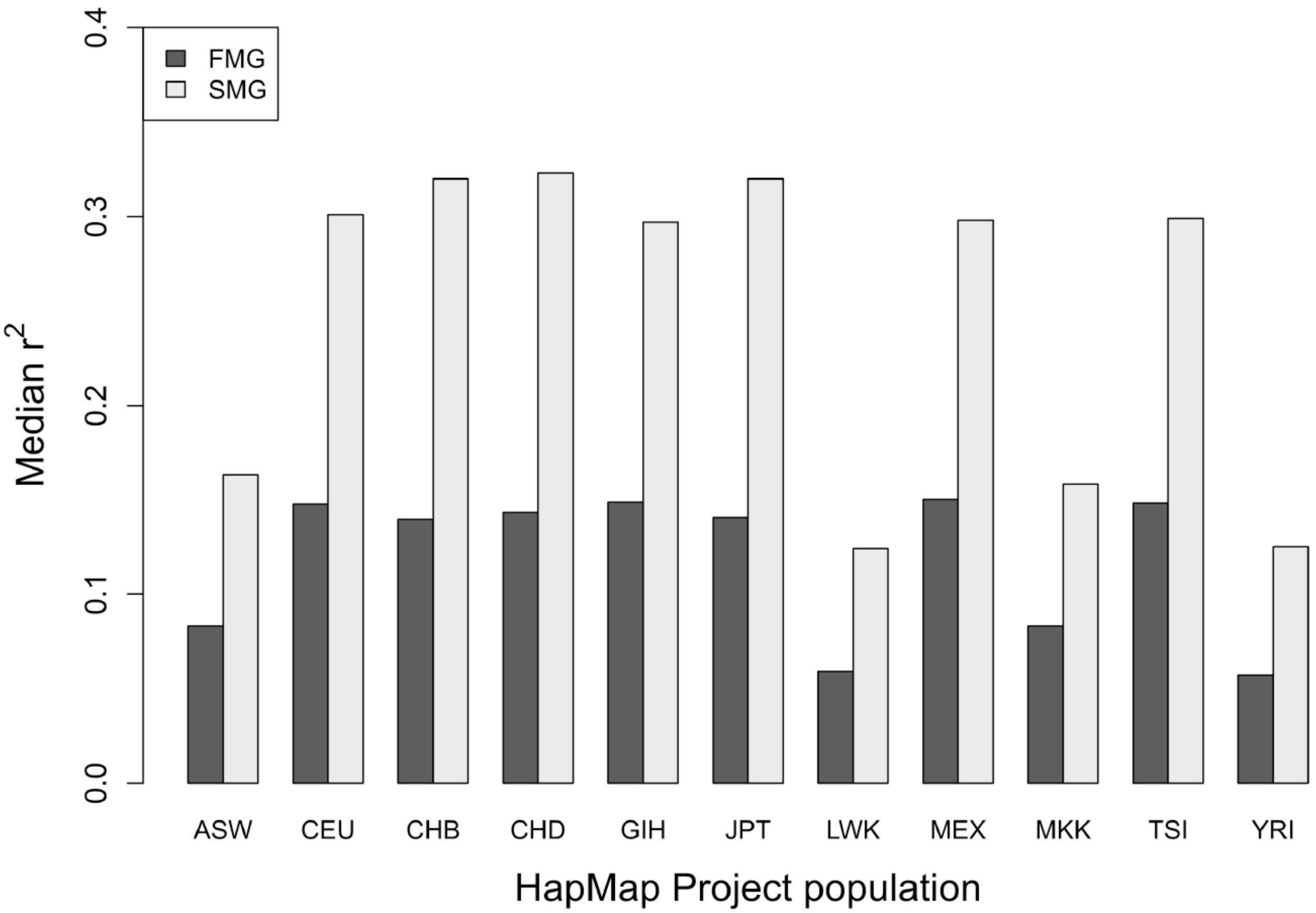

Supplementary Figure S10-7: The bar plots of the SM genes against the FM genes in the LD coefficient r2 for each of the 11 populations of HapMap Project.

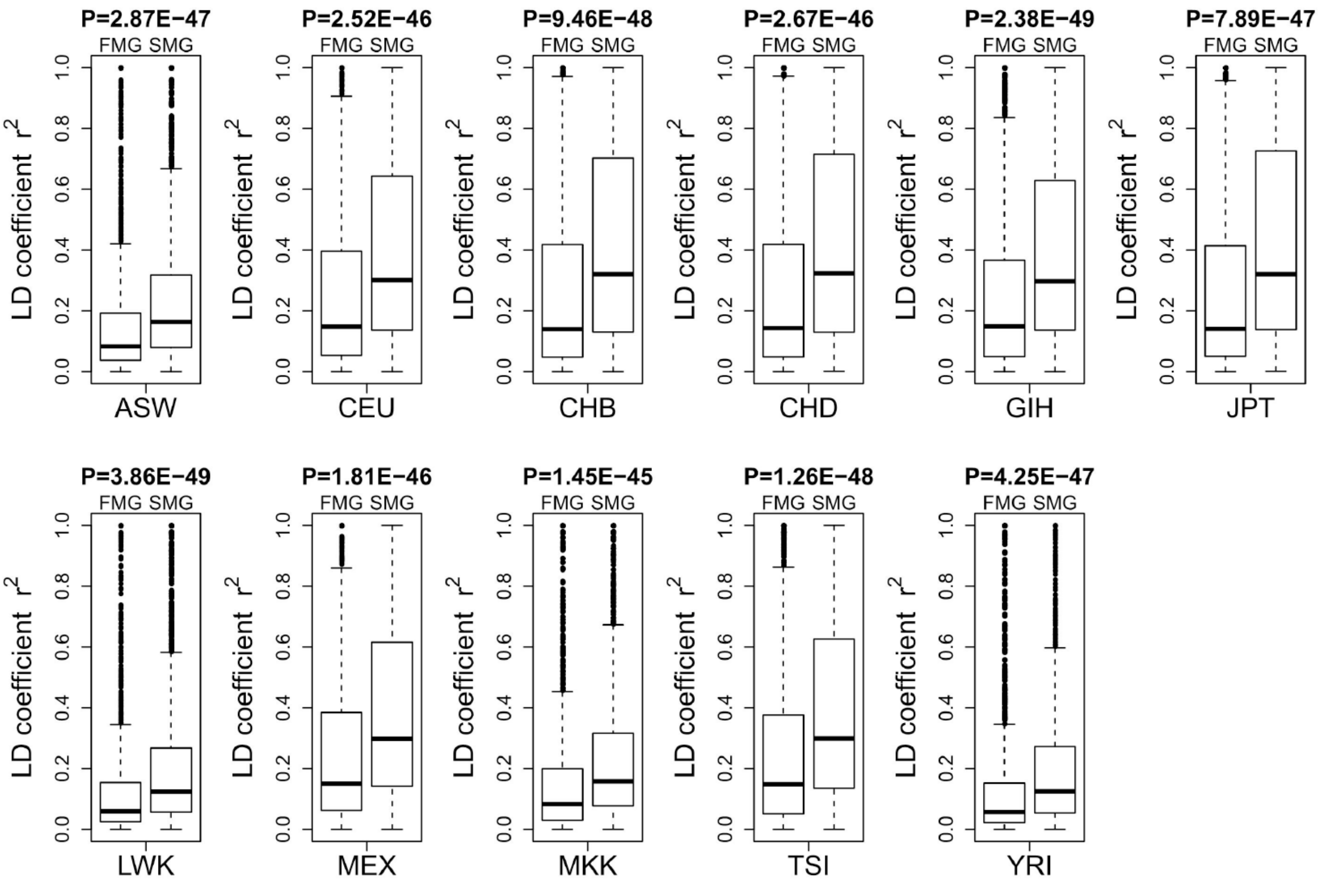

Supplementary Figure S10-8: The box plots of the SM genes against the FM genes in the LD coefficient r2 for each of the 11 populations of HapMap Project.

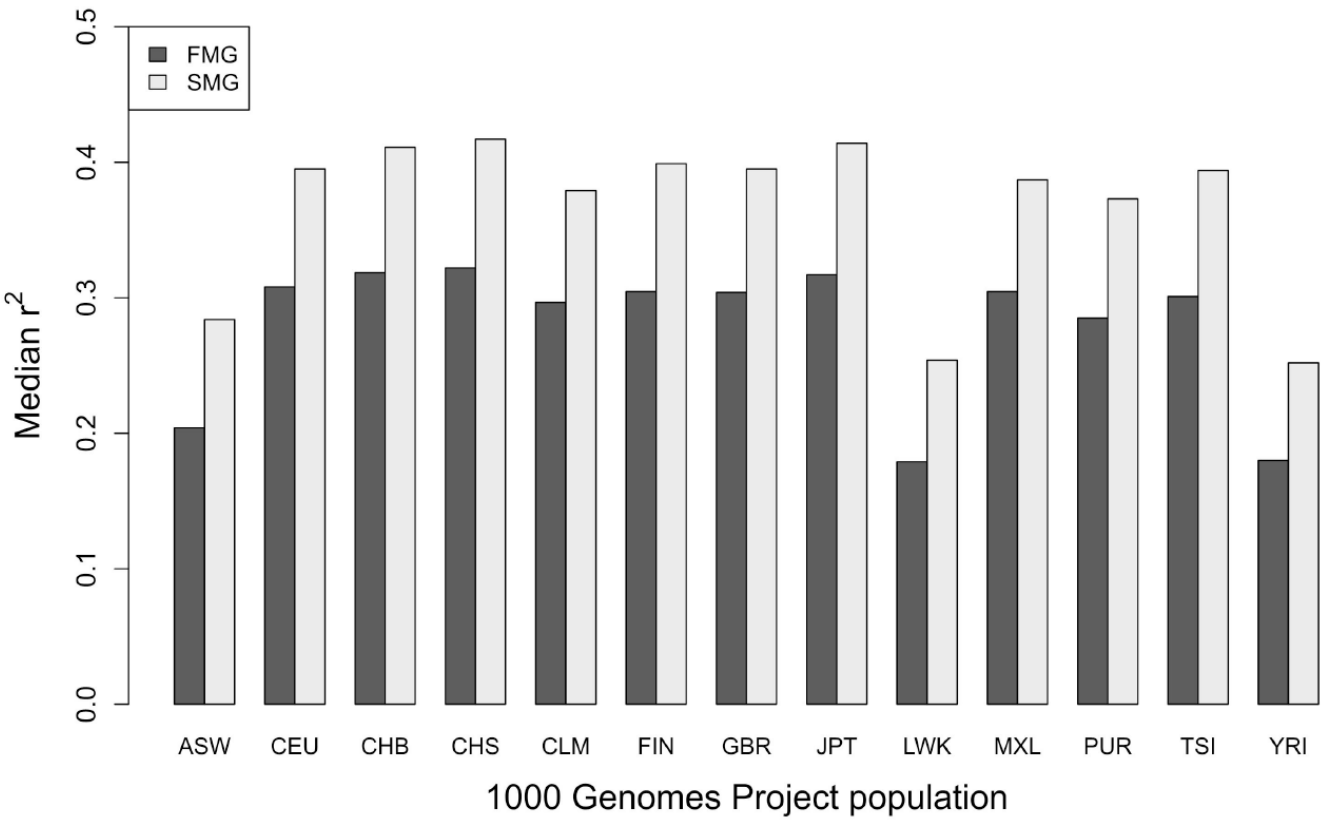

Supplementary Figure S10-9: The bar plots of the SM genes against the FM genes in the LD coefficient r2 for each of the 13 populations of 1000 Genomes Project.

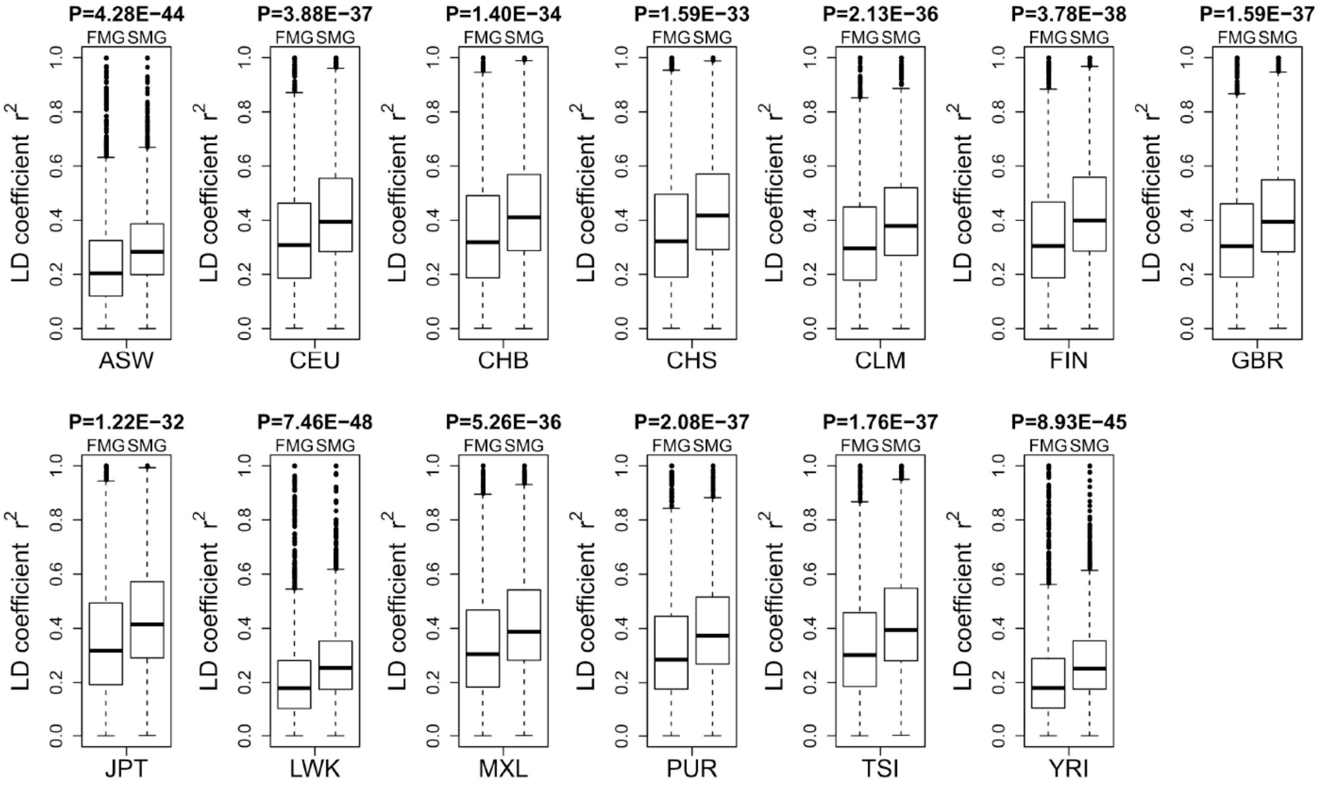

Supplementary Figure S10-10: The box plots of the SM genes against the FM genes in the LD coefficient r2 for each of the 13 populations of 1000 Genomes Project.

Supplementary Table S10-1: Summary statistics for the comparisons of dn/ds in 21 species

Supplementary Table S10-2: Summary statistics for the comparisons of sequence identity in 21 species

Supplementary Table S10-3: Summary statistics for the comparisons of r^2 in HapMap population

Supplementary Table S10-4: Summary statistics for the comparisons of r^2 in 1000 genomes populations

SECTION 11: COMPARISON OF EVOLUTIONARY CONSERVATION BETWEEN SM (TOP 20%) AND FM GENES (BOTTOM 20%) USING ENCODE PROJECT DNA METHYLATION DATA

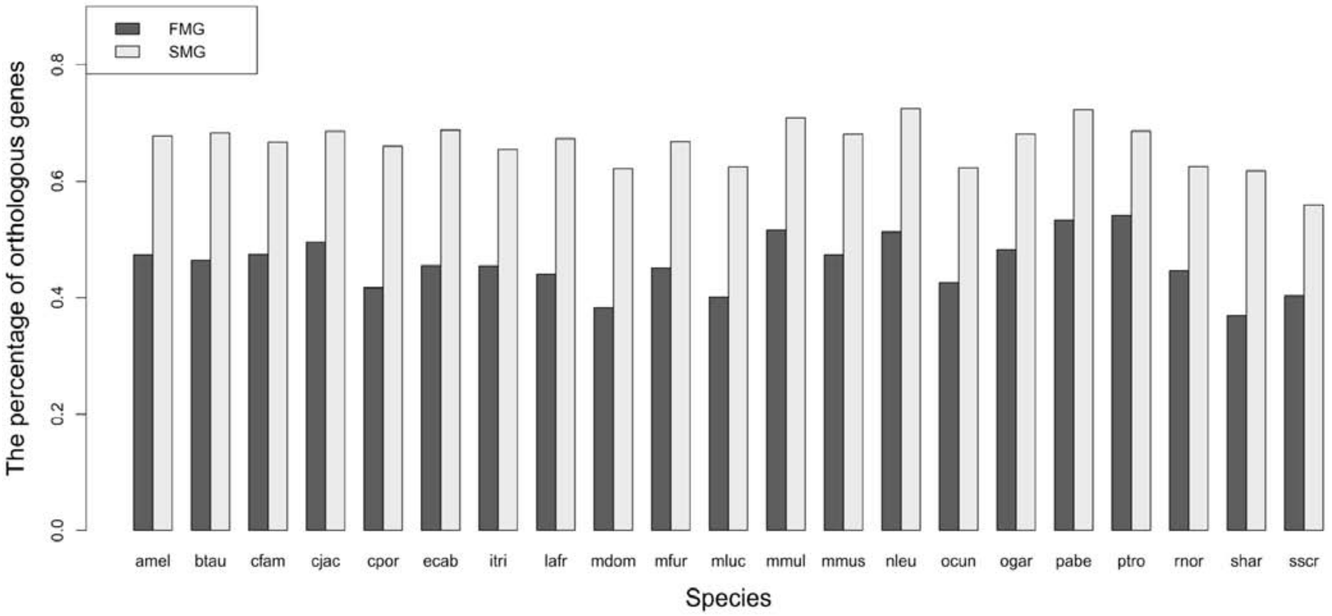

Supplementary Figure S11-1: The bar plots of the SM genes against the FM genes in the percentage of orthologous genes for each of the 21 species.

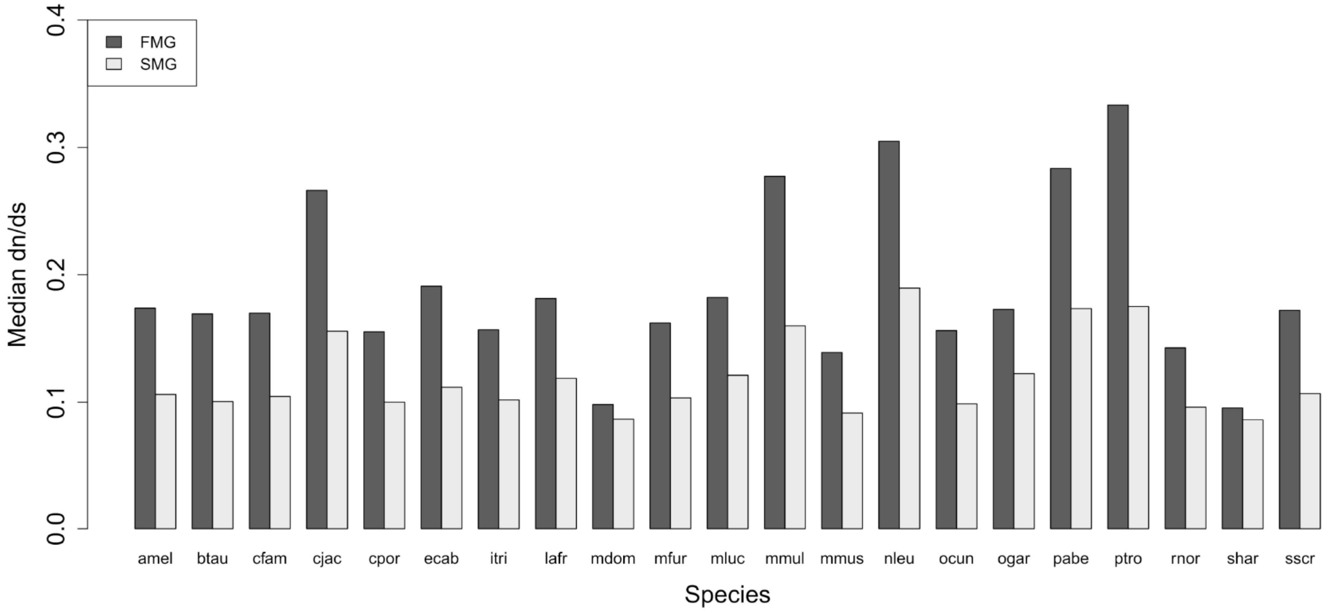

Supplementary Figure S11-2: The bar plots of the SM genes against the FM genes in the evolutionary rate for each of the 21 species.

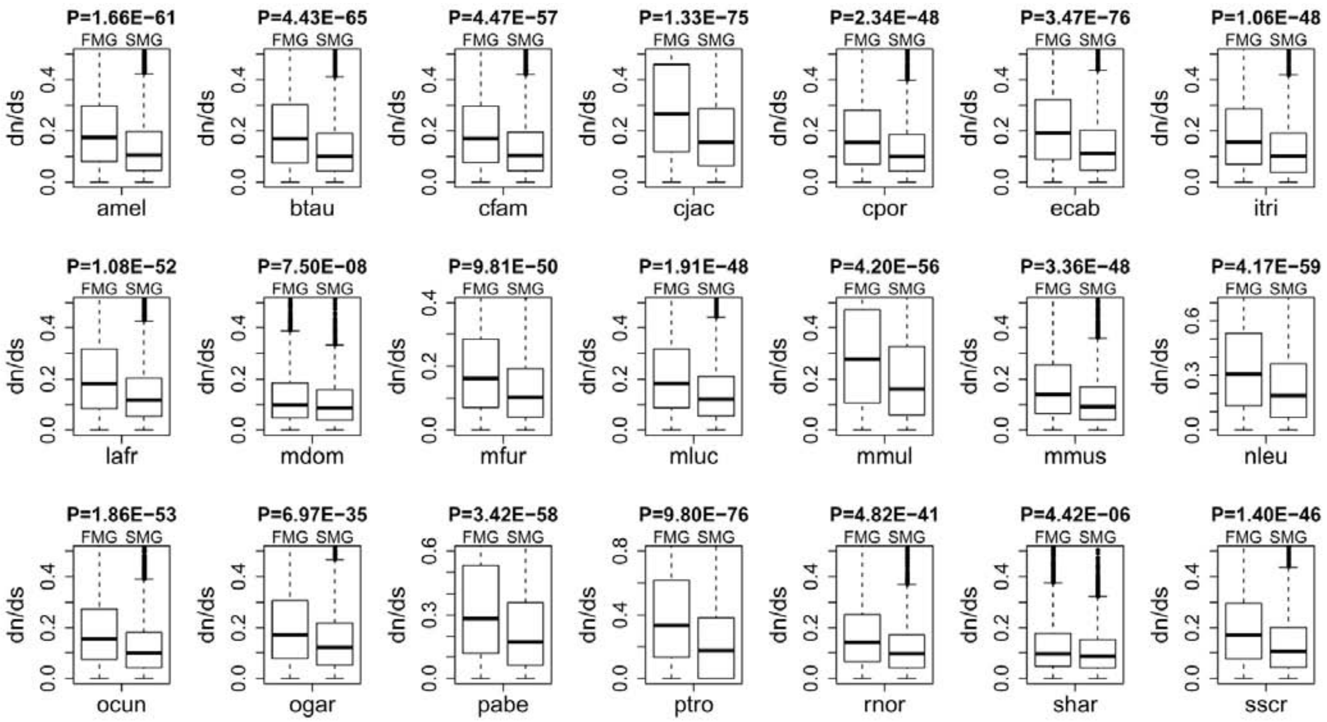

Supplementary Figure S11-3: The box plots of the SM genes against the FM genes in the evolutionary rate for each of the 21 species.

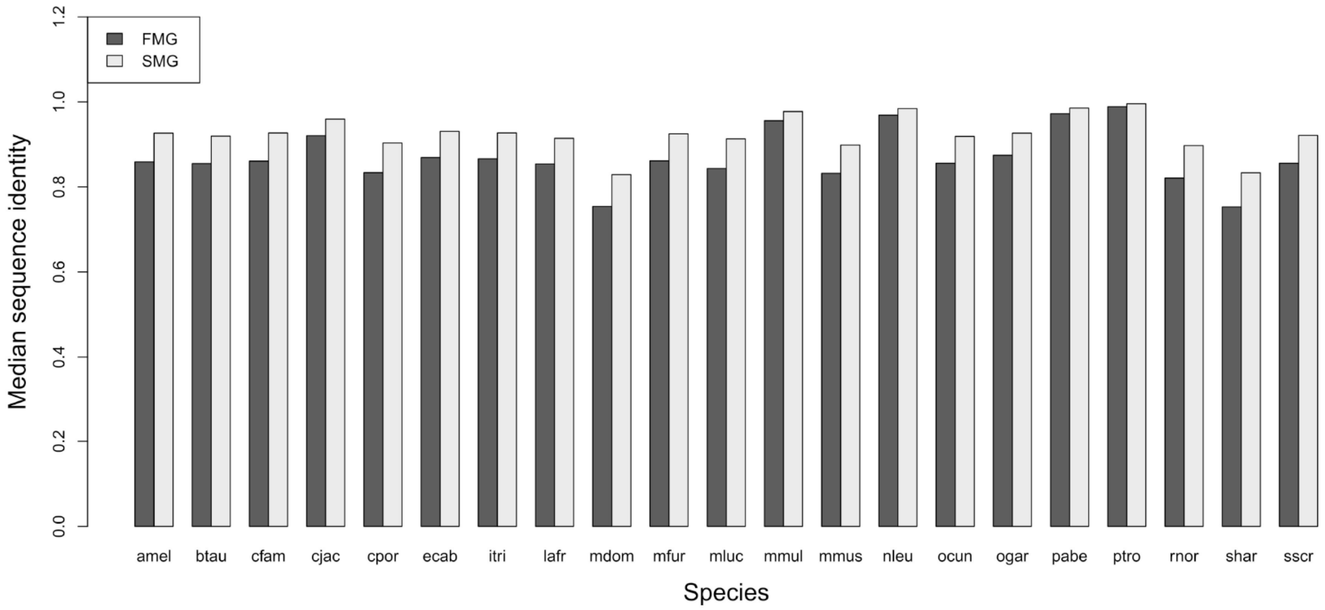

Supplementary Figure S11-4: The box plots of the SM genes against the FM genes in the sequence identity for each of the 21 species.

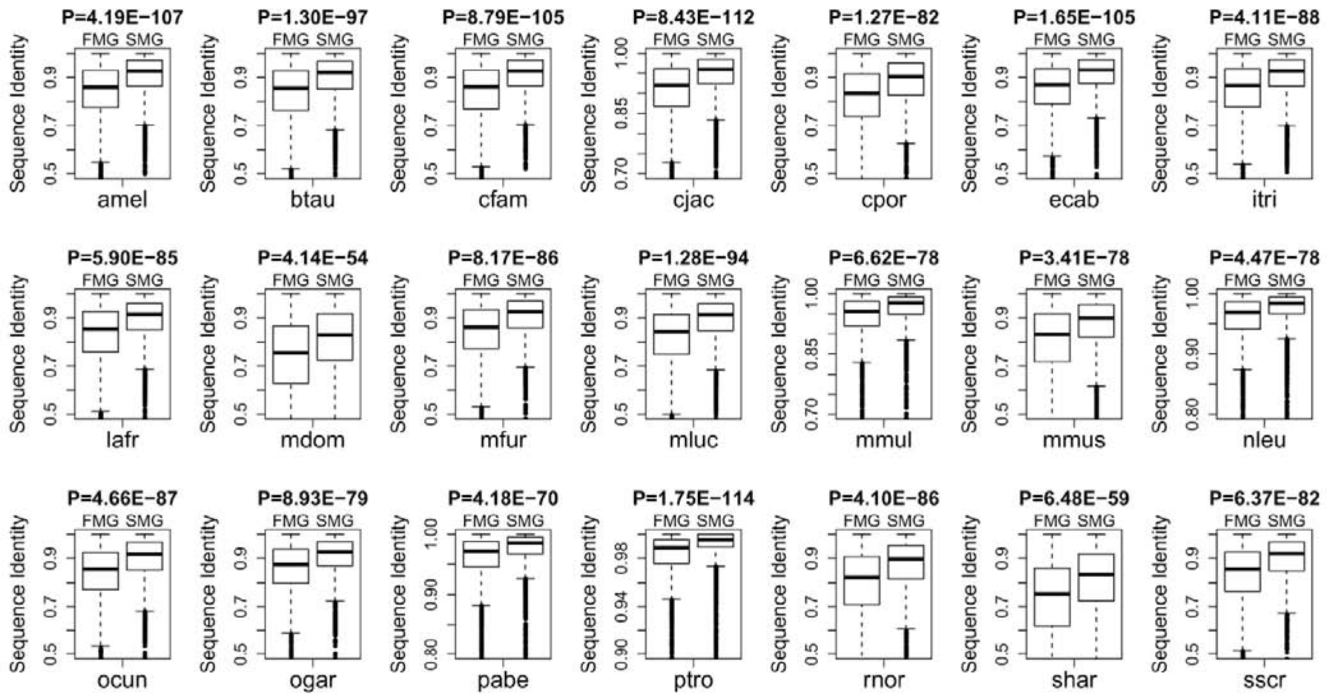

Supplementary Figure S11-5: The bar plots of the SM genes against the FM genes in the sequence identity for each of the 21 species.

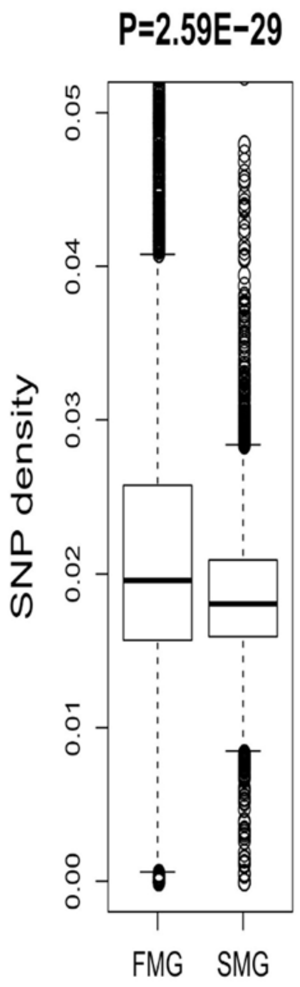

Supplementary Figure S11-6: The box plot of the SM genes against the FM genes in the SNP density.

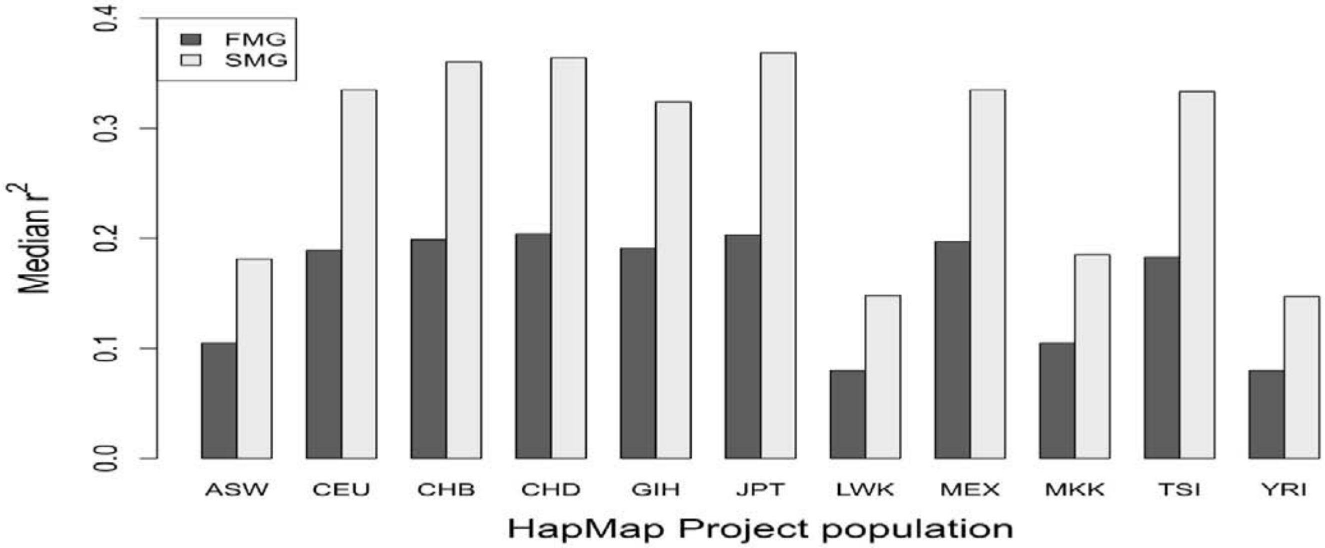

Supplementary Figure S11-7: The bar plots of the SM genes against the FM genes in the LD coefficient r2 for each of the 11 populations of HapMap Project.

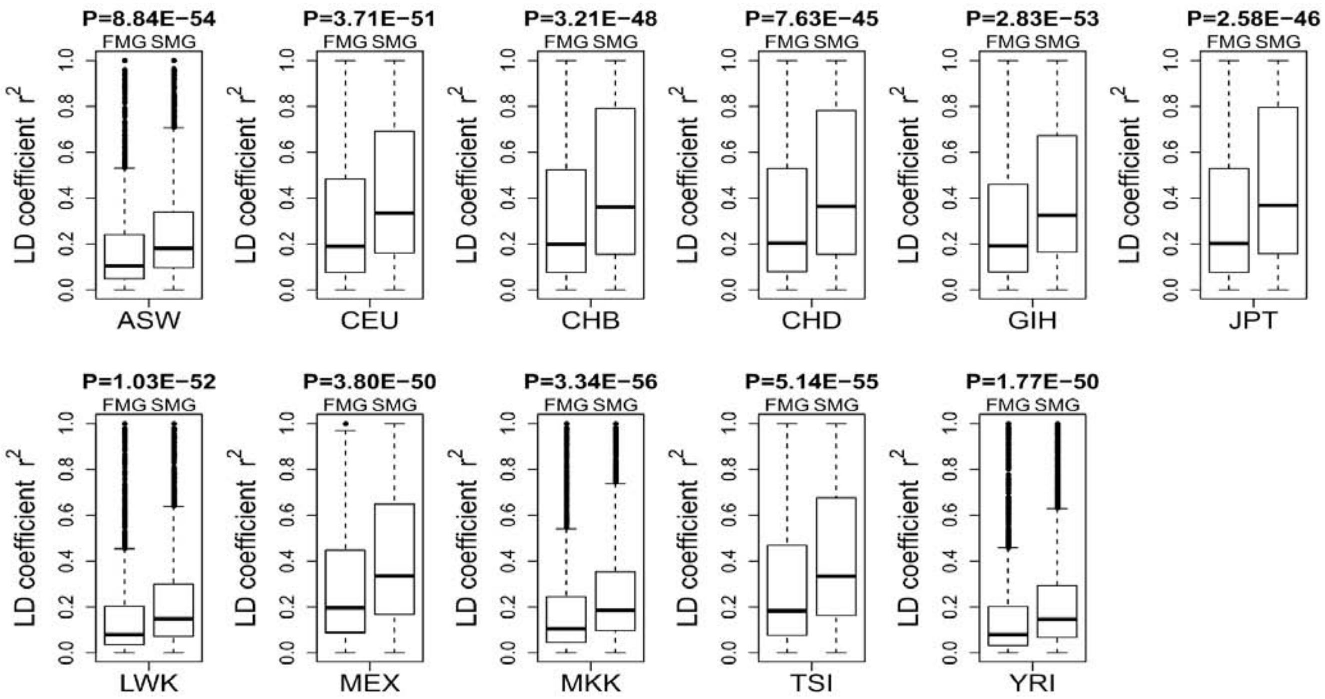

Supplementary Figure S11-8: The box plots of the SM genes against the FM genes in the LD coefficient r2 for each of the 11 populations of HapMap Project.

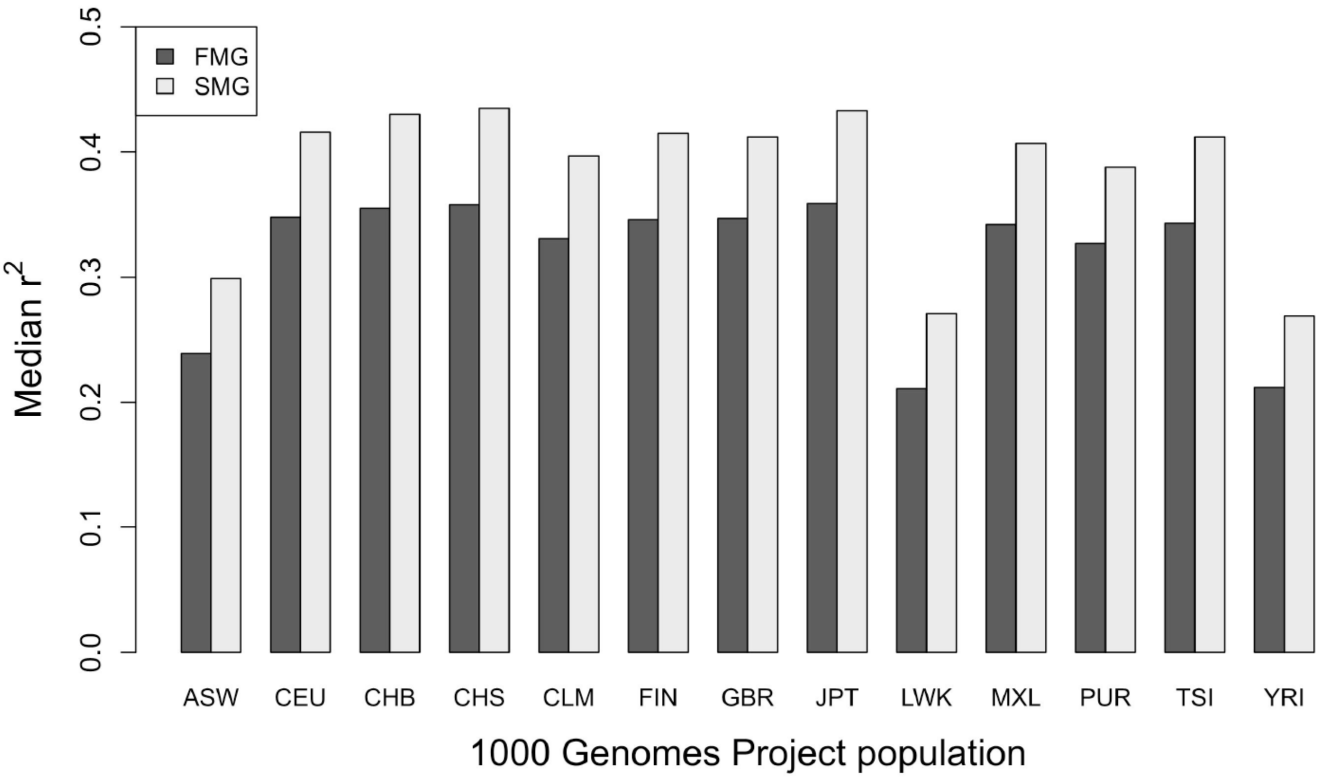

Supplementary Figure S11-9: The bar plots of the SM genes against the FM genes in the LD coefficient r2 for each of the 13 populations of 1000 Genomes Project.

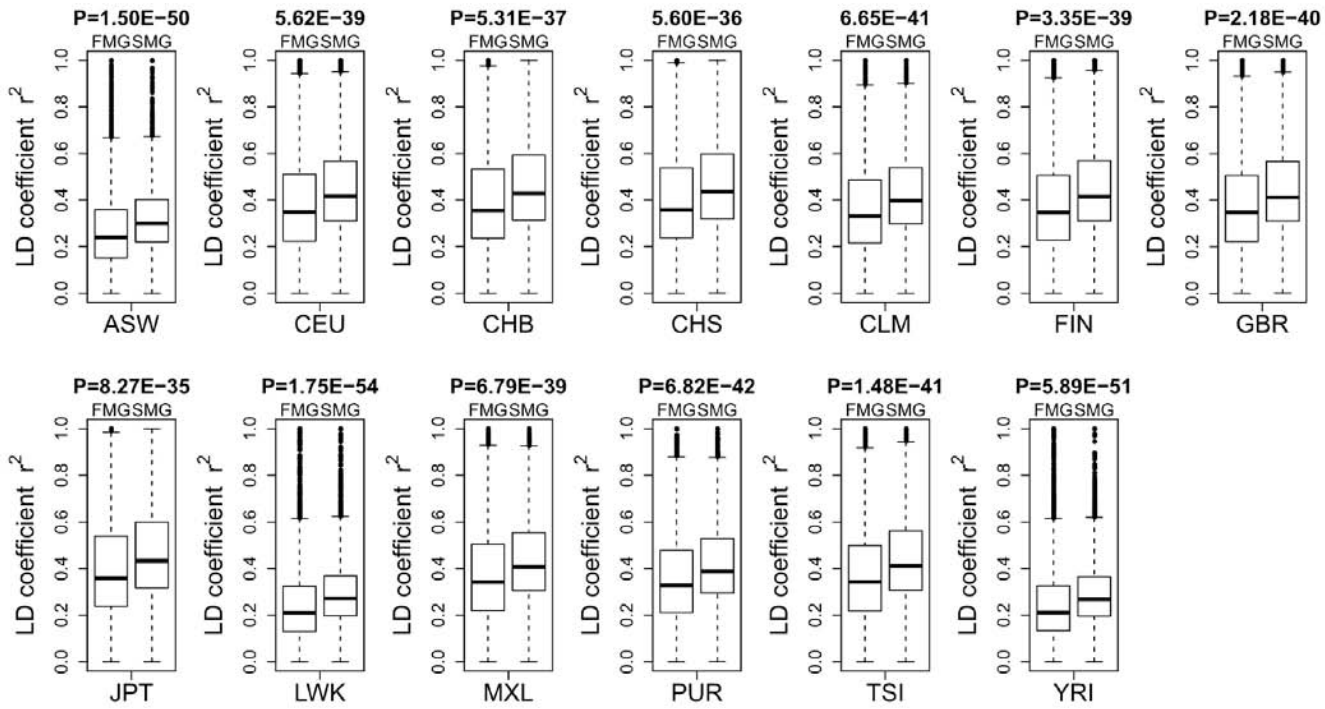

Supplementary Figure S11-10: The box plots of the SM genes against the FM genes in the LD coefficient r2 for each of the 13 populations of 1000 Genomes Project.

Supplementary Table S11-1: Summary statistics for the comparisons of dn/ds in 21 species

Supplementary Table S11-2: Summary statistics for the comparisons of sequence identity in 21 species

Supplementary Table S11-3: Summary statistics for the comparisons of r2 in HapMap population.

Supplementary Table S11-4: Summary statistics for the comparisons of r^2 in 1000 genomes populations
